# Halogen doped graphene quantum dots modulate TDP-43 phase separation and aggregation in the nucleus

**DOI:** 10.1038/s41467-024-47167-x

**Published:** 2024-04-06

**Authors:** Hong Zhang, Huazhang Guo, Danni Li, Yiling Zhang, Shengnan Zhang, Wenyan Kang, Cong Liu, Weidong Le, Liang Wang, Dan Li, Bin Dai

**Affiliations:** 1 School of Sensing Science and Engineering, School of Electronic Information and Electrical Engineering, Shanghai Jiao Tong University, Shanghai, 200240 China; 2 Institute of Nanochemistry and Nanobiology, School of Environmental and Chemical Engineering, Shanghai University, 99 Shangda Road, Baoshan District, Shanghai, 200444 PR China; 3 Interdisciplinary Research Center on Biology and Chemistry, Shanghai Institute of Organic Chemistry, Chinese Academy of Sciences, Shanghai, 201210 China; 4 Department of Neurology and Institute of Neurology, Ruijin Hospital, Shanghai Jiao Tong University School of Medicine, Shanghai, 200025 China; 5 Department of Neurology, Ruijin Hainan Hospital, Shanghai Jiao Tong University School of Medicine (Boao Research Hospital), Hainan, 571434 China; 6 Shanghai University of Medicine and Health Sciences Affiliated Zhoupu Hospital, Shanghai, 201318 China; 7 Center for Clinical and Translational Medicine, Shanghai University of Medicine and Health Sciences, Shanghai, 201318 China; 8 Key Laboratory for the Genetics of Developmental and Neuropsychiatric Disorders (Ministry of Education), Bio-X Institutes, Shanghai Jiao Tong University, Shanghai, 200030 China; 9 Bio-X-Renji Hospital Research Center, Renji Hospital, School of Medicine, Shanghai Jiao Tong University, Shanghai, 200240 China; 10 Zhangjiang Institute for Advanced Study, Shanghai Jiao Tong University, Shanghai, 200240 China

**Keywords:** Protein aggregation, Intrinsically disordered proteins, Quantum dots

## Abstract

TDP-43 is implicated in the dynamic formation of nuclear bodies and stress granules through phase separation. In diseased states, it can further condense into pathological aggregates in the nucleus and cytoplasm, contributing to the onset of amyotrophic lateral sclerosis. In this study, we evaluate the effect of graphene quantum dots (GQDs) with different functional groups on TDP-43’s phase separation and aggregation in various cellular locations. We find that halogen atom-doped GQDs (GQDs-Cl, Cl-GQDs-OH) penetrate the nuclear envelope, inhibiting the assembly of TDP-43 nuclear bodies and stress granules under oxidative stress or hyperosmotic environments, and reduce amyloid aggregates and disease-associated phosphorylation of TDP-43. Mechanistic analysis reveals GQDs-Cl and Cl-GQDs-OH modulate TDP-43 phase separation through hydrophobic and electrostatic interactions. Our findings highlight the potential of GQDs-Cl and Cl-GQDs-OH in modulating nuclear protein condensation and pathological aggregation, offering direction for the innovative design of GQDs to modulate protein phase separation and aggregation.

## Abstract (web summary)

Modulating amyloid protein phase separation and fibrilization may help in addressing neurodegenerative diseases. This study demonstrates that halogen-doped graphene quantum dots can modulate these processes in TDP-43 in both nucleus and cytoplasm.

## Introduction

Liquid-liquid phase separation (LLPS) is an essential mechanism that governs the dynamic formation of membraneless organelles (MLOs), crucial for regulating various physiological functions^1^. In certain pathological conditions, specific proteins may undergo a phase transition from liquid to solid state, resulting in the creation of deleterious amyloid aggregates that are intricately linked to various neurodegenerative diseases (NDs)^2^. TAR DNA-binding protein 43 (TDP-43) is known to form both intranuclear and cytoplasmic aggregates in age-related NDs, including amyotrophic lateral sclerosis (ALS), frontotemporal dementia (FTD), and Alzheimer’s disease (AD)^3–5^.

TDP-43, primarily localized within the nucleus, has the ability to traffic between the nucleus and cytoplasm^6^. It consists of functional domains such as a nuclear localization signal (NLS), a nuclear export signal (NES), two conventional RNA recognition motifs (RRMs), and a C-terminal low complexity (LC) domain filled with disease-associated mutations^7^. Acting as a vital gene expression regulator, TDP-43 is engaged in various RNA processes, and under stress, it may form liquid-like droplets that could transition to solid-like states^8,9^. Such behavior posits that LLPS-driven TDP-43 aggregation might underlie NDs^10^. While many studies have explored cytoplasmic aspects of TDP-43, evidence also points to nuclear aggregation in brain areas affected by FTD and AD^11–14^. Moreover, previous studies show that TDP-43 can form dynamic nuclear bodies (NBs) in nucleus modulated by the lncRNA NEAT1 and Hsp70 in response to stress, playing a protective role under normal conditions^10,15,16^. While, the dynamic TDP-43 NBs may be solidified under chronic stress or diseased condition^15,16^. The disruption of TDP-43 functions, particularly through aberrant phase separation and aggregation in the nucleus, may contribute to ALS and FTD pathogenesis^10^. However, the intricacies of nuclear phase separation and aggregate elimination remain somewhat elusive, highlighting a need for in-depth research to pave the way for potential therapeutic interventions.

Graphene quantum dots (GQDs), an emerging bio-nanomaterial noted for their excellent hydrophilicity, low toxicity, and stable fluorescence, have displayed promise in treating a range of human diseases, from diabetes and cancers to NDs^17–19^. Notably, GQDs have demonstrated capabilities to cross the blood-brain barrier, inhibit pathological α-synuclein accumulation, and mitigate dopamine neuron depletion in Parkinson’s disease models^17^. However, despite their exciting potential for intracellular delivery and therapeutic applications^19^, accessing the nucleus remains a challenge due to the nuclear envelope’s regulatory restrictions.

In response to this challenge, our study focuses on the development and synthesis of halogen atom-doped GQDs, aimed at augmenting their cellular permeability. These GQDs hold promise for penetrating the nuclear membrane, opening horizons for the manipulation of nuclear protein phase separation and aggregation. We present evidence that GQDs can modulate the construction of TDP-43 NBs and TDP-43-positive stress granules (SGs) under oxidative stress and hyperosmotic stimuli. Furthermore, we observed the GQDs’ ability to restrain both aggregation and phosphorylation levels within the nucleus and cytoplasm. While these data remain preliminary, it suggests potential roles for GQDs in modulating phase separation and amyloid aggregation of proteins associated with NDs.

## Results

### GQDs-NH_**2**_ and GQDs-OH inhibit TDP-43 condensation in the cytoplasm but not in the nucleus

We firstly sought to examine the ability of amino and hydroxy-functionalized GQDs (GQDs-NH_2_ and GQDs-OH), which was previously found to exhibit a potent activity in preventing condensation of the FUS protein^20^, in modulating phase separation of TDP-43 (Supplementary Fig. 1a, b). As shown in Supplementary Fig. 2, both GQDs-NH_2_ and GQDs-OH effectively infiltrated the liquid-like droplets of TDP-43, thereby successfully inhibiting their formation in a dose-dependent manner (Supplementary Fig. 2a–d). To further determine whether GQDs-NH_2_ and GQDs-OH could also suppress the phase separation of TDP-43 within cells, we transfected the TDP-43 D169G HA plasmid into HeLa cells. The D169G mutation, originally discovered in an isolated sporadic ALS case^21^, induces translocation of TDP-43 into the cytoplasm and formation of SGs under sodium arsenite stress conditions (Supplementary Figs. 2e and 3a, b). Unstimulated TDP-43 D169G transfected HeLa cells form minimal SGs (Supplementary Fig. 2e). However, both GQDs-NH_2_ and GQDs-OH significantly decreased the number of preformed TDP-43 D169G positive SGs after 1 h of incubation (Supplementary Fig. 2e, f). To determine if the decrease in SGs is due to GQDs preventing cytoplasmic mislocalization of TDP-43 D169G, we separated the cytoplasmic and nuclear fractions and measured the expression levels of TDP-43 D169G. The results indicate both GQDs-NH_2_ and GQDs-OH do not affect the distribution of TDP-43 D169G between the cytoplasm and nucleus (Supplementary Fig. 3c, d). However, it is important to note that GQDs-NH_2_ and GQDs-OH were unsuccessful in restricting the formation of NBs induced by arsenite stress in cells overexpressing the TDP-43 HA plasmid (Supplementary Fig. 2g, h), possibly due to their limited ability to penetrate the nucleus compared to their effective entry into the cytoplasm cytoplasmic^20^.

We next evaluate whether GQDs-NH_2_ or GQDs-OH could prevent amyloid fibrillation of TDP-43 LC, which plays a key role in mediating full-length TDP-43 pathological aggregation^22^. Utilizing thioflavin T (ThT) fluorescence assay and negative-staining transmission electron microscopy (TEM), we found that both GQDs demonstrated a dose-dependent inhibition of TDP-43 LC fibrillation (Supplementary Fig. 2i, j). Additionally, we transfected HeLa cells with TDP-43 C-terminal fragments (CTF), a 25 kDa C-terminal fragment of TDP-43 associated with insoluble cytoplasmic aggregates in ALS/FTD patient brain tissue^23^. Notably, post-incubation with the GQDs-NH_2_ or GQDs-OH, a drastic reduction in aggregate formation was observed (Supplementary Fig. 2k, l). Western blot (WB) results showed that GQDs-NH_2_ or GQDs-OH incubation did not influence TDP-43 CTF plasmid expression but did reduce TDP-43 phosphorylation (pTDP-43) (S409/410) levels (Supplementary Fig. 2o, p), a disease-specific marker of TDP-43 aggregates^24^. The TDP-43 K181E mutation, associated with ALS/FTD^25^, triggered the spontaneous formation of large, irregularly shaped TDP-43 nuclear aggregates, even in the absence of stress conditions^16^. Nuclear aggregation of TDP-43 K181E exhibits slower recovery in the fluorescence recovery after photobleaching (FRAP) assay (Supplementary Fig. 4a, b) and is hyperphosphorylated at S409/410 (Supplementary Fig. 4c). Although severe protein aggregation occurs, Caspase 3 staining (Supplementary Fig. 4d) and TUNEL assay (Supplementary Fig. 4e) demonstrate that cells expressing the TDP-43 K181E plasmid do not undergo apoptosis. However, in the case of nuclear aggregation, examined through transfection of HeLa cells with TDP-43 K181E plasmid, both GQDs were ineffective (Supplementary Fig. 2m, n). Moreover, WB analysis revealed that the presence of GQDs-NH_2_ or GQDs-OH did not impact TDP-43 K181E plasmid expression or pTDP-43 (S409/410) levels (Supplementary Fig. 2m, n, q, r). Therefore, to more effectively regulate the protein LLPS in the nucleus, it is suggested that there is a necessity to design modulators with enhanced nuclear permeability.

### Design and characterization of nucleus-penetrating halogen atom-doped GQDs

**Fig. 1:**
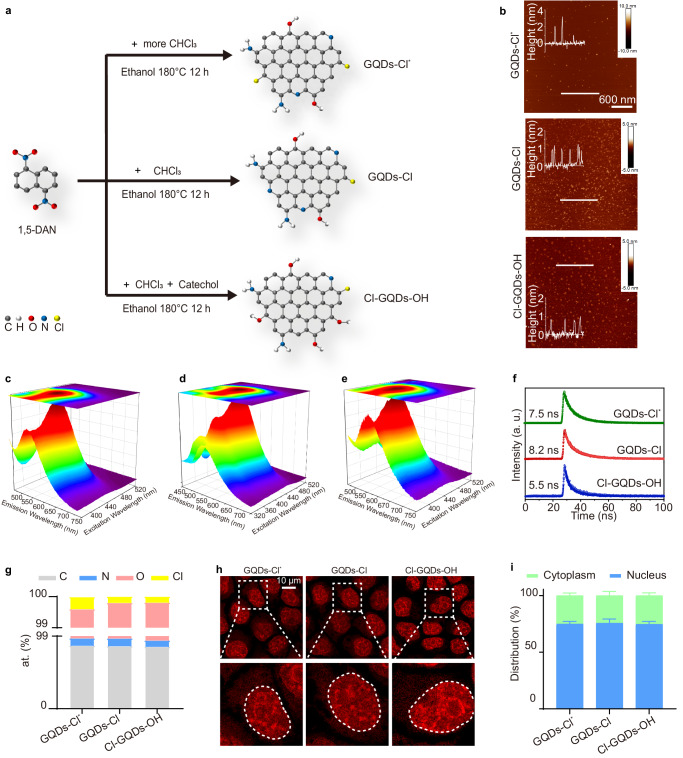
Synthesis and structural characterization of halogen atom-doped GQDs. **a** Schematic representation of the synthetic process for the three types of halogen atom-doped GQDs. **b** AFM images of the three types of halogen atom-doped GQDs with height profiles along the white lines. The imaging was independently repeated 3 times with similar observations. **c**–**e** 3D spectra of GQDs-Cl*, GQDs-Cl and Cl-GQDs-OH, respectively. **f** Time-resolved PL spectrum of the halogen atom-doped GQDs. **g** Comparative histogram of the elemental content of XPS spectra. **h** Confocal fluorescence images for all three types of halogen atom-doped GQDs. The imaging was independently repeated 3 times with similar observations. **i** The graph shows the percentage distribution of halogen atom-doped GQDs within the nucleus and cytoplasm compartments. Data correspond to the mean ± SD, *n* = 3. Source data are provided as a Source Data file.

Previous studies demonstrated that the introduction of chlorine atoms into molecules could increase nucleus uptake^26,27^. Thus, we designed and synthesized chlorine-functionalized GQDs (GQDs-Cl^*^) using a previously established method involving a one-step molecular fusion process under solvothermal conditions with the active molecular precursor, 1,5-diaminonaphthalene (DAN). To decrease the hydrophobicity of GQDs-Cl^*^, we synthesized a variant with reduced chloroform content (GQDs-Cl). Furthermore, GQDs-Cl functionalized with hydroxyl groups (Cl-GQDs-OH) was synthesized by introducing catechol into the system (Fig. 1a and Table 1). The thickness of the GQDs was determined using atomic force microscopy (AFM) (Fig. 1b). All halogen atom-doped GQDs exhibited significant visible light absorption (Fig. 1c–e). Interestingly, the photoluminescence (PL) spectrum of GQDs remained largely unchanged by modulating the solvent volume ratio of CHCl_3_ or catechol introduction. These results suggest that the addition of chlorine and hydroxyl groups primarily influenced the light absorption characteristics of GQDs without affecting the PL and photoluminescence excitation (PLE) maxima (Fig. 1c–e). The intrinsic PL properties of the three synthesized GQDs types are further exhibited in their respective PL decay curves (Fig. 1f). These curves demonstrate a monoexponential decay, characterized by a single, prolonged lifetime. Structural characterization of the GQDs (GQDs-Cl^*^, GQDs-Cl, Cl-GQDs-OH) by Raman spectra confirmed their high graphitization (Supplementary Fig. 5a). Notably, Cl-GQDs-OH showed fewer defects, as evidenced by the stronger G to D intensity ratio (Supplementary Fig. 5a). The FT-IR revealed similar spectroscopic characteristics across the three samples (Supplementary Fig. 5b). X-ray photoelectron spectroscopy (XPS) analysis was conducted to confirm the surface groups of the GQDs (Fig. 1g and Supplementary Fig. 5c–e). The high-resolution spectra indicated the formation of covalent bonds between chloride ions and carbon (Supplementary Fig. 5c4, d4, e4). These data indicate that the synthesized GQDs were multi-doped, with abundant -NH_2_ and -OH functional groups, as well as rare -Cl functional groups. Confocal fluorescence microscopy revealed the cellular uptake of GQDs in HeLa cells after a 20-min incubation and two PBS washes (Fig. 1h). Interestingly, a higher GQDs density was observed in the nucleus compared to the cytoplasm (Fig. 1i). And the higher amount of the chlorine atom, the greater the uptake of GQDs into the nucleus (Supplementary Fig. 5f). In addition, cytotoxicity assays were conducted to assess the impact of GQDs on cell viability. Cells were exposed to GQDs at concentrations ranging from 20 to 100 mg/l for variable incubation times of 6 to 12 h (Supplementary Fig. 5g–i). Results showed negligible cytotoxicity for GQDs-Cl and Cl-GQDs-OH, signifying their potential safety for subsequent experiments and prompting us to focus on these two GQDs types (GQDs-Cl, Cl-GQDs-OH).

**Table 1:** Proportions of reagents used in the synthesis of GQDs

Sample	1,5-diaminonaphthalene/g	CHCl_3_/ml	Ethanol/ml	Catechol/g
GQDs-Cl*	0.1	3	7	0
GQDs-Cl	0.1	1	9	0
Cl-GQDs-OH	0.1	1.5	8.5	0.025
GQDs	0.1	0	10	0

### Halogen atom-doped GQDs modulate the phase separation of TDP-43 in vitro through electrostatic and hydrophobic interactions

**Fig. 2:**
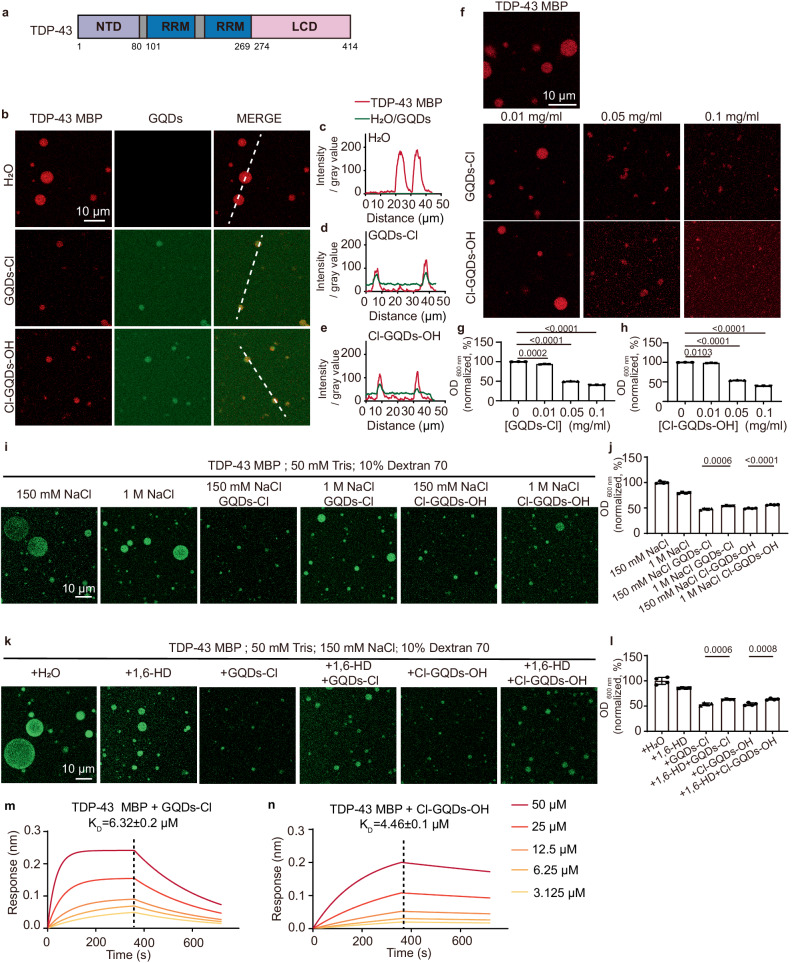
Halogen atom-doped GQDs inhibit TDP-43 phase separation in vitro. **a** Domain architecture of TDP-43. **b** Fluorescent images show TDP-43 MBP in the presence and absence of GQDs-Cl or Cl-GQDs-OH. TDP-43 MBP at a concentration of 20 μM undergoes LLPS within a buffer (50 mM Tris, pH 7.5, 150 mM NaCl, 10% Dextran 70), subsequently combined with 0.01 μg/μl GQDs-Cl or Cl-GQDs-OH. Scale bar, 10 μm. The imaging was independently repeated 3 times with similar observations. **c**–**e** Intensity profiles evaluating the colocalization of GQDs with TDP-43 MBP. **f** Fluorescence images provide representative views of 40 μM TDP-43 MBP undergoing phase separation in the presence of varying GQDs-Cl or Cl-GQDs-OH concentrations. Scale bar, 10 μm. The imaging was independently repeated 3 times with similar observations. **g**, **h** Turbidity measurements show the turbidity of TDP-43 MBP phase separation in the presence of GQDs-Cl or Cl-GQDs-OH. Data correspond to the mean ± SD, *n* = 3, two-tailed unpaired *t*-test. **i** Representative fluorescence images of 40 μM TDP-43 MBP undergoing phase separation with 50 mM Tris, pH 7.5, 1 M NaCl, 10% Dextran 70 and 0.1 μg/μl GQDs-Cl or Cl-GQDs-OH. Scale bar, 10 μm. The imaging was independently repeated 3 times with similar observations. **j** Turbidity measurement of 40 μM TDP-43 MBP as shown in image (**h**). Data correspond to mean ± SD, *n* = 4, two-tailed unpaired *t*-test. **k** Fluorescence image of TDP-43 MBP in the presence and absence of 1,6-HD and GQDs-Cl or Cl-GQDs-OH. Scare bar, 10 μm. The imaging was independently repeated 3 times with similar observations. **l** Turbidity measurement of 40 μM TDP-43 MBP as shown in image (**j**). Data correspond to mean ± SD, *n* = 4, two-tailed unpaired *t*-test. **m**, **n** Kinetic binding curves of TDP-43 MBP with a concentration gradient of GQDs-Cl or Cl-GQDs-OH. The association and dissociation profiles are divided by a vertical black dotted line. Source data are provided as a Source Data file.

Given the significant impact observed with GQDs-NH_2_ and GQDs-OH on the phase separation of TDP-43 protein (Supplementary Fig. 2), we extended our investigation to explore the potential of the halogen atom-doped GQDs (GQDs-Cl, Cl-GQDs-OH) in modulating the LLPS of TDP-43 protein. Notably, both GQDs-Cl and Cl-GQDs-OH demonstrated a substantial ability to permeate TDP-43 liquid-like droplets spontaneously (Fig. 2a–e). Additionally, it was observed that both GQDs had a dose-dependent inhibitory effect on the formation of TDP-43 liquid droplets (Fig. 2f–h), implying that both GQDs-Cl and Cl-GQDs-OH have the potential to regulate TDP-43 protein phase separation. SDS-PAGE analysis confirms that the TDP-43 MBP protein remains intact in our experimental conditions (Supplementary Fig. 6a).

To gain deeper insights into the interactions underlying halogen atom-doped GQDs and TDP-43 protein, we explored both charge-based and hydrophobic factors. Charge-based interactions can be disrupted through the addition of salt^28,29^. However, high salt concentrations significantly impeded the anti-LLPS capability of halogen atom-doped GQDs on TDP-43, indicating the involvement of electrostatic interactions in the binding mechanism between GQDs and TDP-43 protein (Fig. 2i, j). Furthermore, our investigation extended to hydrophobic interactions by introducing 1,6-hexanediol (1,6-HD), a known disruptor of hydrophobic interactions^30,31^, into the phase-separated system of TDP-43 and GQDs. Upon the addition of 1,6-HD to the system, a reduction in the inhibitory effect of GQDs-Cl or Cl-GQDs-OH on phase separation was observed (Fig. 2k, l). Consequently, these results underscore the involvement of both hydrophobic and electrostatic interactions in the binding mechanism between halogen atom-doped GQDs and TDP-43 protein. The mean zeta potentials of GQDs-Cl and Cl-GQDs-OH were found to be −2.58 mV and −9.00 mV, respectively (Supplementary Fig. 7a).

To accurately quantify the binding affinity between GQDs and TDP-43 protein, we employed biolayer interferometry (BLI) assays. TDP-43 protein was immobilized on the biosensor and incubated with GQDs-Cl or Cl-GQDs-OH at various concentrations. Both types of GQDs demonstrated binding capabilities to TDP-43 monomers, and the dissociation constant (*K*_D_) values, a measure of binding affinity, were determined to be in the range from 4 μM to 6 μM (Fig. 2m, n). BLI assays show that TDP-43 interacts with halogen atom-doped GQDs, showing *K*_D_ values of 4.80 μM and 1.35 μM (Supplementary Fig. 8a, b). These values are comparable to the *K*_D_ values observed for the interaction between TDP-43 MBP and halogen atom-doped GQDs (Fig. 2m, n). This suggests that the MBP tag has a minimal impact on the interaction between TDP-43 and GQDs. These findings further emphasize the consistent binding affinities and the potential regulatory roles of GQDs-Cl and Cl-GQDs-OH in TDP-43 phase separation.

### Halogen atom-doped GQDs prevent formation of TDP-43 granules and disassemble them in cells

**Fig. 3:**
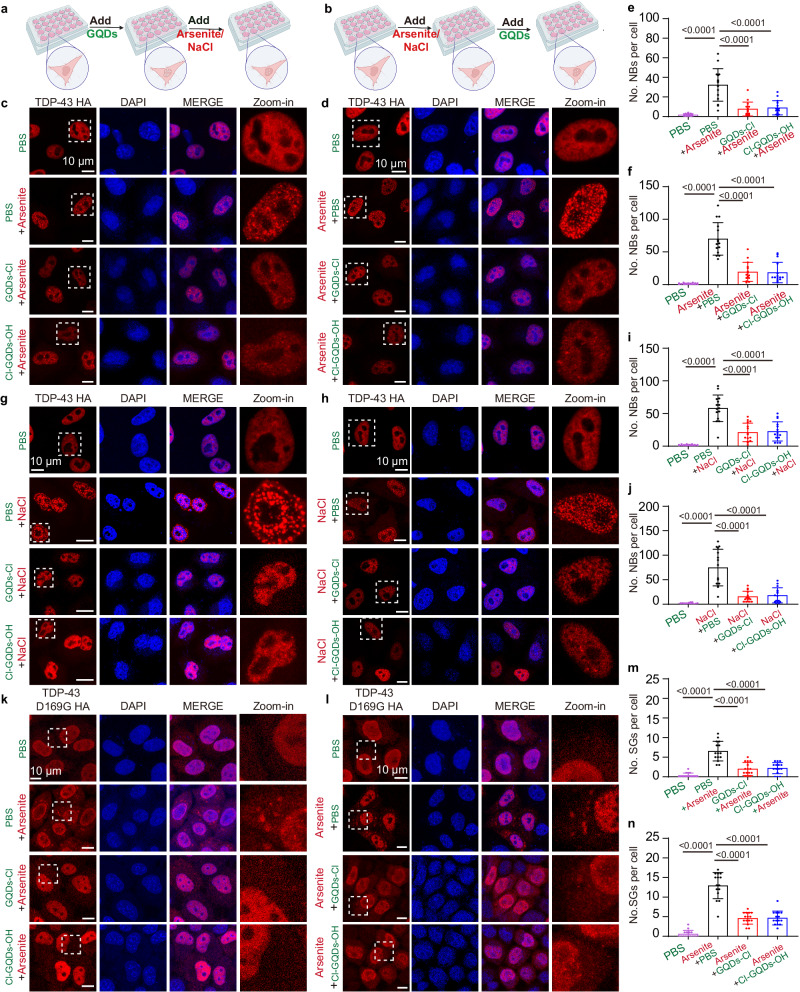
Halogen atom-doped GQDs disassemble preformed TDP-43 granules and prevent their formation in the nucleus and cytoplasm. **a**, **b** Schematic diagram of the TDP-43 granules inhibition and disassembly assay. Created with BioRender.com **c** Representative confocal images of HeLa cells transfected with TDP-43 HA. Cells were treated with 0.1 μg/μl of GQDs for 1 h, followed by 250 μM arsenite for 1 h. Scale bar, 10 μm. **d** Representative confocal images of HeLa cells transfected with TDP-43 HA. Cells were treated with 250 μM sodium arsenite for 1 h, followed by 0.1 μg/μl of GQDs for 1 h. Scale bar, 10 μm. **e**, **f** Quantitative analysis of the number of NBs in nucleus per cell for images (**c**, **d**). Data shown are means ± SD, *n* = 15, two-tailed unpaired *t*-test. **g** Representative confocal images of HeLa cells transfected with TDP-43 HA. Cells were treated with 0.1 μg/μl of GQDs for 1 h, followed by 300 mM NaCl for 1 h. Scale bar, 10 μm. **h** Representative confocal images of HeLa cells transfected with TDP-43 HA. Cells were treated with 300 mM NaCl for 1 h, followed by 0.1 μg/μl of GQDs for 1 h. Scale bar, 10 μm. **i**, **j** Quantitative analysis of the number of NBs in nucleus per cell for image (**g**, **h**). Data shown are means ± SD, *n* = 15, two-tailed unpaired *t*-test. **k** Representative confocal images of HeLa cells transfected with TDP-43 D169G HA. Cells were treated with 0.1 μg/μl of GQDs for 1 h and then treated with 250 μM arsenite for 1 h. Scale bar, 10 μm. **l** Representative confocal images of HeLa cells transfected with TDP-43 D169G HA. Cells were treated with 250 μM sodium arsenite for 1 h and then treated with 0.1 μg/μl of GQDs for 1 h. Scale bar, 10 μm. **m**, **n** Quantitative analysis of the number of SGs in cytoplasm per cell for image (**k**, **l**). Data shown are means ± SD, *n* = 15, two-tailed unpaired *t*-test. The imaging for (**c**, **d**, **g**, **h**, **k**, **l**) was independently repeated 3 times with similar observations. Source data are provided as a Source Data file.

We subsequently examined whether halogen atom-doped GQDs could modulate TDP-43 phase separation in the nucleus. We transfected HeLa cells with TDP-43 HA encoding plasmids. Then, the cells were pretreated with GQDs-Cl or Cl-GQDs-OH for 1 h before a 1-h sodium arsenite stress exposure (Fig. 3a). Notably, the pretreatment with GQDs led to a significant reduction in the number of TDP-43 NBs upon stress, implicating that both GQDs-Cl and Cl-GQDs-OH possess the capability to mitigate the formation of TDP-43 NBs (Fig. 3c, e). Moreover, both GQDs-Cl and Cl-GQDs-OH effectively reduced the number of preformed TDP-43 NBs after 1 h of incubation (Fig. 3b, d, f). It is worth noting that GQDs alone, in the absence of sodium arsenite, did not induce the formation of TDP-43 NBs (Supplementary Fig. 9a, b). To ensure halogen atom-doped GQDs does not affect the expression of TDP-43, we conducted a WB assay. The results demonstrate that halogen atom-doped GQDs do not alter the expression level of TDP-43 protein (Supplementary Fig. 9c, d). Meanwhile, we conduct toxicity tests to examine potential cytotoxic effects under non-stress conditions, arsenite stimulation, and halogen atom-doped GQDs supplementation post-arsenite stimulation. Our results show that neither treatment exhibited obvious toxicity (Supplementary Fig. 9e). Taken together, these observations suggest that halogen atom-doped GQDs exhibit potent preventive and disassembling effects on TDP-43 NBs induced by oxidative stress.

Previous studies have highlighted the propensity of TDP-43 to assemble into NBs under hypertonic solutions^32^. Consistently, we found GQDs-Cl and Cl-GQDs-OH to efficiently prevent and disassemble hypertonic stimulation-induced TDP-43 NBs (Fig. 3g–j). These findings reveal the potent activity of the GQDs in preventing and disassembling stress-induced TDP-43 NBs under different types of stress. Importantly, GQDs-Cl and Cl-GQDs-OH proved to be equally effective in preventing and disassembling TDP-43 NBs induced by hypertonic stimulation in Neuro 2a (N2a) cells, thereby extending the applicability of the GQDs across different cell types (Supplementary Fig. 10a–f).

Although primarily localized in the nucleus, a small fraction of GQDs-Cl and Cl-GQDs-OH was detected in the cytoplasm (Fig. 1h, i). This observation prompted us to investigate the potential of GQDs in inhibiting or depolymerizing the formation of cytoplasmic granules containing TDP-43. We transfected HeLa cells with the TDP-43 D169G HA encoding plasmid. Remarkably, GQDs-Cl and Cl-GQDs-OH efficiently prevented and disassembled stress-induced TDP-43 D169G-positive SGs (Fig. 3k–n). Overall, our results highlight the robust effectiveness of halogen atom-doped GQDs in preventing and disassembling stress-induced TDP-43 LLPS, both in NBs and cytoplasmic granules.

### Halogen atom-doped GQDs inhibit TDP-43 LC fibrillation

**Fig. 4:**
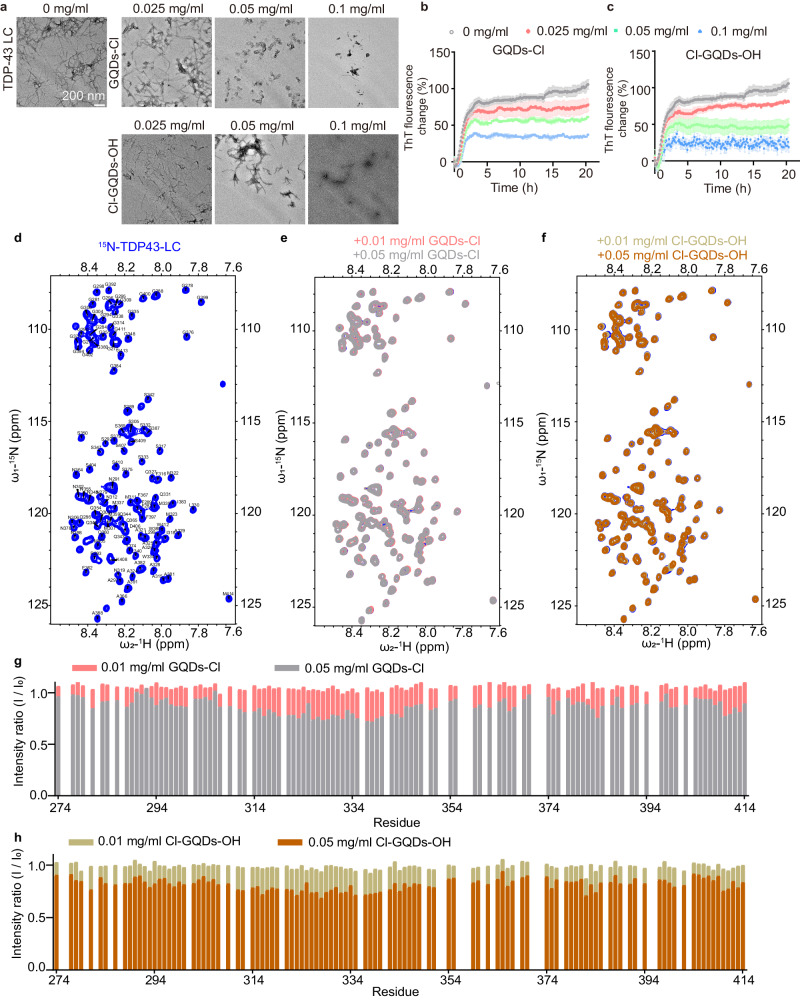
Halogen atom-doped GQDs interact with and inhibit TDP-43 LC fibrillation. **a** TEM images of the ThT samples in the presence and absence of GQDs at 20 h. Scale bar, 200 nm. The imaging was independently repeated 3 times with similar observations. **b**, **c** ThT fluorescence assay of 20 μM TDP-43 LC monomers fibrillation in the presence of different concentrations of GQDs. Data correspond to the mean ± SEM, *n* = 3. **d** 2D ^1^H-^15^N HSQC spectrum of TDP-43 LC. Peaks are labeled with the one-letter amino acid code and sequence number in TDP-43. **e**, **f** Overlay of the 2D ^1^H-^15^N HSQC spectra of 20 μM ^15^N-TDP-43 LC in the presence of GQDs-Cl or Cl-GQDs-OH. **g**, **h** Changes in the intensity ratio of residues in the 2D ^1^H-^15^N HSQC spectra of 20 μM ^15^N-labeled TDP-43 LC in the presence of GQDs. The *x*-axis represents the sequence number of residues in TDP-43 LC. Source data are provided as a Source Data file.

TDP-43 features an N-terminal domain with dimerization propensity, two RRM domains with β-α-β motifs, and an LCD domain, mediating liquid-to-solid phase transitions and pathological fibril formation^33,34^. BLI assays were used to quantify the binding affinity between halogen atom-doped GQDs and TDP-43 protein domains. The results demonstrate that halogen atom-doped GQDs interact with various domains of TDP-43, including the NTD, RRM, and LC domain (Supplementary Fig. 11a–f). The *K*_D_ of GQDs-Cl and Cl-GQDs-OH to the TDP-43 NTD was measured as 19.60 μM and 10.78 μM, respectively. These GQDs also exhibit a similar binding affinity to the TDP-43 RRM domain, with *K*_D_ values of 18.96 μM and 13.33 μM. Notably, for the TDP-43 LC domain, BLI data revealed that halogen atom-doped GQDs bind with *K*_D_ values of 1.50 μM and 1.35 μM, suggesting the strongest binding affinity among the three domains, potentially attributable to its flexible structure. TDP-43 is known to form pathological amyloid fibrils, a process primarily driven by its N-terminal LC domain^33,34^. This process is associated with NDs such as ALS and FTD. ThT kinetic assay and TEM data suggest that GQDs-Cl and Cl-GQDs-OH strongly inhibited the amyloid aggregation of TDP-43 LC in a dose-dependent manner (Fig. 4a–c). To investigate the interaction between GQDs-Cl or Cl-GQDs-OH with TDP-43 LC domain at the residue level, we employed nuclear magnetic resonance (NMR) spectroscopy^35^. By titrating both GQDs into ^15^N-labeled TDP-43 LC, we observed a gradual decrease in signal intensities in the HSQC spectra (Fig. 4d–h). These results indicate direct binding between TDP-43 LC and halogen atom-doped GQDs. Notably, this decrease in intensity was not localized to a specific region of TDP-43 LC but was observed to uniformly across its entire LC domain (Fig. 4d–h). This observation suggests that the interaction between TDP-43 LC and GQDs is likely to be weak and transient, and non-specific to any particular region or residue of the TDP-43 LC domain.

### Halogen atom-doped GQDs can inhibit aggregation of TDP-43 and decrease pTDP-43 (S409/410) level

**Fig. 5:**
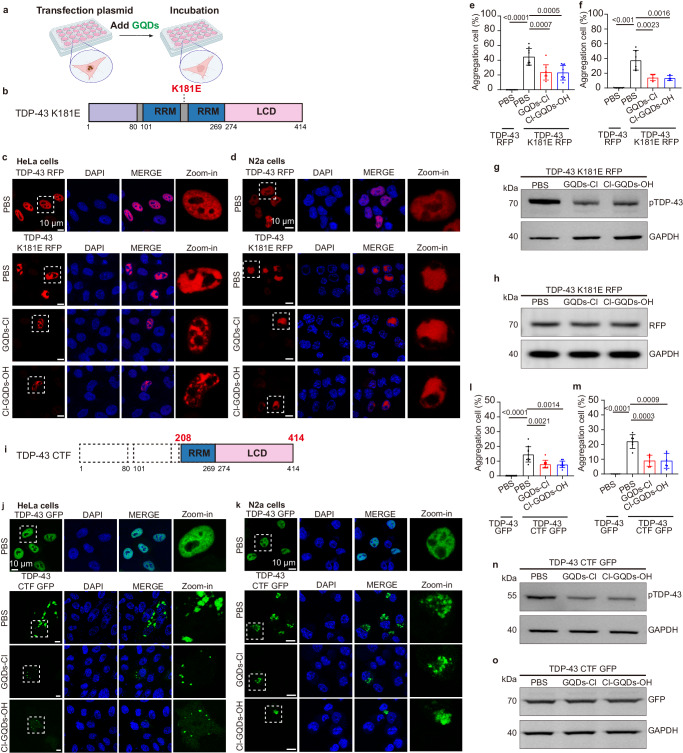
GQDs inhibit the aggregation of TDP-43 and reduce the pTDP-43 (S409/410) levels in the nucleus and cytoplasm. **a** Schematic diagram of the assay for inhibition of GQDs on TDP-43 aggregation in cells. Created with BioRender.com. **b** Domain architecture of TDP-43 K181E mutant. **c**, **d** Fluorescence images of HeLa cells and N2a cells transfected with TDP-43 K181E RFP. Cells were treated with PBS or 0.02 μg/μl GQDs for 16 h. Scale bar, 10 μm. The imaging was independently repeated 3 times with similar observations. **e** Quantitative analysis of the number of cells with aggregates for images (**c**). Data correspond to the mean ± SD, *n* = 9, two-tailed unpaired *t*-test. **f** Quantitative analysis of the number of cells with aggregates for images (**d**). Data correspond to the mean ± SD, *n* = 6, two-tailed unpaired *t*-test. **g**, **h** Western blot for the expression of pTDP-43 (S409/410) or RFP in the transfected 293T cells. GAPDH serves as a loading control. The imaging was independently repeated 3 times with similar observations. **i** Domain architecture of TDP-43 CTF mutant. **j**, **k** Fluorescence images of HeLa cells or N2a cells transfected with TDP-43 CTF GFP. Cells were treated with PBS or 0.02 μg/μl GQDs for 16 h. Scale bar, 10 μm. The imaging was independently repeated 3 times with similar observations. **l** Quantitative analysis of the number of aggregates for image (**j**). Data correspond to the mean ± SD, *n* = 10, two-tailed unpaired *t*-test. **m** Quantitative analysis of the number of aggregates for image (**k**). Data correspond to the mean ± SD, *n* = 6, two-tailed unpaired *t*-test. **n**, **o** Western blot for the expression of pTDP-43 (S409/410) or GFP in the transfected 293T cells. GAPDH serves as a loading control. The imaging was independently repeated 3 times with similar observations. Source data are provided as a Source Data file.

To explore the potential impact of GQDs on TDP-43 nuclear aggregates, we transfected the TDP-43 K181E plasmid into cells. Remarkably, incubation with either GQDs-Cl or Cl-GQDs-OH markedly reduced the number of aggregated cells in both HeLa cells and N2a cells, indicating the potential of GQDs-Cl or Cl-GQDs-OH for inhibiting TDP-43 nucleus aggregation (Fig. 5a–f). Furthermore, both GQDs-Cl and Cl-GQDs-OH were observed to increase the number of cells with dispersed protein (Supplementary Fig. 12a–c). pTDP-43 at S409/410 is a disease-specific marker of TDP-43 aggregates^24^. Next, we performed a WB analysis to examine the expression and pTDP-43 (S409/410) levels of TDP-43 K181E in the presence of GQDs. Intriguingly, while the expression level of the TDP-43 K181E plasmid was unaffected by GQDs (Fig. 5h), we noticed a discernible decrease in pTDP-43 (S409/410), suggesting a modulatory role for GQDs-Cl and Cl-GQDs-OH in regulating pTDP-43 (S409/410) (Fig. 5g).

Additionally, after transfecting cells with the TDP-43 CTF plasmid, we treated them with either GQDs-Cl or Cl-GQDs-OH. This treatment significantly reduced the number of cells with cytoplasmic aggregates while increasing those exhibiting cytoplasmic dispersion, as observed with both types of GQDs (Fig. 5i–m and Supplementary Fig. 13a–c). WB analysis confirmed that GQDs did not impact the expression level of the TDP-43 CTF plasmid, but resulted in a decrease in its pTDP-43 (S409/410) level (Fig. 5n, o). In summary, these findings underscore the ability of GQDs-Cl or Cl-GQDs-OH to efficiently inhibit the fibrillation and diminish disease-associated pTDP-43 (S409/410) in both the nucleus and cytoplasm.

## Discussion

The study focused on TDP-43, a protein implicated in NDs such as ALS and FTD, which forms both intranuclear and cytoplasmic TDP-43 condensates^11,36^. Previous studies mainly focused on the pathologies associated with cytoplasmic mislocalization and aggregation of TDP-43^9,37–39^. Whereas, recent findings have also observed nuclear aggregation of TDP-43 in affected brain regions of FTD and AD patients^11–14^, highlighting the pathological relevance of TDP-43 condensation in nucleus. Thus, understanding the mechanisms and regulation of phase separation and aggregate clearance of TDP-43 protein within the nucleus is vital for devising potential therapeutic strategies^10^. This study addressed the challenge of limited nuclear access of GQDs by designing and synthesizing halogen atom-doped GQDs to enhance nuclear permeability. The modified GQDs effectively penetrated the nuclear membrane, creating a promising avenue for controlling nuclear protein phase separation and aggregation. The results showed that GQDs-Cl and Cl-GQDs-OH could regulate the assembly of TDP-43 NBs and SGs under conditions of oxidative stress and hyperosmotic stimulation. Importantly, GQDs-Cl and Cl-GQDs-OH were found to suppress both the aggregation and pTDP-43 (S409/410) level within the nucleus and cytoplasm. The observations from this study offer initial insights into the possible therapeutic use of halogen atom-doped GQDs for influencing nuclear protein phase separation and aggregation. Of note, in addition to nuclear TDP-43 condensation, our designed GQDs also exhibits potential activity in affecting the cytoplasmic TDP-43 condensates.

Previous work showed that TDP-43 formed dynamic NBs in the nucleus in response to stress, playing an essential protective role in the cellular stress response^15,16^. The transiently formed TDP-43 NBs can be disassembled during recovery after stress. While, under chronic stress, TDP-43 NBs could be further solidified and loss capability for dynamic disassembly, which is believed to play a pathological role in disease. Thus, the properties and roles of TDP-43 condensates vary significantly, serving protective functions in normal stress responses while potentially becoming detrimental in disease states. We showed that the designed GQDs can inhibit the formation of both the dynamic condensate and solidified condensates of TDP-43 in nucleus (Figs. 3c–n and 5c–o). Thus, for potential therapeutic application, it will be important to apply GQDs to the cells with solidified TDP-43 condensates and aggregates rather than to those with dynamic TDP-43 NBs, in order to diminish the potential side-effect GQDs in disrupting the functional TDP-43 NBs in normal cells. Moreover, development of next generate GQDs which could specifically target the pathological TDP-43 condensates will be of importance.

In light of the finding that dysregulated LLPS can facilitate the formation of deleterious protein aggregates, there has been great interest in identifying chemical molecules that might modulate the LLPS of NDs-related protein^40^. Previous efforts have targeted small molecules like mitoxantrone, lipoamide, and lipoic acid, which have proven effective in disassembling SGs and inhibiting disease-associated proteins’ recruitment into these granules^41^. In the evolving landscape of nanotechnology, GQDs stand out as a revolutionary innovation. These zero-dimensional nanomaterials are recognized for their exceptional electronic, optical, and chemical properties^42^. GQDs are synthesized using both top-down methods, which involve segmenting larger graphene sheets, and bottom-up approaches, employing benzene derivatives or carbonization processes^43,44^. This flexibility in synthesis allows for the precise control of GQDs’ characteristics, including size, edge functionalization, surface features, activity, biocompatibility, and permeability through the blood-brain barrie^17,45–47^. Such control has been pivotal in optimizing their properties for specific applications, including bioimaging, drug delivery, photodynamic therapy, and nanozymes catalysis^48,49^.Given these attributes, GQDs could potentially contribute to the development of chaperone-like nanomaterials for NDs intervention and diagnosis^50,51^, though further studies are necessary to fully ascertain their effectiveness and applicability.

Finally, several limitations of applying GQDs in cells for modulating protein condensation must be considered. First, the complexity of MLOs in cells, containing a variety of different proteins and nucleic acids, creates challenges. The GQDs’ broad interaction with many different intracellular biomacromolecules necessitates further optimization of GQDs with specific client-recognition capability^52,53^. Moreover, due to the complexities associated with the absorption, metabolism, excretion, and immunogenicity of GQDs, it remains uncertain whether the in vitro and cellular effects of GQDs observed in this study can be replicated in the in vivo models of TDP-43-related NDs. Nevertheless, our research offers vital insights into GQDs’ potential as modulators of TDP-43 condensation in the context of ND treatment.

## Methods

### Materials

1,5-diaminonaphthalene was purchased from Aladdin. Reagent Co., ltd. (China). Ethanol, trichloromethane and catechol were purchased from Shanghai Titan Technology Co. Ltd. All chemical reagents were directly used.

### Preparation of GQDs

GQDs-NH_2_ and GQDs-OH were prepared using a conventional procedure, which was described in detail in our previous study^20^. To synthesize GQDs-OH, 0.1 g of 1,3,6-trinitropyrene was uniformly dispersed in 10 ml of deionized water through ultrasonication for 5 min. Subsequently, 1 ml of a 1 M NaOH solution was incorporated into the dispersed solution. This mixture was then transferred into a 25 ml poly(tetrafluoroethylene) (Teflon)-lined autoclave and subjected to a temperature of 180 °C for a duration of 12 h. Upon cooling to ambient temperature, the resultant product, which contained water-soluble GQDs, was filtered using a 0.22 μm microporous membrane to separate the insoluble carbonaceous material. For the synthesis of GQDs-NH_2_, the procedure was mirrored with the exception of substituting the NaOH solution with 1 ml of ammonia, maintaining all other conditions identical. The GQDs-Cl* and GQDs-Cl were synthesized by solvothermal treatment. Briefly, 0.1 g 1,5-diaminonaphthalene was dissolved in 10 ml ethanol/trichloromethane solvent (7:3 or 9:1 in volume), the solution was then transferred to a Teflon-lined autoclave (25 ml) and heated at 180 °C for 12 h. The Cl-GQDs-OH was prepared by using precursor of the same quality with ethanol/trichloromethane (8.5:1.5 in volume) under the same solvent thermal conditions. The crude product solution was added 0.025 g of catechol for secondary reaction. After cooled to room temperature, all the GQDs colloids were dialyzed in a 3500 Da dialysis for 1 week and dried at 80 °C for structural characterization. The GQDs were directly used without purification for characterization of optical properties.

### GQDs characterization

The optical properties of GQDs were characterized by Horiba Duetta. The time-resolved PL spectra were characterized with a Horiba FluoroMax. Raman spectra were performed by an Anton Paar Cora 5001 with 532 nm. FT-IR spectra were obtained by using a Thermo Scientific iS50. XPS spectra were assessed utilizing a Thermo ESCALAB 250Xi spectrometer. AFM images was recorded with ScanAsyst air mode using Nanoscope V Multimode 8 (Bruker).

### Protein expression and purification

TDP-43 full length was cloned into the pET9d vector with a TEV protease cleavable MBP-His tag at the C-terminus. We expressed the plasmid encoding TDP-43-MBP in BL21(DE3) PlySs *E. coli* cells (TransGen Biotech, China). Protein expression was instigated using 1 mM isopropyl β-D-1-thiogalactopyranoside (IPTG, Sigma-Aldrich, USA) at 16 °C for a duration of 16 h. Then cells were harvested and treated with a lysis buffer (comprising 50 Tris-HCl, pH 7.5, 1 M NaCl, 2 mM DTT, 10% glycerol, 1 mM EDTA, and 2 mM PMSF). Post-lysis, we separated the proteins via centrifugation and loaded them onto an MBPSep Dextrin 6FF chromatography column. The elution process was then carried out with a buffer (50 mM Tris-HCl, pH 7.5, 1 M NaCl, 2 mM DTT, 10% glycerol, and 10 mM maltose). For further purification, we employed gel filtration chromatography (Superdex 200 16/300; GE Healthcare, UK) balanced with a storage buffer (50 mM Tris-HCl, pH 7.5, 300 mM NaCl, and 2 mM DTT). The final purified protein was concentrated and flash-frozen using liquid nitrogen before being stored in aliquots at −80 °C until further use.

TDP-43 LC was cloned into the pET28a vector with a 6-His tag at the N-terminus. For the purification of the TDP-43 LC construct, TDP-43 LC was overexpressed in BL21(DE3) E. coli cells (TransGen Biotech, CD601-02) by adding 1 mM IPTG and incubating at 37 °C for 12 h. The cells were then harvested and lysed in a buffer containing 50 mM Tris-HCl, pH 8.0, and 100 mM NaCl. Cell pellets were collected by centrifugation (30,000 × g, 4 °C, 1 h) and subsequently resuspended in a denaturing buffer containing 50 mM Tris-HCl, pH 8.0, 6 M guanidine hydrochloride with sonication. The resuspended protein was filtered and then loaded onto a Ni column (GE Healthcare, USA). The protein was eluted using a denaturing elution buffer containing 50 mM Tris, pH 8.0, 6 M guanidine hydrochloride and 100 mM imidazole, and was further concentrated into over 30 mg/ml proteins, flash-frozen and stored at −80 °C. Before experiments, the protein was desalted using a desalting column (GE Healthcare, USA) into a buffer containing 20 mM MES, pH 6.0. The protein was not concentrated after desalination and ThT experiment was carried out immediately.

TDP-43 NTD and RRM was cloned into the pET28a vector with a 6-His tag at the N-terminus. TDP-43 NTD and TDP-43 RRM was overexpressed in BL21(DE3) *E. coli* cells with 0.5 mM IPTG at 16 °C for 16 h. Cells were harvested and lysed in 50 mM Tris-HCl, pH 7.5, 500 mM NaCl, 2 mM β-mercaptoethanol and 2 mM PMSF. After removing cell pellets by centrifugation, protein was loaded onto His Trap HP column and then eluted with 50 mM Tris, pH 7.5, 500 mM NaCl, 500 mM imidazole. Eluted protein was purified over the gel filtration chromatography (Superdex 75 16/600; GE Healthcare) equilibrated with storage buffer (50 mM Tris, pH 7.5, 300 mM NaCl, 2 mM DTT).

### Fluorescent labeling of the proteins

TDP-43 MBP proteins were desalted into a reaction buffer (50 mM Tris, pH 7.5, 500 mM NaCl, and 4 mM TCEP (Invitrogen, T2556)) to remove DTT from the storage buffer. Subsequently, the proteins were incubated with a fluorescent dye at a 5-fold molar ratio, specifically using Alexa 488 C5-malemide (Invitrogen, A10254) and Alexa 555 C2-malemide (Invitrogen, A20346). This labeling reaction was carried out for more than 1 h at room temperature. Afterward, the labeled proteins were purified using Superdex 200 10/300 columns (GE Healthcare, USA). For subsequent LLPS assays and confocal imaging, unlabeled proteins were mixed with labeled proteins at a molar ratio of 49:1.

### ThT fluorescence kinetic assay

The ThT fluorescence kinetic assay, owing to its specific affinity for β-sheet amyloid fibril structures, was employed to observe the progression of amyloid fibrils. Measurements were taken from a 50 µl sample housed in a NUNC 384-well plate. The fluorescence signal was captured using a microplate reader operating at excitation and emission wavelengths of 440 nm and 480 nm, respectively. The TDP-43 LC ThT samples were incubated under conditions of 37 °C, with a 20 µM concentration of TDP-43 LC, in a buffer solution (20 mM MES, pH 6.0). Each condition was reproduced in four separate experimental replicates for consistency.

### Negative-staining TEM

To study the morphology of the fibrils, we employed negative staining TEM techniques. Initially, we hydrophilized the copper mesh using a glow discharge instrument to treat the carbon support film. Subsequently, we incubated 5 µl of the sample on the hydrophilized side of the carbon-coated grids for 45 s. The sample was then rinsed with dd H_2_O and subjected to two successive rounds of uranyl acetate staining, each lasting 45 s. The prepared sample was ultimately loaded onto a Tecnai G2 Spirit transmission electron microscope operating at a voltage of 120 kV.

### Cell culture and transfection

We sourced cells from the Shanghai Cell Bank of the Chinese Academy of Sciences: HeLa cells (SCSP-504); 293T cells (SCSP-5035); N2a cells (SCSP-502). All cell types were cultured in Dulbecco’s Modified Eagle Medium (DMEM) supplemented with 10% fetal bovine serum (FBS, VISTECH) and 1% penicillin, under a controlled environment of 37 °C with 5% CO_2_. Cells were seeded 18 to 24 h prior to transfection to achieve a monolayer density of ~70–80%. Plasmids were introduced into cells using PolyJetTM reagent (SignaGen Laboratories) and after 6 h, we replaced the transfection medium with fresh DMEM. Unless otherwise specified, all cells were allowed a 24-h post-transfection period before undergoing any further drug treatments or assessments.

### Preparation and immunofluorescence staining assay

We fixed the cells with 4% paraformaldehyde for 30 min at room temperature, followed by three washes with PBS. The cells were then permeabilized using 0.5% Triton X-100 in PBS for 30 min and subsequently blocked with a PBS buffer containing 3% goat serum and 0.1% Triton X-100 (PBST) for 1 h at room temperature. We incubated the cells with the primary antibody overnight at 4 °C. After three washes with PBST, the cells were treated with secondary antibodies for 1–2 h at room temperature. For mounting, we used ProLongTM Gold Antifade Mountant with DAPI (Thermo Fisher Cat #P36935). Fluorescent images were captured using a SP8 Leica microscope equipped with a DMI8 camera and further analyzed using Leica AF Lite software. The following antibodies were utilized: primary antibody: anti-HA (AB_1549585, Cat#3724, Cell Signaling Technology), anti-GFP (AB_2619674, Cat#M20004, Abmart) anti-pS409/410-TDP-43 (Cat#66318-1-lg, Proteintech), anti- anti-G3BP (AB_398438, 611127, BD Biosciences), anti-RFP (ab62341, abcam), anti-Lamin B (ab133741, abcam), anti-Caspase 3(Cat#9661, Cell Signaling), The dilution ratio for above antibodies was 1:1000. GAPDH (Cat#AT0002, Engibody), The dilution ratio for these antibodies was 1:10,000. HRP conjugated secondary antibodies: goat anti-mouse (Sigma, A4416), goat anti-rabbit (Sigma, A9169), The dilution ratio for these antibodies was 1:10,000. Fluorescent secondary antibodies: goat anti-rabbit-Alexa Flour 488 (Life Technologies, A11034), goat anti-rabbit-Alexa Flour 568 (Life Technologies, A11011), goat anti-mouse-Alexa Flour 568 (Life Technologies, A11031), goat anti-mouse Flour 488 (Life Technologies, A10680), The dilution ratio for these antibodies was 1:1000. The antibodies are well validated for the indicated use by the manufacturer available on their websites.

### BLI

The binding kinetics were assessed using a ForteBio Octet RED96 system (Pall ForteBio LLC). A total volume of 200 μl either sample or buffer (50 mM Tris, pH 7.5, 150 mM NaCl) was dispensed into a 96-well black flat-bottom plate. The plate was incubated at 37 °C with orbital shaking set to 1000 rpm. The biosensors were first incubated with GQDs to eliminate the non-specifically binding. Proteins were immobilized onto the Ni-NTA biosensors (Sartorius, 185101) at a concentration of 20 μg/ml. Different concentrations of GQDs were further incubated with biosensors during the association step, followed by a disassociation step. The resulting curves were adjusted using a blank reference and analyzed using the ForteBio Data Analysis software (version 9.0).

### Cell viability assay

Post-transfection, HeLa cells were seeded in 96-well plates at a density of 10,000 cells/well and cultivated in 100 μl of culture medium. Following 24 h of transfection, cells were treated with 20 μg ml^−1^ and 100 μg ml^−1^ of GQDs at different time points. Subsequently, cell viability was assessed using the Cell Counting Kit-8 (CCK-8) (Dojindo) in accordance with the manufacturer’s protocol. In brief, we added 10 μl of the CCK-8 solution to each well, followed by an hour of incubation at 37 °C. Ultimately, we measured the absorbance at 450 nm using a Synergy2 microplate reader (BioTek Instruments).

### NMR spectroscopy

The NMR experiments were conducted using a Bruker Avance 900 MHz spectrometer equipped with a cryogenically cooled probe. The backbone assignment was accomplished according to previous publications^35^. We prepared each NMR sample to a total volume of 500 μl, incorporating 20 μM of ^15^N-TDP-43 LC in 20 mM MES (pH 6.0), 10% D_2_O, either with or without GQDs at concentrations of 0.01 μg/μl or 0.05 μg/μl for titration experiments. We collected the 2D ^1^H-^15^N HSQC spectra using the Bruker standard pulse sequence (hsqcetfpf3gpsi) with 16 scans at 298 K. For both ^1^H (16 ppm) and ^15^N (19 ppm) dimensions, the data matrix was set to 2048 × 160 complex points. The experimental setup, including NMR buffer compositions and data collection parameters, was in substantial accordance with those described in prior publications^35^, ensuring the accuracy of the transferred chemical shifts in our study. Signal intensity changes were calculated by the ratio *I*/*I*_0_, where *I* denote the intensity in the HSQC spectrum of TDP-43 LC with GQDs, and *I*_0_ indicates the intensity in the HSQC spectrum of TDP-43 LC alone. Data acquisition was performed using Topspin (3.5pl5), while NMR data analysis was performed using NMRViewJ^54^ (9.2.0) and SPARKY^55^ (3.113).

### Western blotting

Cultured cells’ total protein was extracted using a 4% extraction buffer (100 mM Tris pH 6.8, 4% SDS, 1% mercaptoethanol, 40% glycerol, and 0.04% bromophenol blue) that included a protease inhibitor cocktail and phosphatase inhibitor (Beyotime Biotechnology, China). Subsequently, the protein samples were subjected to a 10-min boiling period at 100 °C, followed by separation via 10% or 12.5% sodium dodecyl sulfate-polyacrylamide gel electrophoresis (SDS-PAGE). The separated proteins were transferred to a nitrocellulose filter membrane and blocked using 5% skimmed milk in Tris-buffered saline with Tween 20 (TBST). Following this, the membranes were incubated with primary antibodies at 4 °C overnight. After three wash cycles with TBST solution, the bound primary antibodies were detected with a 1:2000 dilution of HRP-conjugated secondary antibody, and protein signals were visualized using the BeyoECL Star analysis reagent (Beyotime Biotechnology, China).

### Statistical analysis

GraphPad Prism and Microsoft Excel software were used for statistical analysis. All experiments were repeated independently for more than three times. The statistical significance in this study is determined by two tailed *t-*test at *p < 0.05, **p < 0.01, and ***p < 0.001, ns: no significant.

### Reporting summary

Further information on research design is available in the Nature Portfolio Reporting Summary linked to this article.

### Supplementary information

Supplementary Information

Reporting Summary

Peer Review File

### Source data

Source Data

## Data Availability

All data supporting the findings of this study are available within the article and in the Supplementary Information. Source data are provided with this paper.
