# Supplementary material for: Halogen doped graphene quantum dots modulate TDP-43 phase separation and aggregation in the nucleus
Source: Nat Commun. 2024 Apr 6;15:2980. doi: 10.1038/s41467-024-47167-x (PMC10998863; doi:10.1038/s41467-024-47167-x)

Supplementary Information

Halogen Doped Graphene Quantum Dots Modulate TDP-43 Phase Separation and Aggregation in the Nucleus

Hong Zhang[1], Huazhang Guo[2], Danni Li[1], Yiling Zhang[1], Shengnan Zhang[3], Wenyan Kang[4,5], Cong Liu[3], Weidong Le[6,7], Liang Wang[2*], Dan Li[8,9,10*], Bin Dai[1*]

[1] School of Electronic Information and Electrical Engineering, Shanghai Jiao Tong University, Shanghai 200240, China

[2]Institute of Nanochemistry and Nanobiology, School of Environmental and Chemical Engineering, Shanghai University, 99 Shangda Road, Baoshan District, Shanghai 200444, P. R. China

[3]Interdisciplinary Research Center on Biology and Chemistry, Shanghai Institute of Organic Chemistry, Chinese Academy of Sciences, Shanghai 201210, China

[4]Department of Neurology and Institute of Neurology, Ruijin Hospital, Shanghai Jiao Tong University School of Medicine, Shanghai 200025, China

[5]Department of Neurology, Ruijin Hainan Hospital, Shanghai Jiao Tong University School of Medicine (Boao Research Hospital), Hainan, 571434, China

[6]Shanghai University of Medicine & Health Sciences Affiliated Zhoupu Hospital, Shanghai 201318, China

[7]Center for Clinical and Translational Medicine, Shanghai University of Medicine and Health Sciences, Shanghai 201318, China

[8]Key Laboratory for the Genetics of Developmental and Neuropsychiatric Disorders (Ministry of Education), Bio-X Institutes, Shanghai Jiao Tong University, Shanghai 200030, China

[9]Bio-X-Renji Hospital Research Center, Renji Hospital, School of Medicine, Shanghai Jiao Tong University, Shanghai 200240, China

[10]Zhangjiang Institute for Advanced Study, Shanghai Jiao Tong University, Shanghai 200240, China

[*]To whom correspondence should be addressed. Email: wangl@shu.edu.cn, lidan2017@sjtu.edu.cn, daibin@sjtu.edu.cn.

-Supplementary Fig. 1-13
-Source Data

a

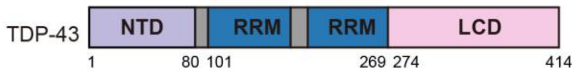

b

1-50	MSEYIRVTED	ENDEPIEIPS	EDDGTVLLST	VTAQFPGACG	LRYRNPVSQC
51-100	MRGVRLVEGI	LHAPDAGWGN	LVYVVNYPKD	NKRKMDETDA	SSAVKVKRAV
101-151	QKTSDLIVLG	LPWKTTEQDL	KEYFSTFGEV	LMVQVKKDLK	TGHSKGFGFV
151-200	RFTEYETQVK	VMSQRHMIDG	RWCDCKLPNS	KQSQDEPLRS	RKVFVGRCTE
201-250	DMTEDELREF	FSQYGDVMDV	FIPKPFRAFA	FVTFADDQIA	QSLCGEDLII
251-300	KGISVHISNA	EPKHNSNRQL	ERSGRFGGNP	GGFGNQGGFG	NSRGGGAGLG
301-351	NNQGSNMGGG	MNFGAFSINP	AMMAAAQAAL	QSSWGMMGML	ASQQNQSGPS
351-400	GNNQNQGNMQ	REPNQAFGSG	NNSYSGSNSG	AAIGWGSASN	AGSGSGFNGG
401-414	FGSSMDSKSS	GWGM			

Supplementary Fig. 1: Amino acid sequence of full length TDP-43 protein. a Domain architecture of TDP-43. **b** TDP-43 NTD (1-80) marked with blue, TDP-43 RRMs (101-269) marked with green and TDP-43 LC (274-414) marked with red.

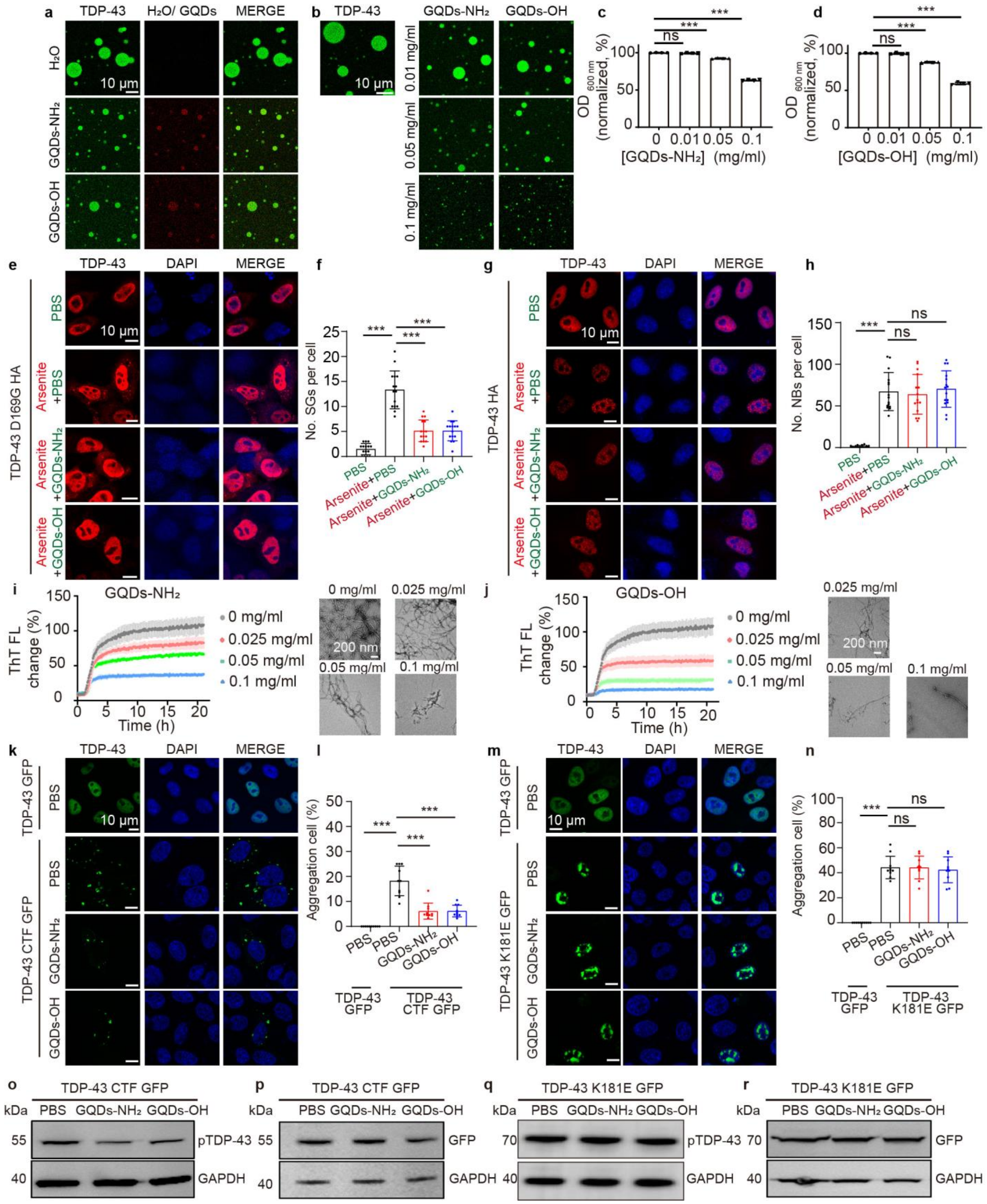

Supplementary Fig. 2: GQDs-NH$_2$ and GQDs-OH inhibit the TDP-43 phase separation *in vitro*. **a** Fluorescent images of the 40 μM TDP-43-MBP in the presence and absence of 0.01 μg/μL GQDs. Buffer condition: 50 mM Tris, pH 7.5, 150 mM NaCl, 10% Dextran 70. Scale bar, 10 μm. **b** Representative fluorescence images of 40 μM TDP-43 MBP undergoing phase separation in the presence of different concentrations of GQDs-NH$_2$ or GQDs-OH. Scale bar, 10 μm. **c-d** Turbidity measurement of the TDP-43 MBP phase separation in the presence of GQDs-NH$_2$ or GQDs-OH. Mean ± SD, n = 4, two-tailed unpaired *t*-test. ***P < 0.001; ns: not significant. **e,g** Representative confocal images of HeLa cells transfected with TDP-43 D169G HA or TDP-43 HA. Cells were treated with 250 μM sodium arsenite for 1h and then treated with 0.1 μg/μL GQDs or PBS for 1 h. Scale bar, 10 μm. **f,h** Quantitative analysis of number of SGs in cytoplasm per cell for image e or the

number of NBs in nucleus per cell for image g. Mean ± SD, n = 15, two-tailed unpaired *t*-test. ***P < 0.001; ns: not significant. **i-j** ThT fluorescence assay of 20 μM TDP-43 LC monomers fibrillation in the presence of different concentrations of GQDs-NH_2 or GQDs-OH. Mean ± SD, n = 3. TEM images of the ThT samples in the presence and absence of GQDs at 20 h are shown right. Scale bar, 200 nm. **k,m** Fluorescence images of HeLa cells transfected with TDP-43 CTF GFP or TDP-43 K181E GFP. Cells were treated with PBS or 0.02 μg/μL GQDs-NH_2 or GQDs-OH for 16 h. Scale bar, 10 μm. **l,n** Quantitative analysis of the number of aggregation cells for image k and m. Data correspond to the mean ± SD, n =10, two-tailed unpaired *t*-test, ***P < 0.001; ns: not significant. **o-r** Western blot for the expression of pTDP-43 or GFP in the transfected 293T cells. GAPDH serves as a loading control. The imaging for a-b, e, g, i, j, k, m, o-r was independently repeated 3 times with similar observations. Source data are provided as a Source Data file.

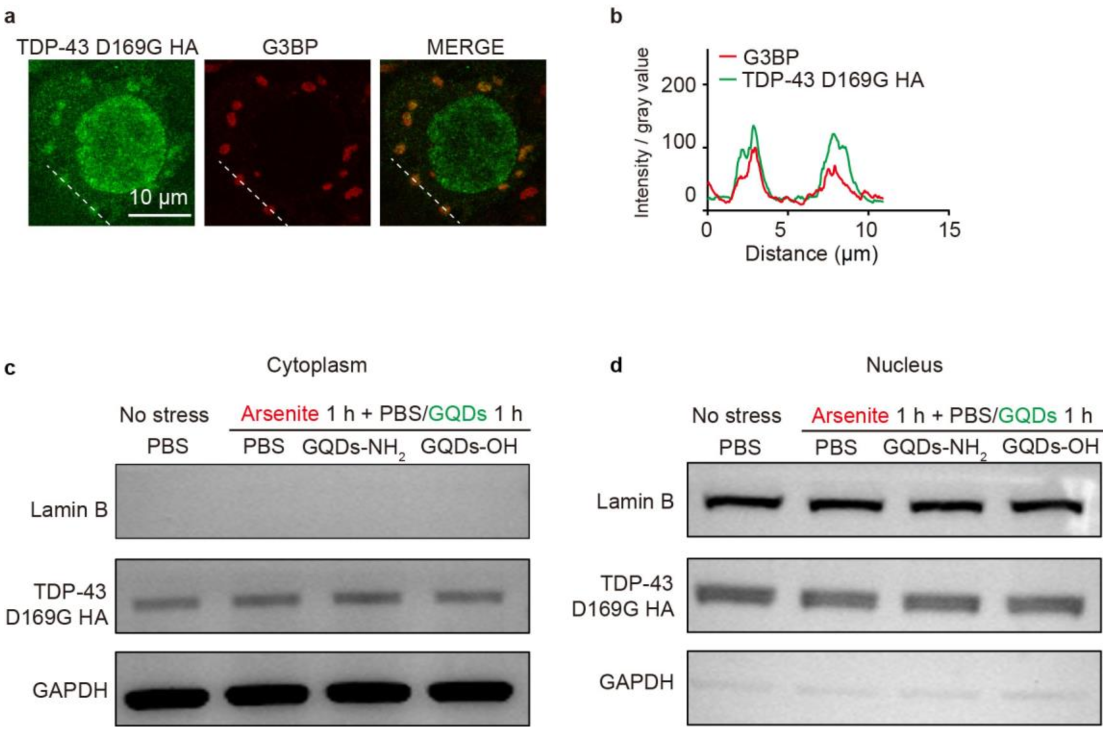

Supplementary Fig. 3: TDP-43 D169G HA is co-localized with the SGs in HeLa cells. a Representative confocal images of HeLa cells transfected with plasmid TDP-43 D169G HA. Cells were treated with 250 µM sodium arsenite for 1 h. Scale bar, 10 µm. The imaging was independently repeated 3 times with similar observations. **b** The co-localization of D169G HA with G3BP was evaluated by the intensity profile. **c-d** Western blot analysis of TDP-43 D169G HA expression in cytoplasmic and nuclear fractions of transfected HeLa cells. Cells were treated with 250 µM sodium arsenite for 1h, followed by 0.1 µg/µL GQDs-NH_2 or GQDs-OH or PBS (control) for 1 h. GAPDH serves as a cytoplasm loading control, and Lamin B serves as a nuclear loading control. The imaging was independently repeated 3 times with similar observations. Source data are provided as a Source Data file.

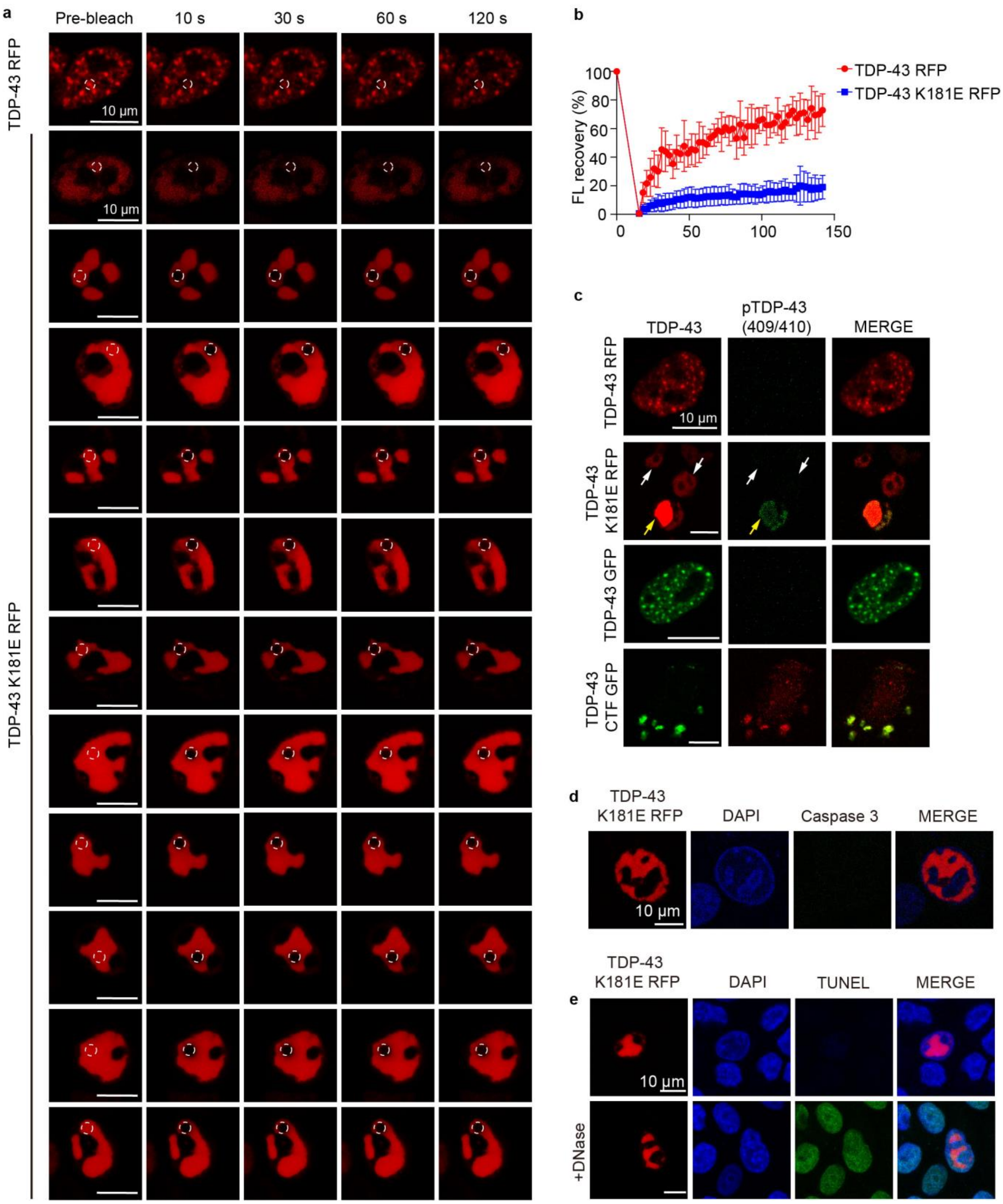

Supplementary Fig. 4: Aggregation of TDP-43 K181E in nucleus. a Representative fluorescence microscopy images showing TDP-43 RFP nuclear bodies and TDP-43 K181E RFP nuclear aggregates, with regions (dashed circles) subjected to photobleaching in the FRAP assay. The imaging was independently repeated 3 times with similar observations. **b** Fluorescence intensity recovery curves are displayed, TDP-43 RFP n=5, TDP-43 K181E RFP n=11. **c** Immunofluorescence images of 293T cells transfected with the respective plasmids, stained for phosphorylated TDP-43 (S409/410). Irregularly shaped aggregates co-localizing with pTDP-43 (S409/410) antibodies (highlighted by yellow arrows), in contrast to cells with dispersed protein, which lack phosphorylation at S409/410 (denoted by write arrows). The imaging was independently repeated 3 times with similar observations. **d** Images of HeLa cells expressing TDP-43 K181E RFP, immunostained for Caspase-3 to assess apoptosis. The imaging was independently repeated 3 times with similar observations. **e** TUNEL assay

on HeLa cells expressing TDP-43 K181E RFP, with DNase treatment serving as a positive control. The imaging was independently repeated 3 times with similar observations. Source data are provided as a Source Data file.

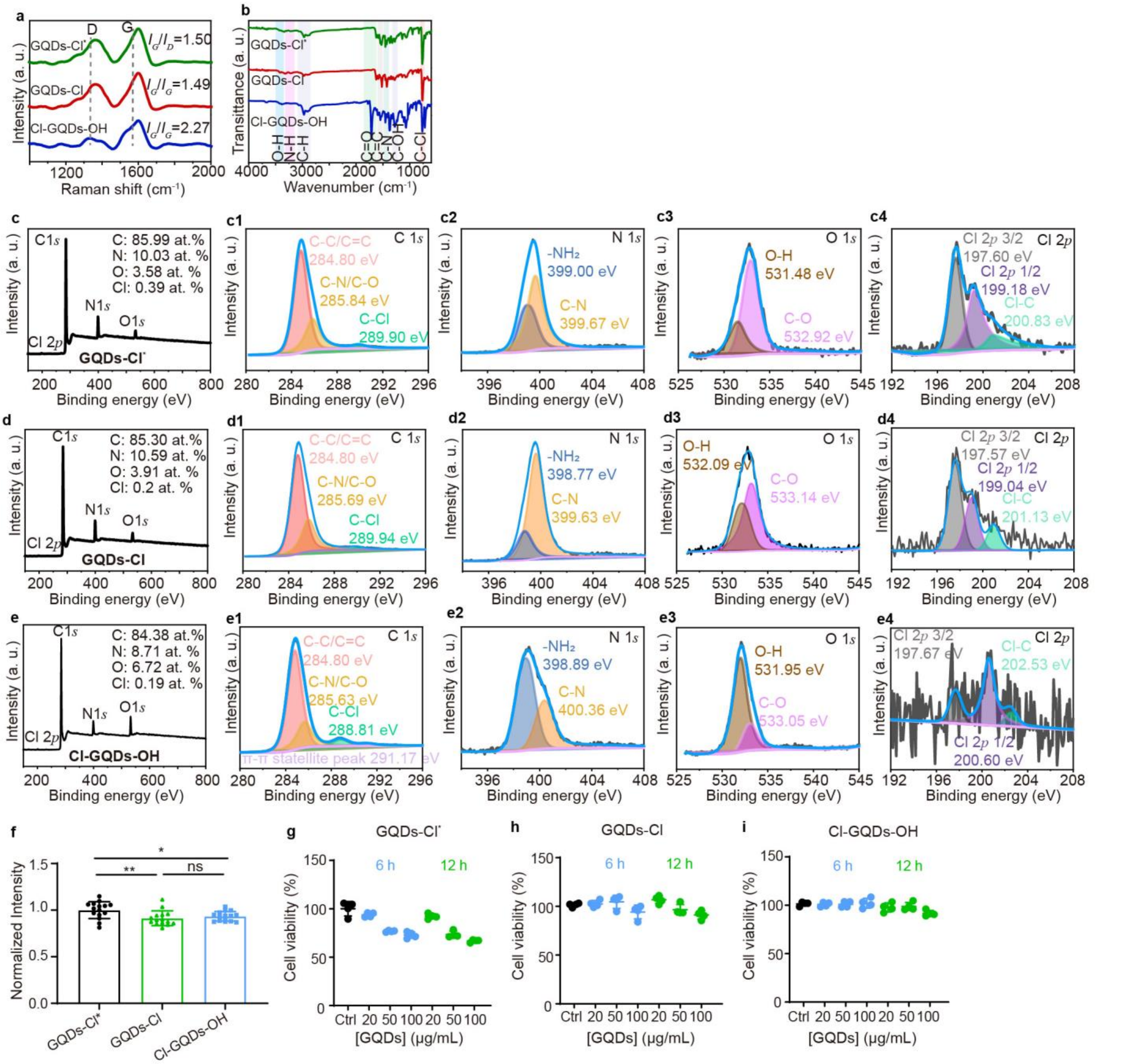

Supplementary Fig. 5: Structural characterization and cytotoxicity analysis of halogen atom-doped GQDs. **a** Raman spectrum of the halogen atom-doped GQDs. **b** FT-IR spectrum of the halogen atom-doped GQDs. **c-e** XPS spectra of the three types of halogen atom-doped GQDs and high-resolution spectra for C1s, N1s, O1s, and Cl2p. **f** Normalized fluorescence intensity of HeLa cells (normalized to its own fluorescence). Data correspond to the mean ± SD, n =15, two-tailed unpaired *t*-test, **P < 0.01; *p<0.05; ns: not significant. **g-i** Cell viability assay results for the three types of halogen atom-doped GQDs at the imaging dose and incubation time. Data correspond to the mean ± SD, n =4. Source data are provided as a Source Data file.

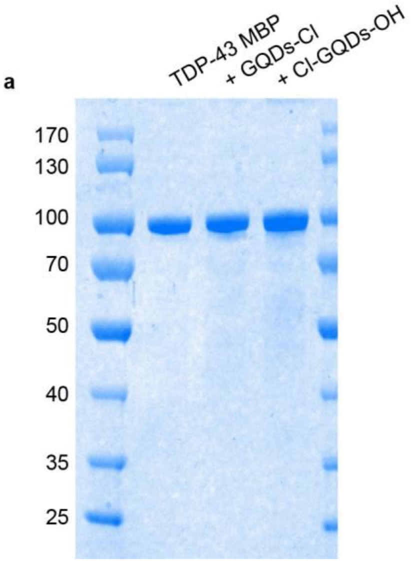

Supplementary Fig. 6: SDS-PAGE of TDP-43-MBP protein. a SDS-PAGE of TDP-43 MBP at a concentration of 20 μM in a buffer solution (50 mM Tris, pH 7.5, 150 mM NaCl, 10% Dextran 70), both with and without the addition of 0.01 μg/μL GQDs-Cl or Cl-GQDs-OH. The imaging was independently repeated 3 times with similar observations. Source data are provided as a Source Data file.

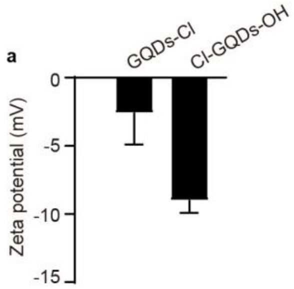

Supplementary Fig. 7: Characterization of zeta potentials of GQDs. a Zeta potential of GQDs-Cl and Cl-GQDs-OH in 50mM Tris, 150 mM NaCl. Data correspond to the mean ± SD, n=3. Source data are provided as a Source Data file.

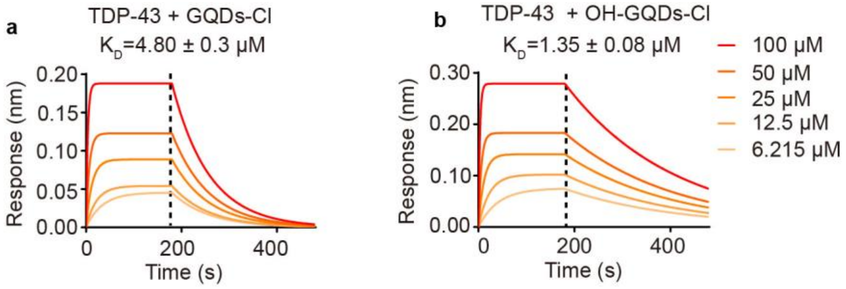

Supplementary Fig. 8. Binding affinity of halogen atom-doped GQDs to TDP-43 protein. a-b Kinetic binding curves of TDP-43 with a concentration gradient of GQDs-Cl or Cl-GQDs-OH. The association and dissociation profiles are divided by a vertical black dotted line. Source data are provided as a Source Data file.

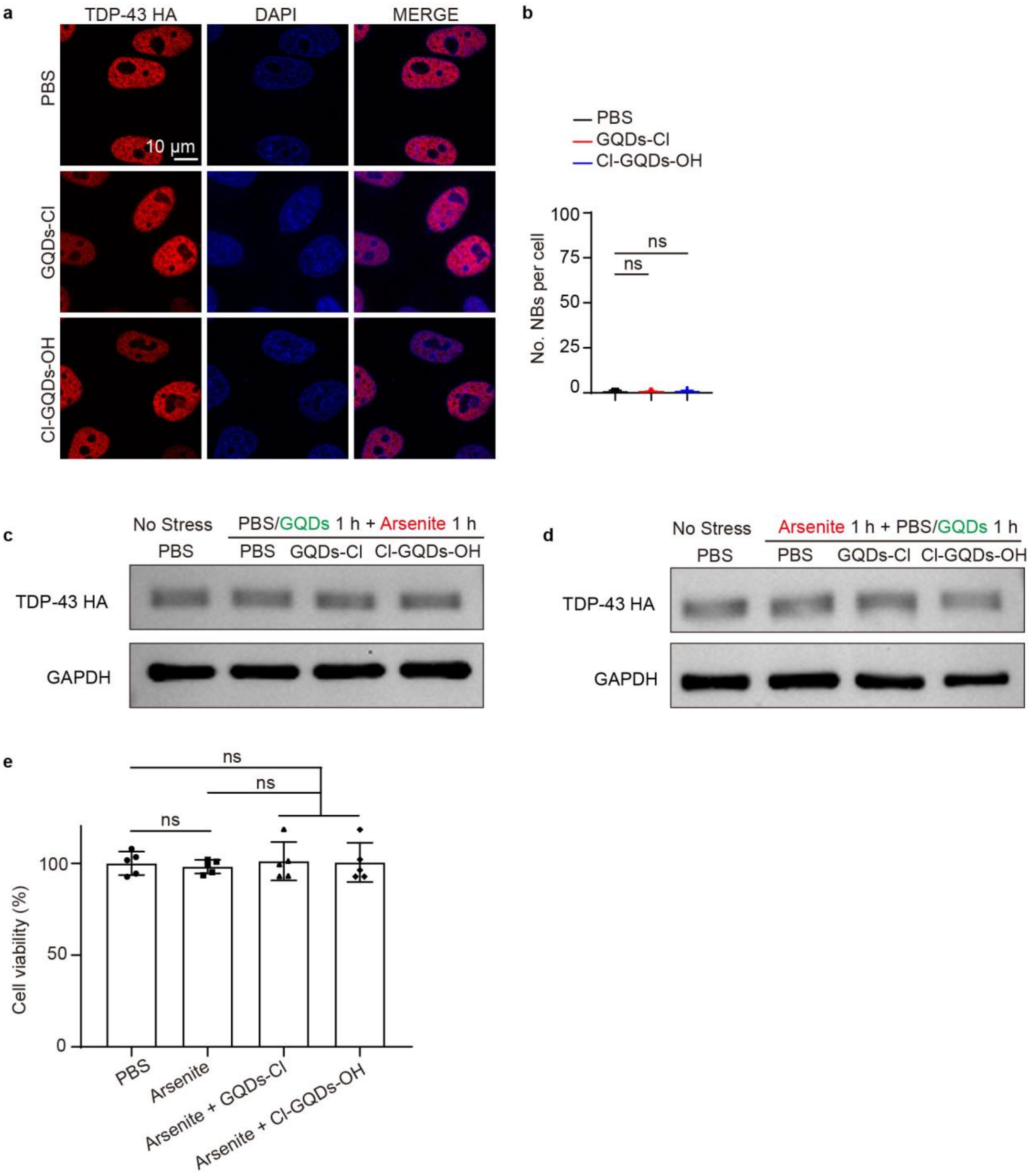

Supplementary Fig. 9: Halogen atom-doped GQDs alone cannot induce the NBs assembly do not affect the expression of TDP-43. a Representative confocal images of HeLa cells transfected with TDP-43 HA plasmid and then incubated with 0.1 µg/µl GQDs for 2 h. Scale bar, 10 µm. The imaging was independently repeated 3 times with similar observations. **b** Data correspond to the mean ± SD, n = 15, two-tailed unpaired *t*-test. ns: not significant. **c-d** Western blot analysis of TDP-43 HA transfected HeLa cells treated with sodium arsenite and GQDs-NH_2 or GQDs-OH. GAPDH serves as a control. The imaging was independently repeated 3 times with similar observations. **e** Cell viability was assessed in cells treated with 250 µM sodium arsenite for 1 hour, followed by 0.1 µg/µL halogen atom-doped GQDs or PBS (control) for 1 h. Cell viability analysis measured by CCK8 assay. Data correspond to the mean ± SD, n = 5, two-tailed unpaired *t*-test, ns: not significant. Source data are provided as a Source Data file.

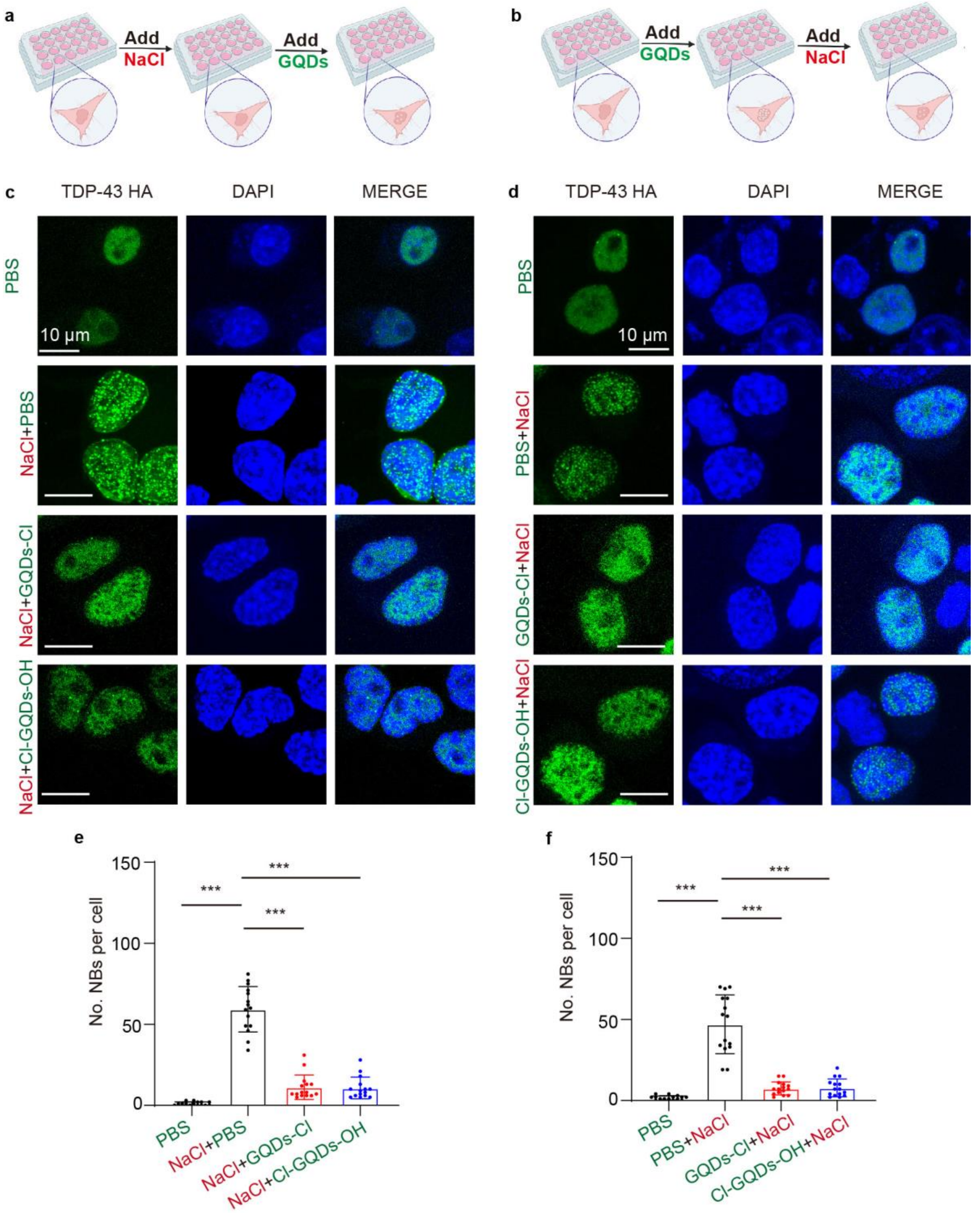

Supplementary Fig. 10: GQDs disassemble preformed TDP-43 nucleus granules as well as prevent their formation in N2a cells. a Schematic diagram of TDP-43 granules disassembly assay. Created with BioRender.com. **b** Schematic diagram of the assay for GQDs inhibition LLPS of TDP-43. Created with BioRender.com. **c** Representative confocal images of N2a cells transfected with TDP-43 HA. Cells were treated with 300 mM NaCl for 1h and then treated with 0.1 μg/μL both types of GQDs or PBS (control) for 1 h. Scale bar, 10 μm. The imaging was independently repeated 3 times with similar observations. **d** Representative confocal images of N2a cells transfected with TDP-43 HA. Cells were treated with 0.1 μg/μL GQDs or PBS (control) for 1h and then treated with for 300 mM NaCl 1 h. Scale bar: 10 μm. The imaging was independently repeated 3 times with similar observations. **e-f** Quantitative analysis of the number of NBs in nucleus per cell for image c and d. Data correspond to the mean ± SD, n = 15, two-tailed unpaired *t*-test, ***P < 0.001. Source data are provided as a Source Data file.

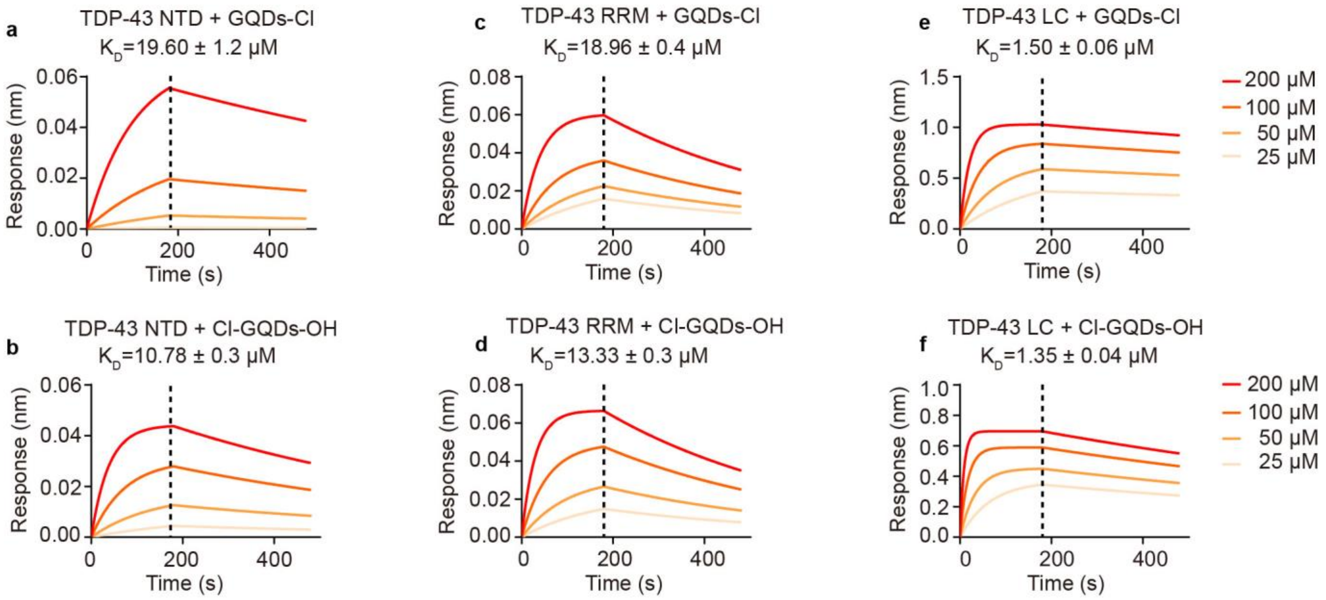

Supplementary Fig. 11: Binding affinity of GQDs to various TDP-43 domains. a-f Kinetic binding curves of TDP-43 NTD, RRM, LC domain with a concentration gradient of GQDs-Cl or Cl-GQDs-OH. The association and dissociation profiles are divided by a vertical black dotted line.

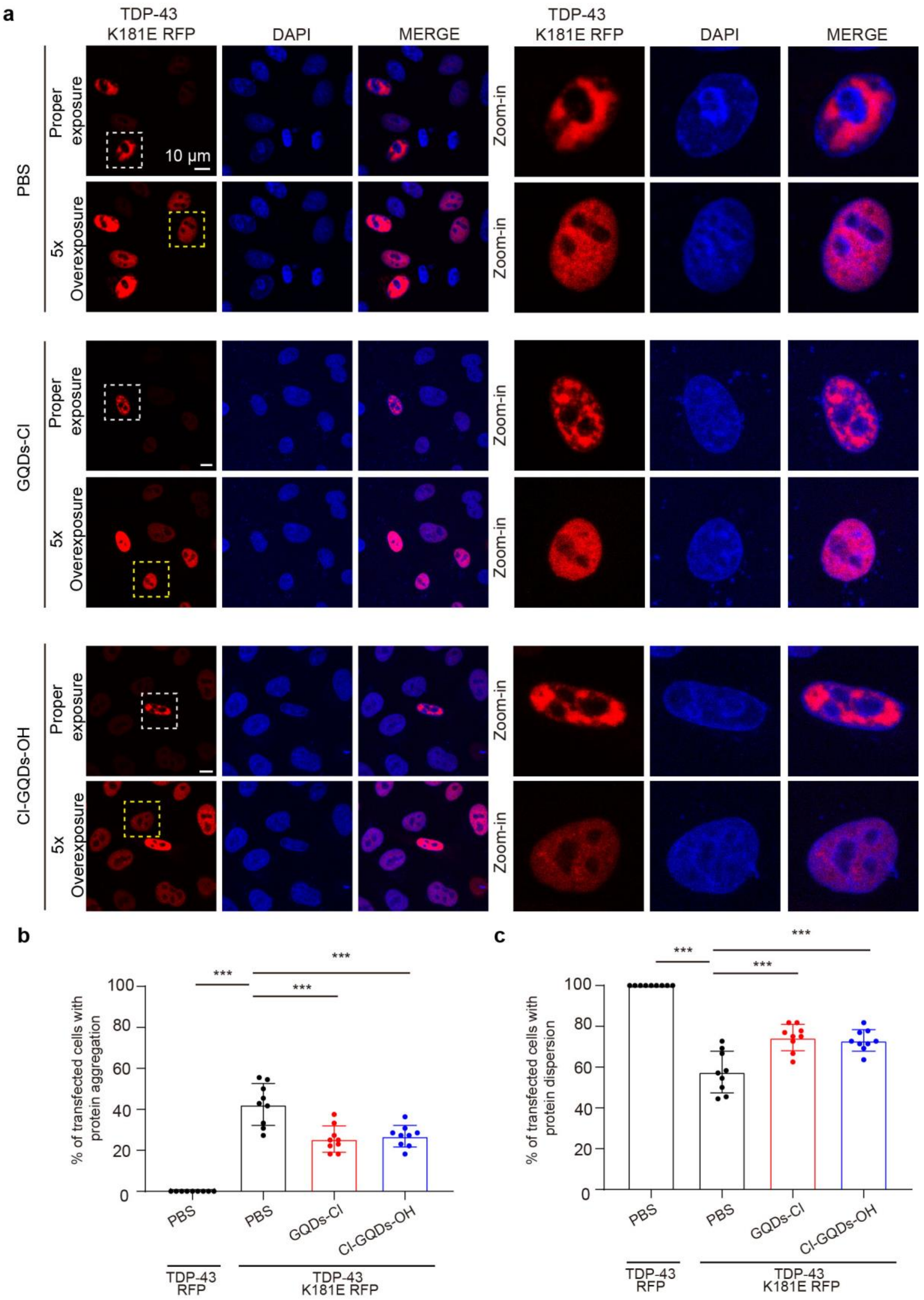

Supplementary Fig. 12: Impact of GQDs-Cl and Cl-GQDs-OH on protein aggregation and dispersion in the nucleus of HeLa cells. a Fluorescence microscopy images of HeLa cells transfected with TDP-43 K181E RFP and treated with either PBS or 0.02 μg/μL GQDs for 16 hours, under proper and 5x exposure conditions. The white dotted box indicates cells with protein aggregation, while the yellow dotted box shows cells with protein dispersion. Scale bar represents 10 μm. The imaging was independently repeated 3 times with similar observations. **b** Quantitative analysis of the number of cells displaying aggregates for images a. Data correspond to the mean ± SD, n =9, two-tailed unpaired *t*-test, ***P < 0.001. **c** Quantitative analysis of the number of transfected cells exhibiting protein dispersion for image a. Data correspond to the mean ± SD, n =9, two-tailed unpaired *t*-test, ***P < 0.001. Source data are provided as a Source Data file.

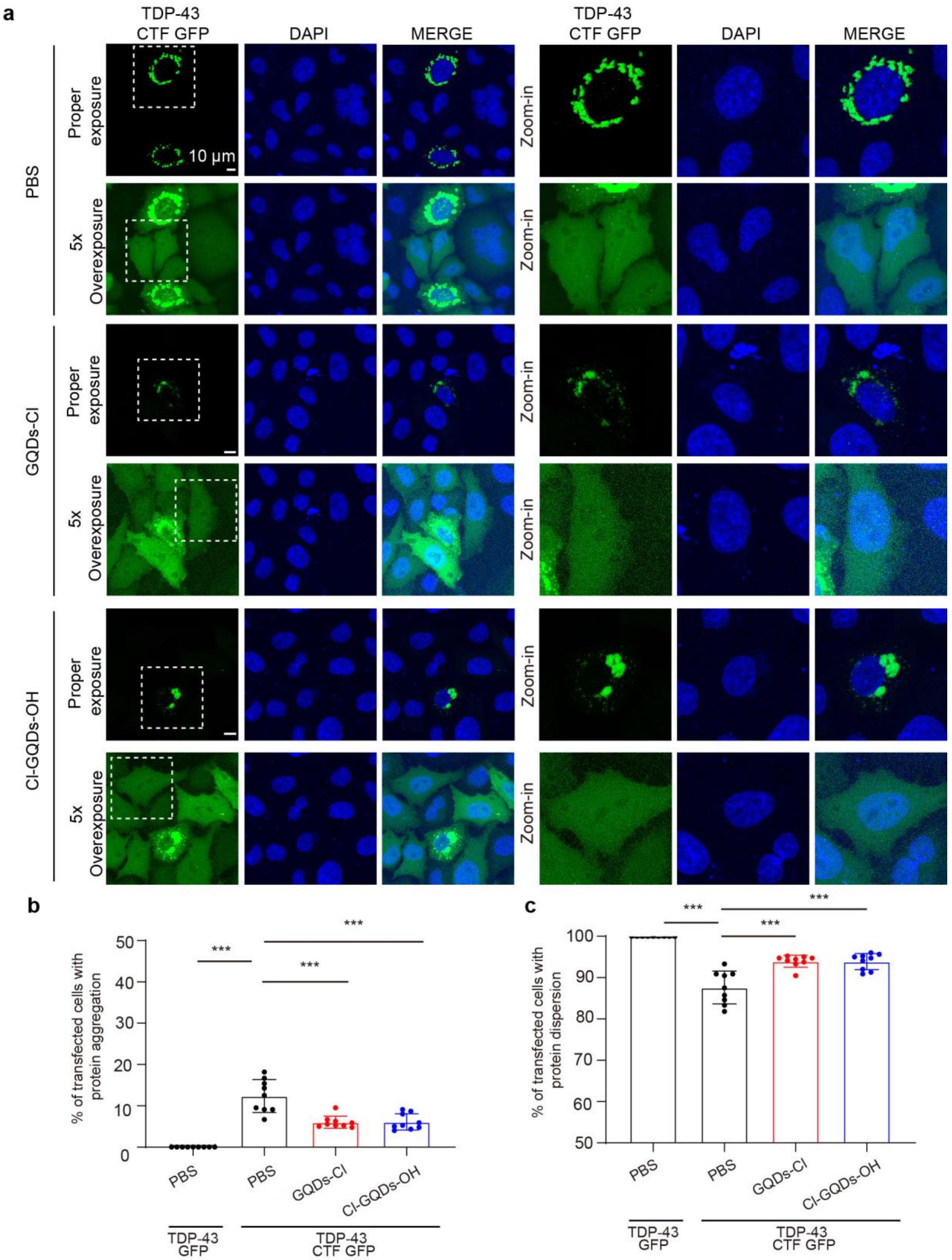

Supplementary Fig. 13: Impact of GQDs-Cl and Cl-GQDs-OH on protein aggregation and dispersion in the cytoplasm of HeLa cells. a Fluorescence microscopy images of HeLa cells transfected with TDP-43 CTF GFP and treated with either PBS or 0.02 μg/μL GQDs for 16 hours, under proper and 5x exposure conditions. Scale bar represents 10 μm. The imaging was independently repeated 3 times with similar observations. **b** Quantitative analysis of the number of cells displaying aggregates for images a. Data correspond to the mean ± SD, n =9, two-tailed unpaired *t*-test, ***P < 0.001. **c** Quantitative analysis of the number of transfected cells exhibiting protein dispersion for image a. Data correspond to the mean ± SD, n =9, two-tailed unpaired *t*-test, ***P < 0.001. Source data are provided as a Source Data file.

Source Data: Supplementary Fig. 2o

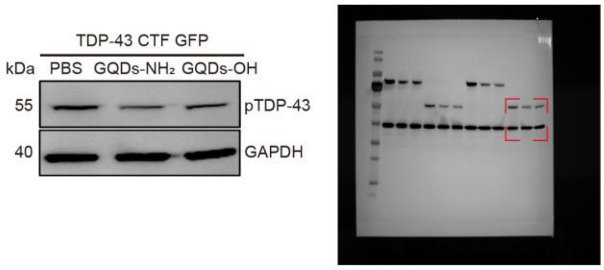

Source Data: Supplementary Fig. 2p

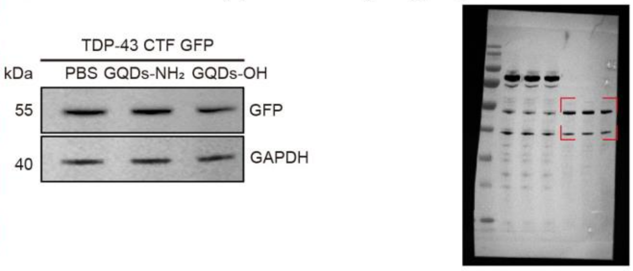

Source Data: Supplementary Fig. 2q

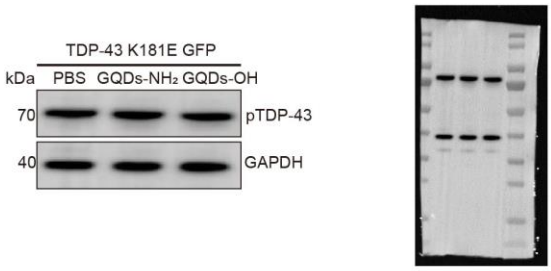

Source Data: Supplementary Fig. 2r

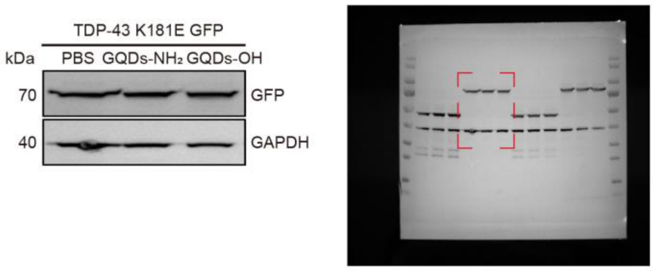

Source Data: Supplementary Fig. 6a

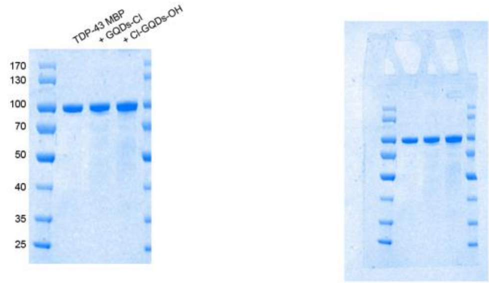

Source Data: Supplementary Fig. 9c

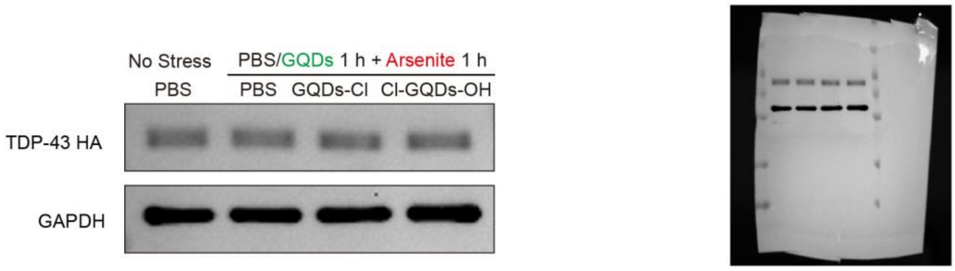

Source Data: Supplementary Fig. 9d

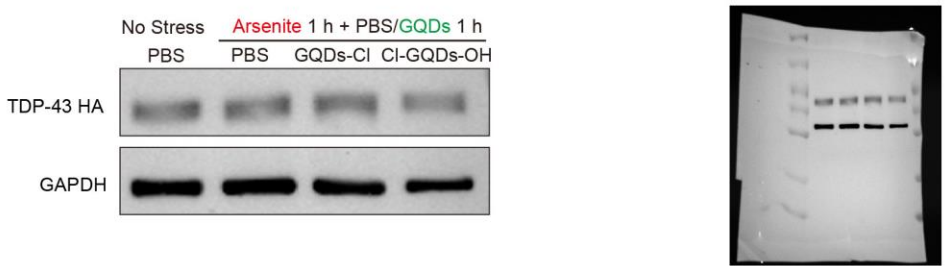

Supplement: Supplementary file 1 — Supplementary Information [file 41467_2024_47167_MOESM1_ESM.pdf]
