# Supplementary material for: Halogen doped graphene quantum dots modulate TDP-43 phase separation and aggregation in the nucleus
Source: Nat Commun. 2024 Apr 6;15:2980. doi: 10.1038/s41467-024-47167-x (PMC10998863; doi:10.1038/s41467-024-47167-x)
Supplement: Supplementary file 2 — Reporting Summary [file 41467_2024_47167_MOESM2_ESM.pdf]

nature portfolio

Corresponding author(s): Bin Dai, Dan Li, Liang Wang

Last updated by author(s): Mar 3, 2024

Reporting Summary

Nature Portfolio wishes to improve the reproducibility of the work that we publish. This form provides structure for consistency and transparency in reporting. For further information on Nature Portfolio policies, see our Editorial Policies and the Editorial Policy Checklist.

Statistics

For all statistical analyses, confirm that the following items are present in the figure legend, table legend, main text, or Methods section.

n/a	Confirmed	
☐	☒	The exact sample size (*n*) for each experimental group/condition, given as a discrete number and unit of measurement
☐	☒	A statement on whether measurements were taken from distinct samples or whether the same sample was measured repeatedly
☐	☒	The statistical test(s) used AND whether they are one- or two-sided<br>*Only common tests should be described solely by name; describe more complex techniques in the Methods section.*
☒	☐	A description of all covariates tested
☒	☐	A description of any assumptions or corrections, such as tests of normality and adjustment for multiple comparisons
☐	☒	A full description of the statistical parameters including central tendency (e.g. means) or other basic estimates (e.g. regression coefficient) AND variation (e.g. standard deviation) or associated estimates of uncertainty (e.g. confidence intervals)
☐	☒	For null hypothesis testing, the test statistic (e.g. *F*, *t*, *r*) with confidence intervals, effect sizes, degrees of freedom and *P* value noted<br>*Give P values as exact values whenever suitable.*
☒	☐	For Bayesian analysis, information on the choice of priors and Markov chain Monte Carlo settings
☒	☐	For hierarchical and complex designs, identification of the appropriate level for tests and full reporting of outcomes
☒	☐	Estimates of effect sizes (e.g. Cohen's *d*, Pearson's *r*), indicating how they were calculated

Our web collection on statistics for biologists contains articles on many of the points above.

Software and code

Policy information about availability of computer code

Data collection	LAS X (Leica) , Nanoscope V Multimode 8 (Bruker), Kaleido™ (EnSight-PerkinElmer), ForteBio Octet RED96 system (Pall ForteBio LLC), TEM imaging&analysis (120 kV), HORLBA EzSpec (1.3.0.63)
Data analysis	Image J (2.0.0), NanoScope Analysis (1.5), GraphPad Prism (8.0), Image Lab (3.0), Sparky (3.113), NMRViewJ (9.2.0), Origin (8.0), Data Analysis HT (Octet RED96, ForteBio, 9.0), TEM Users Interface (120 kV), Avantage 5.948.

For manuscripts utilizing custom algorithms or software that are central to the research but not yet described in published literature, software must be made available to editors and reviewers. We strongly encourage code deposition in a community repository (e.g. GitHub). See the Nature Portfolio guidelines for submitting code & software for further information.

Data

Policy information about availability of data

All manuscripts must include a data availability statement. This statement should provide the following information, where applicable:

- Accession codes, unique identifiers, or web links for publicly available datasets
- A description of any restrictions on data availability
- For clinical datasets or third party data, please ensure that the statement adheres to our policy

All data supporting the findings of this study are available within the article and in the Supplementary Information. Source data are provided with this paper.

Research involving human participants, their data, or biological material

Policy information about studies with human participants or human data. See also policy information about sex, gender (identity/presentation), and sexual orientation and race, ethnicity and racism.

Reporting on sex and gender	n/a
Reporting on race, ethnicity, or other socially relevant groupings	n/a
Population characteristics	n/a
Recruitment	n/a
Ethics oversight	n/a

Note that full information on the approval of the study protocol must also be provided in the manuscript.

Field-specific reporting

Please select the one below that is the best fit for your research. If you are not sure, read the appropriate sections before making your selection.

[x] Life sciences [ ] Behavioural & social sciences [ ] Ecological, evolutionary & environmental sciences

For a reference copy of the document with all sections, see nature.com/documents/nr-reporting-summary-flat.pdf

Life sciences study design

All studies must disclose on these points even when the disclosure is negative.

Sample size	The experiments described in this study were performed with more than 3 samples for each group. No statistical methods were used to predetermine sample size. Sample sizes were determined based on previous related studies.
Data exclusions	None
Replication	At least three independent biological repeats were performed for the experiments. All attempts at replication were successful.
Randomization	No randomization has been performed. Purified protein and cells were used in this study and no animal or human studies were involved.
Blinding	No animal or human studies were involved; no blinding was used for data analysis.

Reporting for specific materials, systems and methods

We require information from authors about some types of materials, experimental systems and methods used in many studies. Here, indicate whether each material, system or method listed is relevant to your study. If you are not sure if a list item applies to your research, read the appropriate section before selecting a response.

Materials & experimental systems

n/a	Involved in the study	
[ ]	[x]	Antibodies
[ ]	[x]	Eukaryotic cell lines
[x]	[ ]	Palaeontology and archaeology
[x]	[ ]	Animals and other organisms
[x]	[ ]	Clinical data
[x]	[ ]	Dual use research of concern
[x]	[ ]	Plants

Methods

n/a	Involved in the study	
[x]	[ ]	ChIP-seq
[x]	[ ]	Flow cytometry
[x]	[ ]	MRI-based neuroimaging

Antibodies

Antibodies used	anti-HA (AB_1549585, Cat#3724, Cell Signaling Technology), anti-GFP(AB_2619674, Cat#M20004, Abmart) anti-pS409/410-TDP-43 (Cat#66318-1-lg, Proteintech), anti-GAPDH (Cat#AT0002, Engibody) anti-G3BP (AB_398438, 611127, BD Biosciences), anti-RFP (ab62341, abcam), anti-Lamin B(ab133741, abcam), anti-Caspase 3(Cat#9661, Cell Signaling). HRP conjugated secondary antibodies: goat anti-mouse (Sigma, A4416), goat anti-rabbit (Signma, A9169). Fluorescent secondary antibodies: goat anti-rabbit-Alexa Flour 488 (Life Technologies, A11034),goat anti-rabbit-Alexa Flour 568 (Life Technologies, A11011), goat anti-mouse-Alexa Flour 568 (Life Technologies, A11031), goat anti-mouse Flour 488 (Life Technologies, A10680).
Validation	cCell Signaling Technology in immunostaining of COS cells, immunoprecipitation of 293T cell extracts, and western blot analysis of extracts from HeLa cells . Anti-GFP antibody has been validated by plenty of publications: PMID:36577386, PMID:35247331, PMID:33296677, PMID:34283998, PMID:34706230. Anti-pS409/410-TDP-43 has been validated by Proteintech in western blot analysis of HeLa cells. Anti-GAPDH has been validated by Engibody in western blot analysis of mouse cell lines. Anti-G3BP has been validated by BD Biosciences in western blot analysis of aSW-13 cell lysate and immunofluorescence staining of A431 cells. Anti-RFP has been validated by abcam in western blot analysis of RFP protein. Anti-Lamin B has been validated by abcam in western blot analysis of HeLa cell lysate, immunofluorescent analysis of Ramos cells, immunohistochemistry analysis of human transitional cell carcinoma of the bladder tissue. Anti-Caspase-3 has been validated by Cell Signaling in western blot analysis of extract from HeLa, NIH-3T3 and C6 cells, immunofluorescent of HT29 cells.

Eukaryotic cell lines

Policy information about cell lines and Sex and Gender in Research

Cell line source(s)	HeLa cells (SCSP-504), 293T cells (SCSP-5035), and N2a cells (SCSP-502) were purchased from cell bank of the Chinese Academy of Science, Shanghai.
Authentication	Cells have been authenticated by STR method.
Mycoplasma contamination	The cell line is mycoplasma negative.
Commonly misidentified lines (See ICLAC register)	No commonly misidentified cell lines were used.

Plants

Seed stocks	n/a
Novel plant genotypes	n/a
Authentication	n/a
