# Supplementary material for: Halogen doped graphene quantum dots modulate TDP-43 phase separation and aggregation in the nucleus
Source: Nat Commun. 2024 Apr 6;15:2980. doi: 10.1038/s41467-024-47167-x (PMC10998863; doi:10.1038/s41467-024-47167-x)
Supplement: Supplementary file 3 — Peer Review File [file 41467_2024_47167_MOESM3_ESM.pdf]

nature portfolio

Peer Review File

Halogen Doped Graphene Quantum Dots Modulate TDP-43 Phase Separation and Aggregation in the Nucleus

Open Access This file is licensed under a Creative Commons Attribution 4.0 International License, which permits use, sharing, adaptation, distribution and reproduction in any medium or format, as long as you give appropriate credit to the original author(s) and the source, provide a link to the Creative Commons license, and indicate if changes were made. In the cases where the authors are anonymous, such as is the case for the reports of anonymous peer reviewers, author attribution should be to 'Anonymous Referee' followed by a clear attribution to the source work. The images or other third party material in this file are included in the article's Creative Commons license, unless indicated otherwise in a credit line to the material. If material is not included in the article's Creative Commons license and your intended use is not permitted by statutory regulation or exceeds the permitted use, you will need to obtain permission directly from the copyright holder. To view a copy of this license, visit http://creativecommons.org/licenses/by/4.0/.

Reviewers' Comments:

Reviewer #1:
Remarks to the Author:
I am competent to comment on the biology part of the manuscript only (not chemistry)
The ability of GQDs to modulate nuclear and cytoplasmic condensate formation by TDP-43 is interesting and these can be a useful tool for the community. However, the biology behind the dataset is unfortunately weak. The entire study is based on the premise that nuclear condensate formation by TDP-43 is toxic/pathological (with references selected accordingly, in a biased way). While this might be true for mutant TDP-43 assemblies such as K181E mutant, the likelihood of TDP-43 nuclear bodies being a part of normal stress response is at least as high as that of them being detrimental. These structures form transiently during stress and then disassemble during recovery, and in fact were shown to be protective in some systems (PMID 32649883). In fact, D169G mutant is deficient in their assembly which can indicate a loss of function effect. Similarly, the role of TDP-43 phosphorylation is ambiguous, a recent study found that this PTM solubilises TDP-43 assemblies which might be protective (PMID 35112738). What makes the authors so convinced that it is beneficial to disassemble nuclear condensates of TDP-43? Condensates of TDP-43 can be a double-edged sword in neurodegeneration – something that the study failed for acknowledge. Overall, GQDs reported here can be instrumental in probing the cellular role of TDP-43 nuclear bodies and this would be the most logical development of this work – unbiased (and a lot more extensive and technically sound) studies of the role of TDP-43 phase separation (including the effect of PTMs) in the nucleus and cytoplasm.
Technical comments
1. Quality of cell images is low therefore it is not always possible to appreciate the phenotypes described. In several cases, there is a mismatch between the claims in the text and the images, controls are missing and sometimes it is not clear what was quantified and how:
- Fig3 – non-arsenite treated controls are missing, so nuclear body assembly cannot be assessed. In panel h, PBS and GQD treated cells look same to me. Overall, the level of granulation after stress cannot be assessed without a control. How do authors know that stress has worked?
- Fig3c,d – GQD seem to downregulate TDP-43, is this really the case?
- Fig3 – No. granules per cell was quantified for both nuclear bodies and SGs, was analysis done in the nucleus and cytoplasm separately?
- Fig5c – top (control) panel, image is hugely overexposed, does it mean that the expression level is significantly higher? The resolution and magnification are quite low so it is impossible to tell whether these are nuclear bodies or apoptotic/pyknotic cells. What does "aggregation cell" readout mean?
- Fig5, why TDP-43 total levels not provided here and moved to supplementary? It is essential to have them side by side.
- FigS4 – no quantification.
2. Line 95: It should be specified what type of stress is being used and that 169 mutant does not form SGs in naïve cells. Does GQD also prevent cytoplasmic mislocalisation of TDP-43 169? This should be measured, to prove that it is condensation that is affected by GQDs rather than cytoplasmic levels of the protein.
3. Line 230: this is over-simplification. N-terminal domain of TDP-43 (NTD) is important for its physiological oligomerisation and functionality.
4. A toxicity readout for cells with decreased TB assembly would be required.

Reviewer #2:
Remarks to the Author:
In the manuscript, Dai et al. reported that the function of neurodegenerative diseases-related protein TDP-43 could be manipulated by functionalized graphene quantum dots (GQDs) during the LLPS process. Considering the intricacies of nuclear phase separation and aggregate elimination, an in-depth research of nucleus-localized TDP-43 still remains challenging. Herein, the halogen-doped GQDs showed superior nucleus-targeted property which could prevent the construction of both TDP-43 nuclear bodies and TDP-43-positive stress granules. What's more, GQDs also showed great potential in restraining both aggregation and phosphorylation levels within the nucleus and cytoplasm. From my perspective, this manuscript is interesting and shows potential for the

neurodegenerative diseases-related therapy. Thus, I recommend this manuscript for publication after minor revisions.

For brevity, the concerns are listed below:
1. The author showed that the introduction of chlorine atoms into GQDs increased nucleus uptake. What’s the mechanism of nucleus uptake progress promoted by chlorine-doped GQDs? Does the amount of chlorine atoms affect the uptake behavior of nucleus?

2. As an emerging bio-nanomaterial, GQDs have displayed promise in treating various diseases. the authors should give a comprehensive overview regarding the advances on design and applications of GQDs, close-related recent references in this field, such as Chem. Rev., 2019, 119, 4357-4412; Angew. Chem. Int. Ed. 2019, 58, 7087–7091; Angew. Chem. Int. Ed.,2015, 54, 7176-7180; Mater. Today, 2013, 16, 433-442 etc., should be considered to cite; this would be very helpful for understanding how to design and develop suitable GQDs for bioapplications.

3. It is convincing that GQDs modulate the LLPS through electrostatic and hydrophobic interactions. The comparation of binding affinity of GQDs with or without halogen should be provided. Besides, the zeta potential of different GQDs are also suggested to be supplied.

4. The author claimed that the interaction between TDP-43 LC and GQDs was likely to be weak and transient, and non-specific to any particular region or residue of the TDP-43 LC domain. Considering the intricate biological environment which may disrupt the weak interaction and the potential off-target toxicity to other proteins, how does the author consider this issue?

Reviewer #3:
Remarks to the Author:
It is a very convincing manuscript by Li, Bai and coworkers on the use of graphene quantum dots to manipulate the formation of TDP-43 aggregation in cellulo.
TDP-43 is a crucial protein implied in numerous processes associated with RNA interaction and it has been proposed that the protein play a key role in several neurodegenerative disorders through its aggregation into amyloid fibrils and phase separation. The protein is very challenging, because unlike abeta, the sequence is much longer and contain several functional (and structurally folded) domains in its native state.

The authors present a long list of experimental results showing a significant effect of graphene quantum dots, especially the potential use of GQD in the nucleus, to manipulate the protein aggregation state. I recommend the manuscript for publication, however I have two main comments on the sample preparation and the use of the low complexity construct, as well as several minor comments (see below).

Main comment:

#1: Some clarification is required concerning the sample preparation of the TDP-43 protein:

. The authors use a non-native TDP-43 construct, that comprises the maltose binding protein fusion protein (MBP). It is unclear if and how MBP might affect the effect of graphene quantum dots. Can the authors comment on this possible MBP - GQD interaction ? Or provide any relevant literature showing that GQD is not affected by the presence of the MBP ?

. Is the MBP also fused with a 6-His tag ? It is unclear from the protocol.

. A "TDP-43 LC" construct is also mentioned in the material and method. Is this construct also attached to MBP ? with 6-His ?

. What is the exact amino-acid sequence of the LC construct ?

. The full-length TDP-43 sequence is known to rapidly be cleaved into smaller C-terminal

fragments. A SDS gel of the full-length construct would be needed to clarify if in vitro experiments are done on the full-length protein or on C-terminal fragments.

#2: About the ThT experiments on the LC construct: I'm surprised (in a positive way) by the results shown in Figure SI1, panel i and j. TDP-43 LC is known to spontaneously aggregate when concentrated. Here the maximal concentration used is 0.1 mg/ml. I'm surprised to see that TDP-43 LC does not aggregate for almost 1 hour at this concentration. Can the authors comment on that ? A more detailed description of the protocol on (i) the LC construct purification and aggregation and (ii) how and when the time t=0 was fixed in the ThT experiments would be useful.

Minor comments:

. In figure 1, panel A: not clear what means "more $CHCl_3$"

. A figure showing the TDP-43 sequence with the position of the different domains is useful. It is present in the manuscript, but only in Figure 5h. I propose to introduce it earlier in the figures.

. The inhibition of droplet formation (shown in Supporting Figure SI1) seems less efficient compared to what was reported by the same authors for FUS (in the publication: https://doi.org/10.1021/acsnano.3c00001, in figure 2), using the same concentration of 0.1mg/mL. Can the authors comment of this aspect ?

. Immobilization of TDP-43 for the BLI experiment is not well described.

. Please comment if the use of the fused MBP in the TDP-43 construct can also lead to artifacts during the BLI experiments.

. The authors show that the LC domain directly interacts with GQDs. Can they rule out that the folded N-terminal domain or the RNA-binding motifs do not directly interact with GQDs ?

Point-by-point response to the reviewers' comments:

We thank the reviewers for the valuable comments and suggestions to our work. We have performed additional experiments, added new data, figures and quantification, and carefully revised the text as the reviewers suggested. All comments are addressed point-by-point below:

*Reviewers' comments are in *Italic*. Our responses are in blue. All revisions are highlighted in red in the revised manuscripts.

Reviewer#1:

Comments:

I am competent to comment on the biology part of the manuscript only (not chemistry)

The ability of GQDs to modulate nuclear and cytoplasmic condensate formation by TDP-43 is interesting and these can be a useful tool for the community. However, the biology behind the dataset is unfortunately weak. The entire study is based on the premise that nuclear condensate formation by TDP-43 is toxic/pathological (with references selected accordingly, in a biased way). While this might be true for mutant TDP-43 assemblies such as K181E mutant, the likelihood of TDP-43 nuclear bodies being a part of normal stress response is at least as high as that of them being detrimental. These structures form transiently during stress and then disassemble during recovery, and in fact were shown to be protective in some systems (PMID 32649883). In fact, D169G mutant is deficient in their assembly which can indicate a loss of function effect. Similarly, the role of TDP-43 phosphorylation is ambiguous, a recent study found that this PTM solubilises TDP-43 assemblies which might be protective (PMID 35112738). What makes the authors so convinced that it is beneficial to disassemble nuclear condensates of TDP-43? Condensates of TDP-43 can be a double-edged sword in neurodegeneration – something that the study failed for acknowledge. Overall, GQDs reported here can be instrumental in probing the cellular role of TDP-43 nuclear bodies and this would be the most logical development of this work – unbiased (and a lot more extensive and technically sound) studies of the role of TDP-43 phase separation (including the effect of PTMs) in the nucleus and cytoplasm.

Response: We appreciate the reviewers so much for raising the insightful and constructive comments. We totally agree with the reviewer that studying GQDs' effect in regulating TDP-43 condensation in nucleus and cytoplasm is equally interesting and important. However, we want to humbly explain the reason we focus on regulation of TDP-43 condensation by GQDs in nucleus is because of the novelty of the GQDs part. Application of GQDs in modulating protein condensation in cytoplasm has been reported previously. While, sending GQDs into nucleus for regulating protein condensation has not been reported yet. Another reason we focus on the nucleus GQDs application is that accumulating studies found that TDP-43 protein inclusion was observed not only in cytoplasm but also in nucleus in

the diseased brains[1-4]. Moreover, TDP-43 nuclear bodies can turn into amyloid structures under prolonged stress[5]. A most recent study suggests that nuclear TDP-43 pathology may be an early event occurring prior to the clinical inflection point between pre-symptomatic and symptomatic states[6]. These data support the pathological relevance and importance of modulating TDP-43 nuclear bodies and aggregation[5, 7] in nucleus.

We agree with the reviewer that TDP-43 nuclear bodies could play an important role in the normal stress response as demonstrated in our previous work (PMID 32649883) the reviewer mentioned as well as the others[8-10]. We also think that the reviewer raised an important point that "condensates of TDP-43 can be a double-edged sword in neurodegeneration". Dynamic condensation process of TDP-43 is protective, while disruption of the dynamic process may result in TDP-43 solid aggregation which is closely associated with disease. Since we showed that GQDs can decrease both dynamic and solidified TDP-43 condensation (Figure 3c-n, Figure 5 c-o), it will be important to apply GQDs for the cells in diseased and aging condition with dynamic-impaired TDP-43 condensate and aggregates for potential disease treatment.

Taking the reviewer's valuable comments into account, we revised our manuscript to present a more balanced view. This includes acknowledging the dual nature of TDP-43 nuclear bodies, which involves both their protective function and their potential pathological activity once they are converted into pathological aggregates, as well as the limitations of applying GQDs in modulating TDP-43 condensation.

Accordingly, we added the following discussion to our manuscript in the revised "Introduction" section paragraph 2, as follows:

"……*Moreover, previous studies show that TDP-43 can form dynamic nuclear bodies (NBs) in nucleus modulated by the lncRNA NEAT1 and Hsp70 in response to stress, playing a protective role under normal conditions*[15-17]. *While, the dynamic TDP-43 NBs may be solidified under chronic stress or diseased condition*[15,16]. *The disruption of TDP-43 functions, particularly through aberrant phase separation and aggregation in the nucleus, may contribute to ALS and FTD pathogenesis*[10]……"

As well as in the revised Discussion, paragraph 2 and 3, as follows:

"……*highlighting the pathological relevance of TDP-43 condensation in nucleus*…… *Of note, in addition to nuclear TDP-43 condensation, our designed GQDs also exhibits potential activity in*

modulating the cytoplasmic TDP-43 condensates, which broadens the application of GQDs in regulating protein condensation in both cytoplasm and nucleus at the same time.

Previous work showed that TDP-43 formed dynamic NBs in the nucleus in response to stress, playing an essential protective role in the cellular stress response[15,16]*. The transiently formed TDP-43 NB can be disassembled during recovery after stress. While, under chronic stress, TDP-43 NB could be further solidified and loss capability for dynamic disassembly, which is believed to play a pathological role in disease. Thus, the properties and roles of TDP-43 condensates vary significantly, serving protective functions in normal stress responses while potentially becoming detrimental in disease states. We showed that the designed GQDs can inhibit the formation of both the dynamic condensate and solidified condensates of TDP-43 in nucleus (Figure 3c-n, Figure 5 c-o). Thus, for potential therapeutic application, it will be important to apply GQDs to the cells with solidified TDP-43 condensates and aggregates rather than to those with dynamic TDP-43 NBs, in order to diminish the potential side-effect GQDs in disrupting the functional TDP-43 NBs in normal cells. Moreover, development of next generate GQDs which could specifically target the pathological TDP-43 condensates will be of importance.*"

Regarding TDP-43 phosphorylation, we also agree with the reviewer that the role of TDP-43 phosphorylation is ambiguous. We apologize for any lack of clarity in our manuscript. We specifically used antibodies for TDP-43 phosphorylated at sites S409/410, as phosphorylation at these sites was commonly used as a disease-specific marker in TDP-43 pathological aggregates[11, 12]. We appreciate the reviewer's input on this matter and ensured that all TDP-43 phosphorylation in our revised text is clearly marked as S409/410.

Technical comments

1. Quality of cell images is low therefore it is not always possible to appreciate the phenotypes described. In several cases, there is a mismatch between the claims in the text and the images, controls are missing and sometimes it is not clear what was quantified and how:

Response: We thank the reviewer for raising this point. Following the reviewer's suggestion, we re-examined our image data and replaced the original ones with higher resolution and clarity versions wherever applied (Figure 3, 5, S2, S3, S4, S10, S11). Controls are supplemented in the following question. Moreover, we revised the figure legends and methods section to provide more clarity.

- Fig3 – non-arsenite treated controls are missing, so nuclear body assembly cannot be assessed. In panel h, PBS and GQD treated cells look same to me. Overall, the level of granulation after stress cannot be assessed without a control. How do authors know that stress has worked?

Response: We thank this reviewer's helpful suggestions. Regarding the absence of non-arsenite treated controls in Figure 3, this is critical in assessing nuclear bodies assembly under normal conditions. In our experiments, we consistently included these controls, but erroneously omitted them from the figure. We appreciate very much for the reviewer's suggestions, which have prompted us to include this crucial data in our manuscript. We added new images (Figure R1-1 a-b) to our manuscript, showing that cells transfected with TDP-43 HA plasmid and not subjected to stress form very few nuclear bodies. This is a stark contrast to cells stimulated with sodium arsenite or sodium chloride.

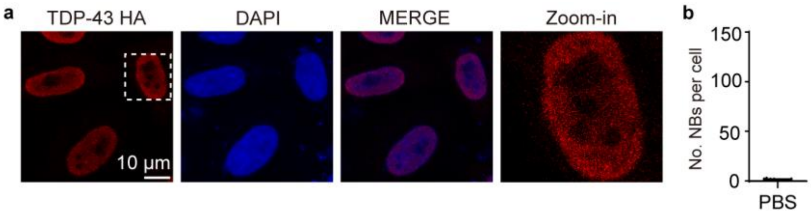

Figure R1-1. Cells without stimulation form few nuclear bodies on TDP-43 HA. (a) Representative confocal images of HeLa cells transfected with TDP-43 HA. Cells were not stimulated with any agents. Scale bar, 10 µm. (b) Quantifications of HeLa cells forming TDP-43 nuclear bodies without stress. Data correspond to the mean ± SD, n=15.

Accordingly, in the revised manuscript, we included Fig. R1-1 as Supplementary Figure S4 a-b. It is described in the revised Results section, "GQDs-NH_2 and GQDs-OH inhibit TDP-43 condensation in the cytoplasm but not in the nucleus" paragraph 1, as follows:

"*……In the absence of stress, cells transfected with TDP-43 HA form very few nuclear bodies (Figure S4 a-b).*"

We apologize for any confusion caused by the compressed image quality in our original submission. Following the reviewer's comments, we uploaded a new set of high-resolution images (Figure 3, 5, S2, S3, S4, S10, S11) to clarify these differences.

- *Fig3c,d – GQD seem to downregulate TDP-43, is this really the case?*

Response: We thank this reviewer's helpful comment. To address the reviewer's question on whether GQDs downregulates the expression of TDP-43, we performed a western blot analysis, under the same experimental conditions as in Figure 3c,d. The results show that GQDs do not affect the expression level of TDP-43 protein (Figure R1-2. a-b). The apparent reduction in TDP-43 level observed in the fluorescence images is due to the high concentration of TDP-43 in the nuclear bodies, which results in a more intense fluorescence. We value reviewer's feedback, which has led to a meaningful addition to

our manuscript.

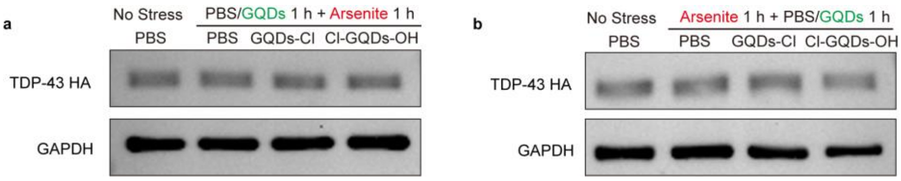

Figure R1-2. GQDs-Cl and Cl-GQDs-OH do not affect the expression of TDP-43. (a-b) Western blot analysis showing TDP-43 expression in the transfected HeLa cells, consistent with the conditions described in Figure 3c, d. GAPDH serves as a loading control.

Accordingly, in the revised manuscript, we added Figure R1-2 as Supplementary Figure S10 c-d, and described it in the revised Results, section “Halogen atom-doped GQDs prevent formation of TDP-43 granules and disassemble them in cells” paragraph 1, as follows:

“*……To ensure halogen atom-doped GQDs does not affect the expression of TDP-43, we conducted a western blot assay. The results demonstrate that halogen atom-doped GQDs do not alter the expression level of TDP-43 protein (Figure S10 c-d)……*”

- *Fig3 – No. granules per cell was quantified for both nuclear bodies and SGs, was analysis done in the nucleus and cytoplasm separately?*

Response: We sincerely thank the reviewer for his/her question about the granule quantification in Figure 3 of our manuscript. We apologize for any lack of clarity in our initial description. We appreciate this opportunity to elucidate our methodology. For nuclear bodies, quantification was performed specifically on TDP-43 positive granules within the nucleus of cells. In contrast, for SGs, our focus was on quantifying TDP-43 positive granules within the cytoplasm. We meticulously reviewed each figure and caption to ensure that the granules we quantitatively analyzed are distinctly delineated. We are grateful for reviewer’s insightful feedback, which has significantly contributed to enhancing the clarity of our results. We revised Figure 3, S2, S9, S10, and corresponding Figure legend. As an example, I revised Figure 3 as follows:

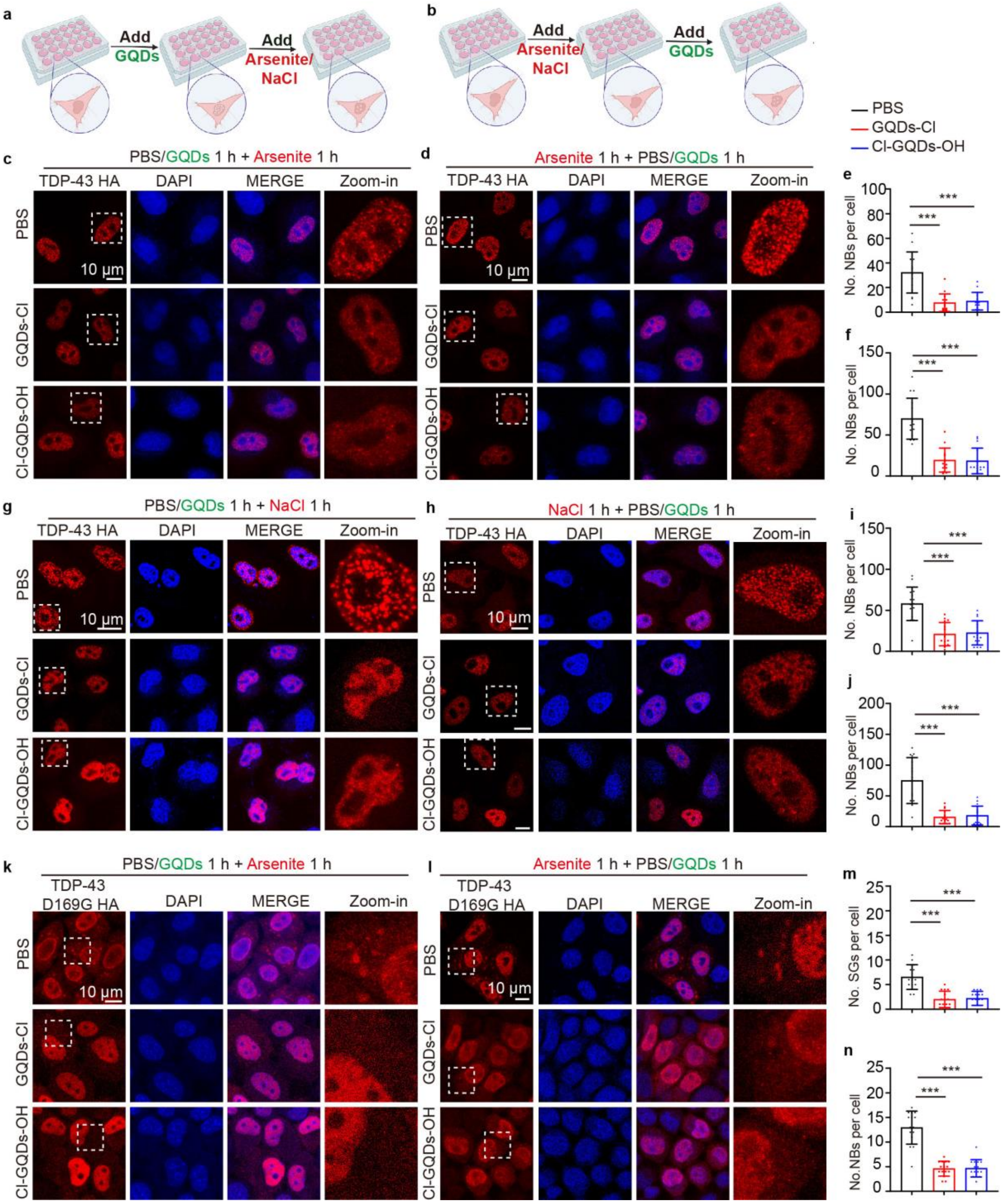

Figure 3. Halogen atom-doped GQDs disassemble preformed TDP-43 granules as well as prevent their formation in the nucleus and cytoplasm. (a) Schematic diagram of the assay for GQDs inhibition of LLPS of TDP-43. (b) Schematic diagram of the TDP-43 granules disassembly assay. (c) Representative confocal images of HeLa cells transfected with TDP-43 HA. Cells were treated with 0.1 μg μL^{-1} of GQDs or PBS (control) for 1 h and then treated with 250 μM arsenite for 1 h. Scale bar, 10 μm. (d) Representative confocal images of HeLa cells transfected with TDP-43 HA. Cells were treated with 250 μM sodium arsenite for 1 h and then treated with 0.1 μg μL^{-1} of both types of GQDs or PBS (control) for 1 h. Scale bar, 10 μm. (e-f) Quantitative analysis of the number of NBs in nucleus per cell for images c and d. Data correspond to the mean ± SD, n = 15. Student's t test, ***$P < 0.001$. (g) Representative confocal images of

HeLa cells transfected with TDP-43 HA. Cells were treated with 0.1 μg μL^{-1} of GQDs or PBS (control) for 1 h and then treated with 300 mM NaCl for 1 h. Scale bar, 10 μm. (h) Representative confocal images of HeLa cells transfected with TDP-43 HA. Cells were treated with 300 mM NaCl for 1 h and then treated with 0.1 μg μL^{-1} of both types of GQDs or PBS (control) for 1 h. Scale bar, 10 μm. (i-j) Quantitative analysis of the number of NBs in nucleus per cell for image g-h. Data correspond to the mean ± SD, n = 15. Student's t test, ***P < 0.001. (k) Representative confocal images of HeLa cells transfected with TDP-43 D169G HA. Cells were treated with 0.1 μg μL^{-1} of GQDs or PBS (control) for 1 h and then treated with 250 μM arsenite for 1 h. Scale bar, 10 μm. (l) Representative confocal images of HeLa cells transfected with TDP-43 D169G HA. Cells were treated with 250 μM sodium arsenite for 1 h and then treated with 0.1 μg μL^{-1} of both types of GQDs or PBS (control) for 1 h. Scale bar, 10 μm. (m-n) Quantitative analysis of the number of SGs in cytoplasm per cell for image k-l. Data correspond to the mean ± SD, n = 15. Student's t test, ***P < 0.001.

- Fig5c – top (control) panel, image is hugely overexposed, does it mean that the expression level is significantly higher? The resolution and magnification are quite low so it is impossible to tell whether these are nuclear bodies or apoptotic/pyknotic cells. What does "aggregation cell" readout mean?

Response: Thank the reviewer for raising this point. We sincerely apologize for the overexposure and collected additional images with the correct exposure. To clearly distinguish if TDP-43 K181E RFP cells are apoptotic, we improved the magnification in our new images (Figure R1-3 a-b). Additionally, we performed Caspase 3 antibody staining to identify apoptotic cells (Figure R1-3 c). These new data further support that the overexpression of the K181E mutant TDP-43 does not correlate with apoptosis in our cell models.

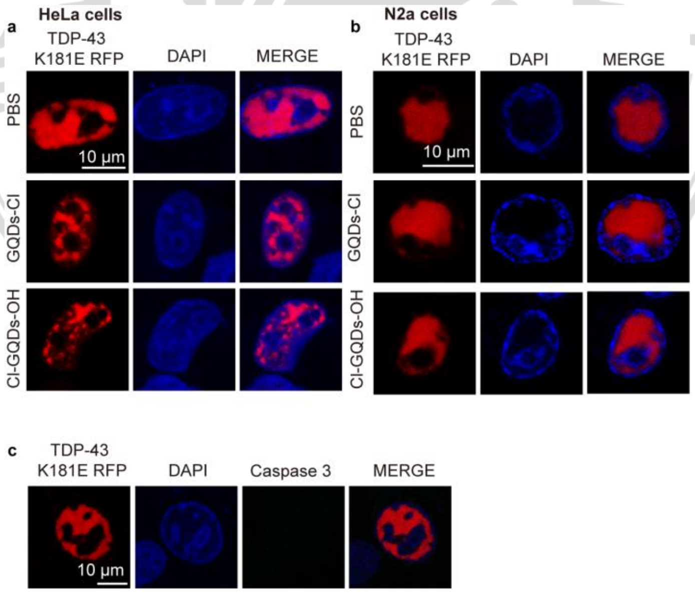

Figure R1-3. Halogen atom-doped GQDs inhibit the aggregation of TDP-43. (a,b) Magnified views of Figures 5c and 5d, respectively. (c) Fluorescence images of HeLa cells transfected with TDP-43 K181E RFP and stained with Caspase 3 antibodies. Scale bar: 10 μm.

In the revised manuscript, we included Figure R1-3 as Supplementary Figure S13, described in the Results section "Halogen atom-doped GQDs can inhibit aggregation of TDP-43 and decrease pTDP-43 (S409/410) level", as follows:

"……*Magnified images and Caspase 3 antibody staining show that the cells do not undergo apoptosis (Figure S13 a-c)*……"

Moreover, consistent with previous reports[13], cells overexpressing K181E TDP-43 exhibited decreased mobility (Figure R1-4 a-b) and colocalization with the disease-related marker pTDP-43 (409/410) (Figure R1-4 c). Threrfore, in our study, we describe it as "aggregation cell"[14, 15].

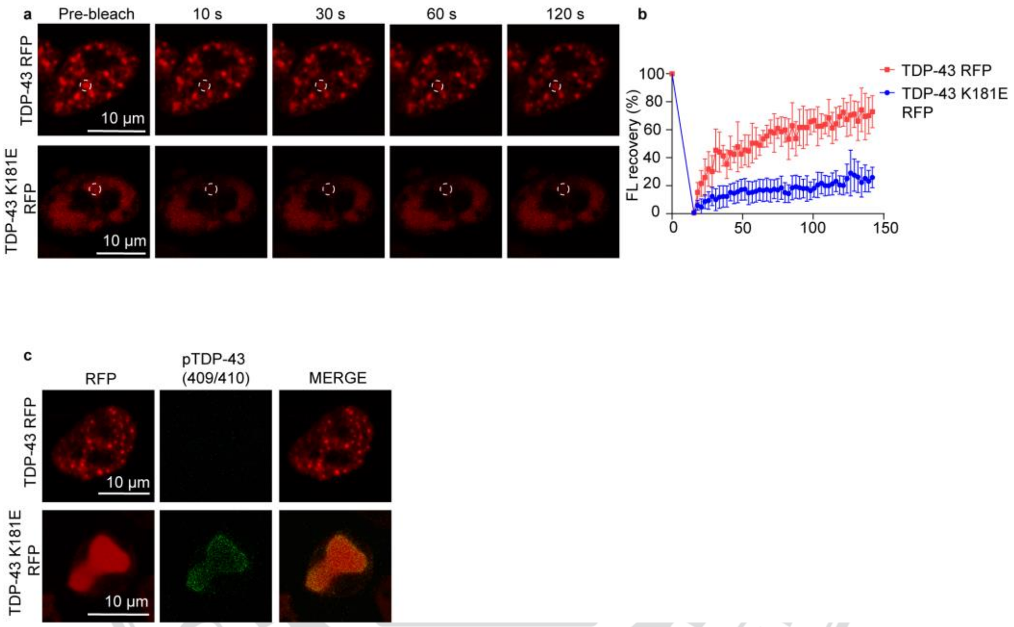

Figure R1-4 Aggregation of TDP-43 K181E in cells. (a) Representative fluorescence microscopy images showing TDP-43 K181E RFP in HeLa cells, with regions (dashed circles) photobleached in the FRAP assay at indicated times. (b) Fluorescent intensity recovery curves are shown, n=5. (c) Representative images of 293T cells expressing TDP-43 RFP or TDP-43 K181E RFP plasmid, co-immunostained for pTDP-43 (409/410).

In the revised manuscript, we included Figure R1-3 as Supplementary Figure S5, described in the Results section "GQDs-NH_2 and GQDs-OH inhibit TDP-43 condensation in the cytoplasm but not in the nucleus", as follows:

"……*Nuclear aggregation of TDP-43 K181E exhibits slower recovery in the FRAP assay (Figure S5 a-b) and is hyperphosphorylated (S409/410) (Figure S5 c)*……."

- *Fig5, why TDP-43 total levels not provided here and moved to supplementary? It is essential to have them side by side.*

Response: We thank for this reviewer’s suggestion. Following the reviewer’s suggestion, we revised the figure 5. The total levels of TDP-43, previously included in the supplementary materials, are now incorporated directly into Figure 5.

- *FigS4 – no quantification.*

Response: Thanks the reviewer for pointing out the absence of quantification in original Figure S4 of our manuscript. We performed a comprehensive quantification analysis for original Figure S4 and updated the supplementary materials accordingly.

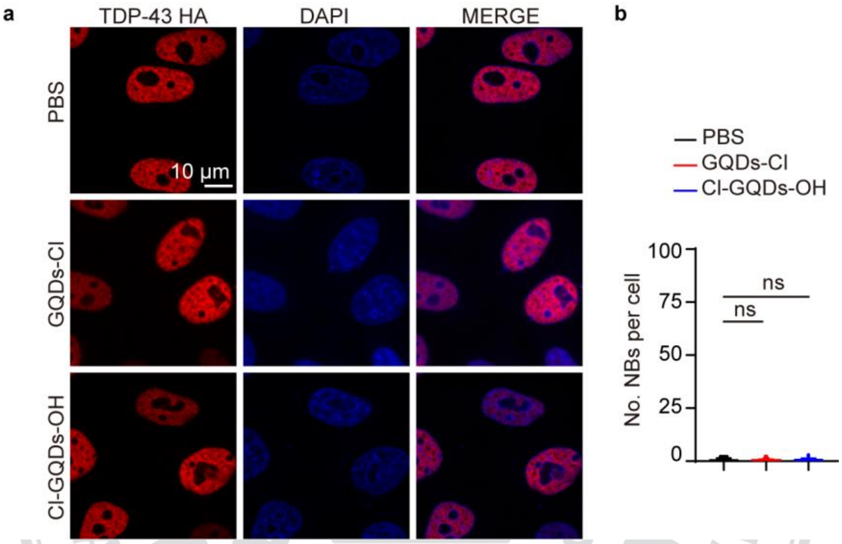

Figure S10. Halogen atom-doped GQDs alone do not induce the nuclear bodies assembly. (a) Representative confocal images of HeLa cells transfected with TDP-43 HA plasmid, followed by incubation with 0.1 μg μL^{-1} GQDs for 2 h. Scale bar, 10 μm. (b) Quantitative analysis of the number of nuclear bodies per cell in image a. Data correspond to the mean ± SD, n = 15. Student’s t test, ns: not significant.

2. Line 95: It should be specified what type of stress is being used and that 169 mutant does not form SGs in naïve cells. Does GQD also prevent cytoplasmic mislocalisation of TDP-43 169? This should be measured, to prove that it is condensation that is affected by GQDs rather than cytoplasmic levels of the protein.

Response: We sincerely thank this reviewer for his/her helpful suggestions. We updated the manuscript to specify the type of stress applied to the D169G mutant of TDP-43 at Line 95 as follows:

“*……The D169G mutation, originally identified in a sporadic ALS case, significantly increases translocation of TDP-43 into the cytoplasm and formation of SGs under sodium arsenite stress conditions (Figure S3 a-b)……*”

Furthermore, we added control experiments for the D169G mutant without stress in the

supplementary section. The results show that unstimulated cells form significantly fewer SGs compared to cells stimulated by sodium arsenite (Figure R1-5 a-b).

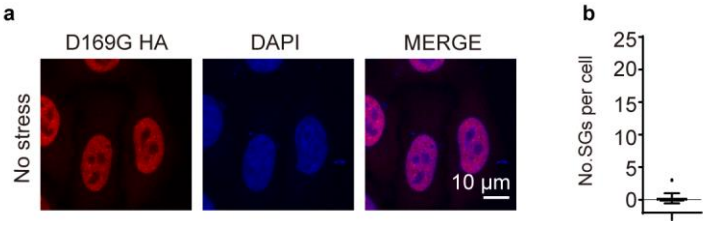

Figure R1-5. Minimal stress granule formation in TDP-43 D169G HA without stimulation. (a) Representative confocal images of HeLa cells transfected with TDP-43 D169G HA. Cells were not stimulated with any agents. Scale bar, 10 μm. (b) Quantification of TDP-43 D169G HA HeLa cells forming SGs without stress. Data correspond to the mean ± SD, n=15.

In the revised manuscript, we included Figure R1-5 as Supplementary Figure S3 and described it in the Results section, "GQDs-NH_2 and GQDs-OH inhibit TDP-43 condensation in the cytoplasm but not in the nucleus," as follows:

"……*Unstimulated TDP-43 D169G transfected HeLa cells form minimal SGs (Figure S3 c-d)*……"

Following the reviewer's suggestion, we conducted western blot experiments to determine whether both GQDs-NH_2 and GQDs-OH influence the localization of TDP-43 D169G in the cytoplasm and nucleus. We measured the expression levels of TDP-43 D169G in separated cytoplasmic and nuclear fractions. The results indicate that neither GQDs-NH_2 nor GQDs-OH affect the distribution of TDP-43 D169G between the cytoplasm and nucleus (Figure R1-6 a-b).

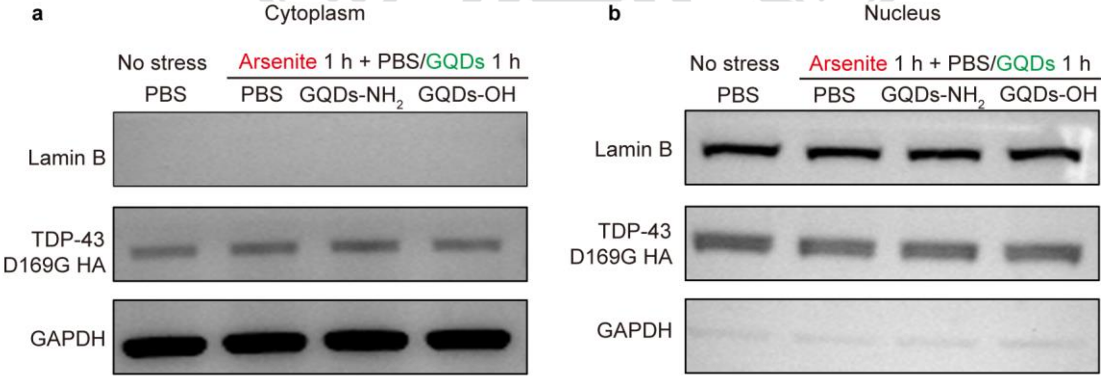

Figure R1-6. Localization of TDP-43 D169G in the cytoplasm and nucleus is unaffected by GQDs-NH_2 and GQDs-OH. (a-b) Western blot analysis of TDP-43 D169G HA expression in cytoplasmic and nuclear fractions of transfected HeLa cells. Cells were treated with 250 μM sodium arsenite for 1h, followed by 0.1 μg μL^{-1} GQDs-NH_2 or GQDs-OH or PBS (control) for 1 h. GAPDH serves as a cytoplasm loading control, and Lamin B serves as a nuclear loading control.

Accordingly, in the revised manuscript, we added Figure R1-6 as Supplementary Figure S3, and described it in the Results section, "GQDs-NH_2 and GQDs-OH inhibit TDP-43 condensation in the cytoplasm but not in the nucleus" as follows:

"*……To determine if the decrease in SGs is due to GQDs preventing cytoplasmic mislocalization of TDP-43 D169G, we separated the cytoplasmic and nuclear fractions and measured the expression levels of TDP-43 D169G. The results indicate both GQDs-NH_2 and GQDs-OH do not affect the distribution of TDP-43 D169G between the cytoplasm and nucleus (Figure S3 e-f)……*"

3. Line 230: this is over-simplification. N-terminal domain of TDP-43 (NTD) is important for its physiological oligomerisation and functionality.

Response: We thank the reviewer for raising this point. We agree with the reviewer it is important to consider the interactions between the various domains of TDP-43 and GQDs. To fully address this, we conducted additional BLI experiments to examine the binding ability of halogen atom-doped GQDs to various domains of TDP-43. The results demonstrate that halogen atom-doped GQD interact with various domains of TDP-43, including the NTD, RRM, and LC domain (Figure R1-7 a-f). The K_D of GQDs-Cl and Cl-GQDs-OH to the TDP-43 NTD was measured as 19.60 μM and 10.78 μM, respectively. These GQDs also exhibit a similar binding affinity to the TDP-43 RRM domain, with K_D values of 18.96 μM and 13.33 μM. For the TDP-43 LC domain, BLI data revealed that halogen atom-doped GQDs bind with K_D values of 1.50 μM and 1.35 μM, suggesting the strongest binding affinity among the three domains, potentially attributable to its flexible structure.

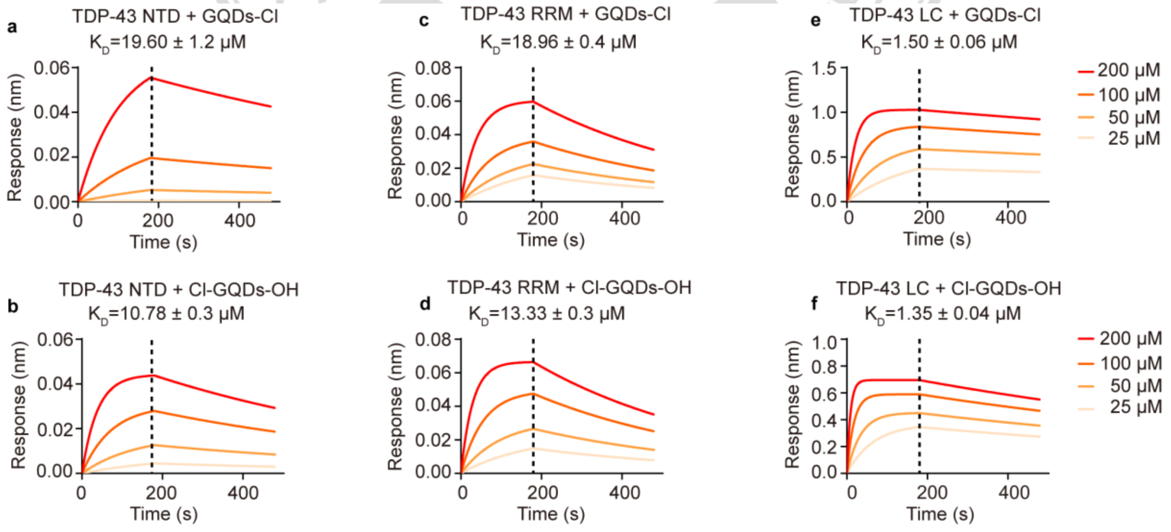

Figure R1-7. Binding affinity of halogen atom-doped GQDs to various TDP-43 domains. (a-f) Kinetic binding curves of TDP-43 NTD, RRM, LC domain with a concentration gradient of GQDs-Cl or Cl-GQDs-OH. The association and dissociation profiles are divided by a vertical black dotted line.

According to reviewer's suggestion, we revised our manuscript to include a more comprehensive discussion of the different domains of TDP-43 in GQDs binding. In the revised manuscript, we included Figure R1-7 as Supplementary Figure S12, described in the Results section, "Halogen atom-doped GQDs inhibit TDP-43 LC fibrillation" as follows:

"*TDP-43 features an N-terminal domain with dimerization propensity, two RRM domains with β-α-β motifs, and an LCD domain, mediating liquid-to-solid phase transitions and pathological fibril formation*[37,38]*. BLI assays were used to quantify the binding affinity between halogen atom-doped GQDs and different domains of TDP-43. The results demonstrate that halogen atom-doped GQD interact with various domains of TDP-43, including the NTD, RRM, and LC domain, with different binding affinities (Figure S12 a-f). The* K_D *of GQDs-Cl and Cl-GQDs-OH to the TDP-43 NTD was measured as 19.60 μM and 10.78 μM, respectively. These GQDs also exhibit a similar binding affinity to the TDP-43 RRM domain, with* K_D *values of 18.96 μM and 13.33 μM. Notably, for the TDP-43 LC domain, BLI data revealed that halogen atom-doped GQDs bind with* K_D *values of 1.50 μM and 1.35 μM, suggesting the strongest binding affinity among the three domains, potentially attributable to its flexible structure……*"

4. A toxicity readout for cells with decreased TB assembly would be required.

Response: Sorry that we have some confusion about the meaning of "TB" as mentioned in the comment. In our study, we focused on Nuclear Bodies (NB) modulation. We thought TB might be a typo, which refers to nuclear body. To address this, we included results from toxicity tests related to NB assembly in our manuscript. The tests were conducted to examine the potential cytotoxic effects under non-stress conditions, arsenite stimulation, and GQDs supplementation post-arsenite stimulation. Our results indicate that neither treatment exhibited obvious toxicity (Figure R1-7).

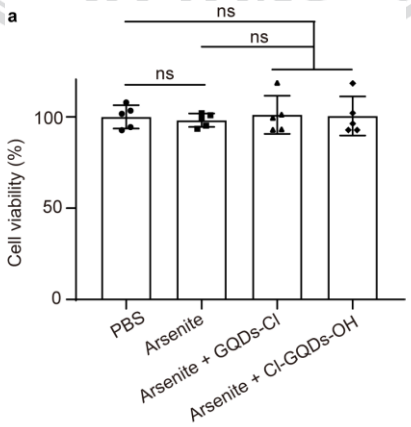

Figure R1-7. Cytotoxicity analysis in NB assembly under arsenite stimulation with or without GQDs-Cl and Cl-GQDs-OH. (a) Cell viability was assessed in cells treated with 250 μM sodium arsenite

for 1 hour, followed by 0.1 μg μL^{-1} halogen atom-doped GQDs or PBS (control) for 1 h. Cell viability analysis measured by CCK8 assay. Data correspond to the mean ± SD, n = 5. Student's t test, ns: not significant.

According to reviewer's suggestion, we included Figure R1-7 as Supplementary Figure S10 e, described in the Results section, "Halogen atom-doped GQDs inhibit TDP-43 LC fibrillation" as follows:

"*……Meanwhile, we conducted toxicity tests to examine potential cytotoxic effects under non-stress conditions, arsenite stimulation, and GQDs supplementation post-arsenite stimulation. Our results show that neither treatment exhibited obvious toxicity (Figure S10 e)……*"

Reviewer #2 (Remarks to the Author):

In the manuscript, Dai et al. reported that the function of neurodegenerative diseases-related protein TDP-43 could be manipulated by functionalized graphene quantum dots (GQDs) during the LLPS process. Considering the intricacies of nuclear phase separation and aggregate elimination, an in-depth research of nucleus-localized TDP-43 still remains challenging. Herein, the halogen-doped GQDs showed superior nucleus-targeted property which could prevent the construction of both TDP-43 nuclear bodies and TDP-43-positive stress granules. What's more, GQDs also showed great potential in restraining both aggregation and phosphorylation levels within the nucleus and cytoplasm. From my perspective, this manuscript is interesting and shows potential for the neurodegenerative diseases-related therapy. Thus, I recommend this manuscript for publication after minor revisions.

Response: We sincerely thank the reviewer for the positive and constructive feedback on our work. We followed the reviewer's insightful suggestions and perform substantial experiments to fully address them.

For brevity, the concerns are listed below:

1. The author showed that the introduction of chlorine atoms into GQDs increased nucleus uptake. What's the mechanism of nucleus uptake progress promoted by chlorine-doped GQDs? Does the amount of chlorine atoms affect the uptake behavior of nucleus?

Response: We are grateful to the reviewer for raising this important point. Regarding the mechanism by which chlorine-doped GQDs increase nuclear uptake, we propose the following two aspects:

1. **Amphiphilicity**: Edge-functionalization of GQDs with Cl ligands imparts amphiphilic properties, enhancing their interaction with lipid membranes and thus facilitating nucleus entry [16, 17].
2. **Halogenation Surface Features**: Halogenation improves membrane interaction capabilities of GQDs, leading to increased binding affinity and permeation across membranes[18].

To investigate the effect of the amount of chlorine atom on cellular uptake behavior, we conducted further experiments (Figure R2-1). These experiments indicate that the amount of chlorine atoms has an influence on cellular uptake. Additionally, we observed enhanced toxicity correlated with an increase in the amount of chlorine atoms (Figure S6 g). Therefore, our study focused on the effects of GQDs-Cl and Cl-GQDs-OH on TDP-43 protein, aiming to balance efficacy and toxicity.

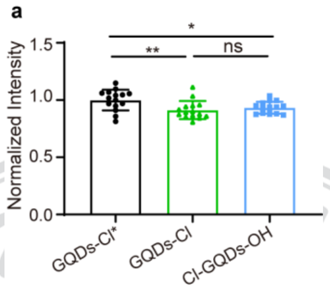

Figure R2-1. Ligand functionalized GQDs mediate delivery to the nucleus. (a) Normalized fluorescence intensity of HeLa cells (normalized to its own fluorescence). Data correspond to the mean ± SD, n =15. Student's t test, **P < 0.01; *p<0.1; ns: not significant.

In our manuscript, we included the Figure R2-1 to Figure S6 f, and describe it at Result section, "Design and characterization of nucleus-penetrating halogen atom-doped GQDs" paragraph 1, as follows:

"……And the higher amount of the chlorine atom, the greater the uptake of GQDs into the nucleus (Figure S6 f)……"

2. As an emerging bio-nanomaterial, GQDs have displayed promise in treating various diseases. the authors should give a comprehensive overview regarding the advances on design and applications of GQDs, close-related recent references in this field, such as Chem. Rev., 2019, 119, 4357-4412; Angew. Chem. Int. Ed. 2019, 58, 7087–7091; Angew. Chem. Int. Ed.,2015, 54, 7176-7180; Mater. Today, 2013, 16, 433-442 etc., should be considered to cite; this would be very helpful for understanding how to design and develop suitable GQDs for bioapplications.

Response: We thank the reviewer for the insightful and helpful suggestions. We totally agree that providing a comprehensive overview of the advances in the design and applications of GQDs would greatly enrich our manuscript. Following the suggestion, we included a more detailed discussion of the recent advances in GQDs design and applications and reference the following key references to provide a more complete discussion: Chem. Rev., 2019, 119, 4357-4412; Angew. Chem. Int. Ed., 2019, 58, 7087–7091; Angew. Chem. Int. Ed., 2015, 54, 7176-7180; Mater. Today, 2013, 16, 433-442.

Accordingly, in the revised manuscript, we added discussion in the revised Discussion, paragraph 2 as follows:

"……*In the evolving landscape of nanotechnology, GQDs stand out as a revolutionary innovation. These zero-dimensional nanomaterials are recognized for their exceptional electronic, optical, and chemical properties*[47]*. GQDs are synthesized using both top-down methods, which involve segmenting larger graphene sheets, and bottom-up approaches, employing benzene derivatives or carbonization processes*[48,49]*. This flexibility in synthesis allows for the precise control of GQDs' characteristics, including size, edge functionalization, surface features, activity, biocompatibility, and permeability through the blood-brain barrie*[19, 50-52]*. Such control has been pivotal in optimizing their properties for specific applications, including bioimaging, drug delivery, photodynamic therapy, and nanozymes catalysis*[53,54]*. Given these attributes, GQDs hold considerable promise in the development of chaperone-like nanomaterials for NDs intervention and diagnosis*[55,56]."

3. It is convincing that GQDs modulate the LLPS through electrostatic and hydrophobic interactions. The comparation of binding affinity of GQDs with or without halogen should be provided. Besides, the zeta potential of different GQDs are also suggested to be supplied.

Response: Thank the reviewer for pointing this out. Taken the reviewer's suggestion, we compared the binding affinity of GQDs with and without halogen modification. Our results demonstrate that GQDs not modified with chlorine exhibit a slightly stronger binding affinity to TDP-43 protein compared to GQDs that are chlorine-modified (Figure R2-1). However, it is important to note that without halogen modification, similar with GQDs-OH and GQDs-NH_2 mentioned in our previous study[19], GQDs cannot penetrate the nucleus to interact with TDP-43 proteins.

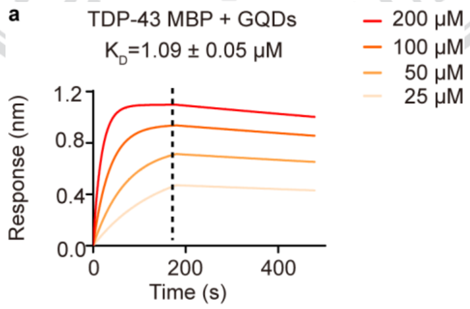

Figure R2-1. Binding affinity of unmodified GQDs to TDP-43 MBP protein. (a) Kinetic binding curves of TDP-43 MBP with a concentration gradient of GQDs not modified with chlorine. The association and dissociation profiles are divided by a vertical black dotted line.

In addition, we appreciate reviewer's recommendation to include zeta potential measurements for different GQDs. Acknowledging the importance of zeta potential in understanding the surface charge characteristics of GQDs, we conducted these measurements.

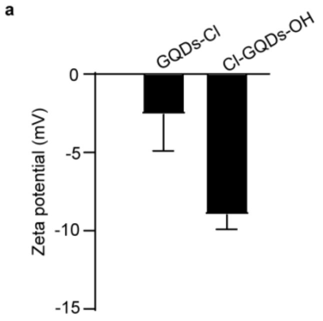

Figure R2-2. Characterization of zeta potentials of GQDs. (a) Zeta potential of GQDs-Cl and Cl-GQDs-OH in 50mM Tris, 150 mM NaCl. Data correspond to the mean ± SD, n=3

According to reviewer's suggestion, we included Figure R2-2 as Supplementary Figure S8 a, described in the Results section, "Halogen atom-doped GQDs modulate the phase separation of TDP-43 in vitro through electrostatic and hydrophobic interactions" as follows:

"*……The mean zeta potentials of GQDs-Cl and Cl-GQDs-OH were found to be -2.58 mV and -9.00 mV, respectively (Figure S8 a).*"

4. The author claimed that the interaction between TDP-43 LC and GQDs was likely to be weak and transient, and non-specific to any particular region or residue of the TDP-43 LC domain. Considering the intricate biological environment which may disrupt the weak interaction and the potential off-target toxicity to other proteins, how does the author consider this issue?

Response: Thanks for pointing this out. We agree with the reviewer that interaction between TDP-43 and GQDs was likely to be weak and transient, does indeed represent a limitation of our study. To address, we revised the manuscript by additional discussion on the limitation at the paragraph 3, Discussion section, as follows:

"*Finally, several limitations of applying GQDs in cells for modulating protein condensation must be considered. First, the complexity of MLOs in cells, containing a variety of different proteins and nucleic acids, creates challenges. The GQDs' broad interaction with many different intracellular biomacromolecules necessitates further optimization of GQDs with specific client-recognition capability[17,18]. Moreover, due to the complexities associated with the absorption, metabolism, excretion, and immunogenicity of GQDs, it remains uncertain whether the in vitro and cellular effects of GQDs observed in this study can be replicated in the in vivo models of TDP-43-related NDs. Nevertheless, our research offers vital insights into GQDs' potential as modulators of TDP-43 condensation in the*

context of ND treatment."

While our study suggests that GQDs do not exhibit specific binding to any particular region or residue of the TDP-43 LC domain, we observed differential binding affinities of GQDs to various TDP-43 domain, including NTD, RRM and LC domain. The K_D of GQDs-Cl and Cl-GQDs-OH to the TDP-43 NTD was measured as 19.60 μM and 10.78 μM, respectively. These GQDs also exhibit a similar binding affinity to the TDP-43 RRM domain, with K_D values of 18.96 μM and 13.30 μM. For the TDP-43 LC domain, BLI data revealed that halogen atom-doped GQDs bind with K_D values of 1.50 μM and 1.35 μM, suggesting the strongest binding affinity among the three domains (Figure R2-3). This observation underscores the potential for GQDs to have specific interactions, albeit with limitations in specificity at the residue level.

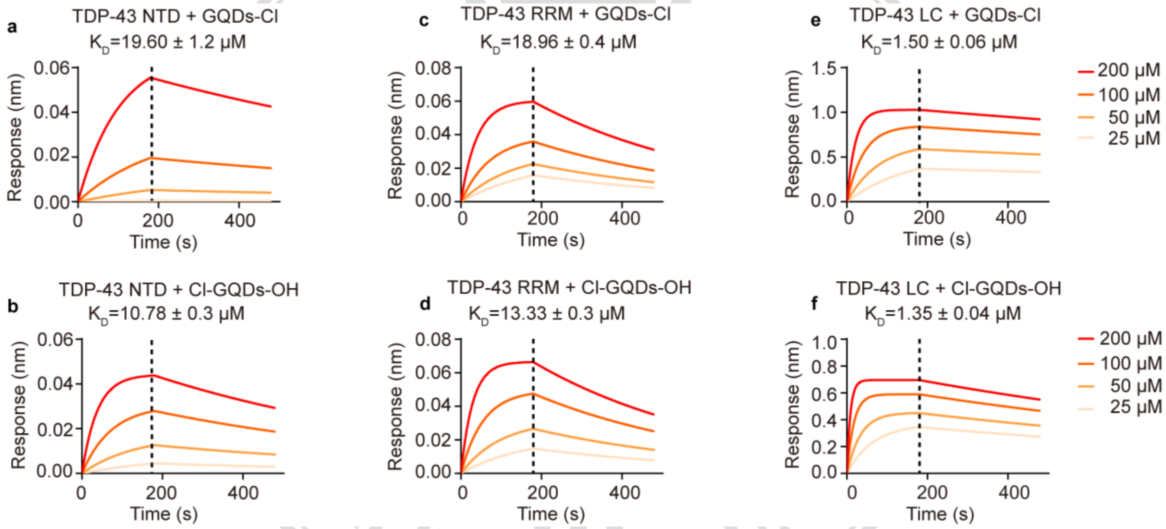

Figure R2-3. Binding affinity of halogen atom-doped GQDs to various TDP-43 domains. (a-f) Kinetic binding curves of TDP-43 NTD, RRM, LC domain with a concentration gradient of GQDs-Cl or Cl-GQDs-OH. The association and dissociation profiles are divided by a vertical black dotted line.

Regarding potential off-target toxicity, many studies have shown that GQDs have excellent bio-compatibility[20, 21]. Previous studies showed that GQDs do not cause appreciable toxicity in treated animals[22, 23]. No obvious organ damage or lesions were observed in the GQDs-treated mice. The GQDs did not exhibit notable toxicity, biological interaction, or inflammatory symptoms in the liver, kidney, heart, or spleen, as observed by histological analysis[22]. Furthermore, nanographene sheets can be gradually cleared through renal and fecal excretion[24]. Collectively, these pieces of evidence underscore the biomedical application potential of GQDs.

Reviewer #3 (Remarks to the Author):

It is a very convincing manuscript by Li, Bai and coworkers on the use of graphene quantum dots to manipulate the formation of TDP-43 aggregation in cellulo.

TDP-43 is a crucial protein implied in numerous processes associated with RNA interaction and it has been proposed that the protein play a key role in several neurodegenerative disorders through its aggregation into amyloid fibrils and phase separation. The protein is very challenging, because unlike abeta, the sequence is much longer and contain several functional (and structurally folded) domains in its native state.

The authors present a long list of experimental results showing a significant effect of graphene quantum dots, especially the potential use of GQD in the nucleus, to manipulate the protein aggregation state. I recommend the manuscript for publication, however I have two main comments on the sample preparation and the use of the low complexity construct, as well as several minor comments (see below).

Response: We thank the Reviewer very much for recognizing the importance of this work and providing valuable suggestions for us to improve the manuscript. We followed the reviewer's insightful suggestions and performed additional experiments to fully address them.

Main comment:

#1: Some clarification is required concerning the sample preparation of the TDP-43 protein:

. The authors use a non-native TDP-43 construct, that comprises the maltose binding protein fusion protein (MBP). It is unclear if and how MBP might affect the effect of graphene quantum dots. Can the authors comment on this possible MBP - GQD interaction ? Or provide any relevant literature showing that GQD is not affected by the presence of the MBP ?

Response: We are grateful to the reviewer for raising this important point. To address the reviewer's concern, we performed a BLI assay to assess the direct interaction between TDP-43 protein and halogen atom-doped GQDs. The results revealed that the K_D values for the interaction between TDP-43 and halogen atom-doped GQDs are 4.80 μM and 1.35 μM (Figure R3-1). These values are comparable to the KD values observed for the interaction between TDP-43 MBP and halogen atom-doped GQDs (Figure 2m-n). Additionally, our cellular model experiments demonstrated the interaction of TDP-43 with GQDs in the absence of the MBP tag (Figure 3c-n). Based on these findings, the MBP tag's effect on the interaction between TDP-43 and GQDs is minimal.

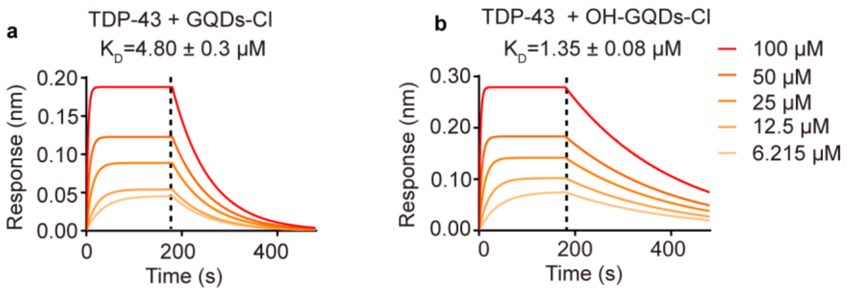

Figure R3-1. Binding affinity of halogen atom-doped GQDs to TDP-43 protein. (a-f) Kinetic binding curves of TDP-43 with a concentration gradient of GQDs-Cl or Cl-GQDs-OH. The association and dissociation profiles are divided by a vertical black dotted line.

Correspondingly, we included Figure R3-1 as Supplementary Figure S9, described in the Results section, "Halogen atom-doped GQDs modulate the phase separation of TDP-43 *in vitro* through electrostatic and hydrophobic interactions" as follows:

"……BLI assays show that TDP-43 interacts with halogen atom-doped GQDs, showing K_D values of 4.80 μM and 1.35 μM (Figure S9 a-b). These values are comparable to the KD values observed for the interaction between TDP-43 MBP and halogen atom-doped GQDs (Figure 2 m-n). This suggests that the MBP tag has a minimal impact on the interaction between TDP-43 and GQDs……"

. Is the MBP also fused with a 6-His tag ? It is unclear from the protocol.

Response: We appreciate the reviewer's attention to detail regarding the fusion of the MBP with a 6-His tag. We apologize for any lack of clarity in our initial description. Taken the reviewer's suggestion, we revised the manuscript to provide a clearer explanation. This revision can be found in the "Method: Protein expression and purification" section, paragraph 1. Specifically, we now state that TDP-43 was cloned into the pET9d vector with a TEV protease cleavable MBP-His tag at the C-terminus. The revised text reads as follows:

"TDP-43 full length was cloned into the pET9d vector with a TEV protease cleavable MBP-His tag at the C-terminus……"

. A "TDP-43 LC" construct is also mentioned in the material and method. Is this construct also attached to MBP ? with 6-His ?

Response: Thanks again. We apologize for any lack of clarity in our initial description. We revised the manuscript to provide a clearer explanation. This revision can be found in the "Method: Protein expression and purification" section, paragraph 2. Specifically, we now state that TDP-43 LC was

cloned into the pET28a vector with a 6-His tag at the N-terminus. The revised text reads as follows:

“*TDP-43 LC was cloned into the pET28a vector with a 6-His tag at the N-terminus……*”

. *What is the exact amino-acid sequence of the LC construct ?*

Response: We thank the reviewer for reminding us to provide the details. The exact amino acid sequence of the human TDP-43 LC (274-414) is as follows:

“GRFGGNPGGFGNQGGFGNSRGGGAGLGNNQGSNMGGGMNFGAFSINPAMMAAAQAA LQSSWGMMGMLASQQNQSGPSGNNQNQGNMQREPNQAFGSGNNSYSGSNSGAAIGWGSAS NAGSGSGFNGGFGSSMDSKSSGWGM”

For comprehensive understanding, we also provide the full-length TDP-43 amino acid sequence (Figure R3-2):

“MSEYIRVTEDENDEPIEIPSEDDGTVLLSTVTAQFPGACGLRYRNPVSQCMRGVRLVEGIL HAPDAGWGNLVYVVNYPKDNKRKMDETDASSAVKVKRAVQKTSDLIVLGLPWKTTEQDLK EYFSTFGEVLMVQVKKDLKTGHSKGFGFVRFTEYETQVKVMSQRHMIDGRWCDCKLPNSKQ SQDEPLRSRKVFVGRCTEDMTEDELREFFSQYGDVMDVFIPKPFRAFAFVTFADDQIAQSLCG EDLIIKGISVHISNAEPKHNSNRQLERSGRFGGNPGGFGNQGGFGNSRGGGAGLGNNQGSNMG GGMNFGAFSINPAMMAAAQAALQSSWGMMGMLASQQNQSGPSGNNQNQGNMQREPNQAF GSGNNSYSGSNSGAAIGWGSASNAGSGSGFNGGFGSSMDSKSSGWGM”

Accordingly, we included Figure R3-2 as Supplementary Figure S1.

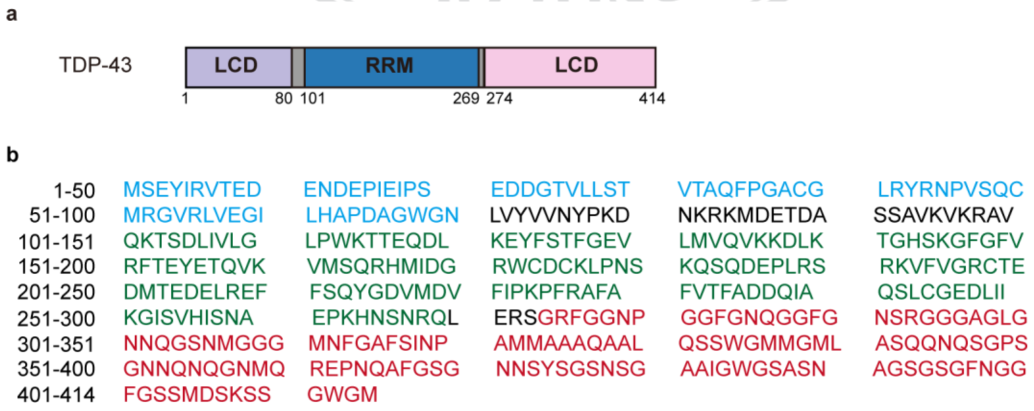

Figure R3-2. Amino acid sequence of full length TDP-43 protein. (a) TDP-43 NTD (1-80) marked with blue, TDP-43 RRM (101-269) marked with green and TDP-43 LC (274-414) marked with red.

. The full-length TDP-43 sequence is known to rapidly be cleaved into smaller C-terminal fragments. A SDS gel of the full-length construct would be needed to clarify if in vitro experiments are done on the full-length protein or on C-terminal fragments.

Response: We thank the reviewer for raising this point. Taken the reviewer's suggestion, we conducted SDS-PAGE analysis on the full-length TDP-43 MBP protein to determine whether it undergoes cleavage into C-terminal fragments under our experimental conditions. These results demonstrate that in our experimental setup, the full-length TDP-43 protein remains intact (Figure R3-3).

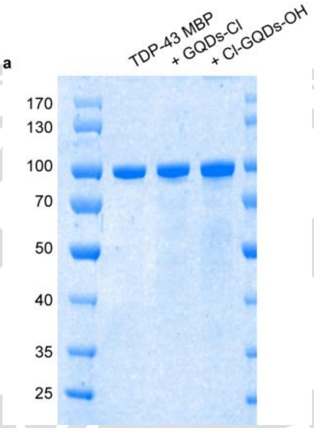

Figure R3-3. SDS-PAGE of TDP-43-MBP protein. (a) SDS-PAGE of TDP-43 MBP at a concentration of 20 μM in a buffer solution (50 mM Tris, pH 7.5, 150 mM NaCl, 10% Dextran 70), both with and without the addition of 0.01 μg/μL GQDs-Cl or Cl-GQDs-OH.

Accordingly, we included Figure R3-3 as Supplementary Figure S7 in the manuscript. In the Result section "Halogen atom-doped GQDs modulate the phase separation of TDP-43 *in vitro* through electrostatic and hydrophobic interactions", we added the following description:

"*……SDS-PAGE analysis confirms that the TDP-43 MBP protein remains intact in our experimental conditions (Figure S7 a).*"

#2: About the ThT experiments on the LC construct: I'm surprised (in a positive way) by the results shown in Figure SI1, panel i and j. TDP-43 LC is known to spontaneously aggregate when concentrated. Here the maximal concentration used is 0.1 mg/ml. I'm surprised to see that TDP-43 LC does not aggregate for almost 1 hour at this concentration. Can the authors comment on that ? A more detailed description of the protocol on (i) the LC construct purification and aggregation and (ii) how and when the time t=0 was fixed in the ThT experiments would be useful.

Response: We are grateful for the reviewer's insightful observation regarding the ThT experiments on the TDP-43 LC construct. The TDP-43 LC protein was purified and concentrated to over 30 mg/ml,

followed by storage in flash-frozen form at -80 °C within an elution buffer containing 50 mM Tris (pH 8.0), 6 M guanidine hydrochloride, and 100 mM imidazole. Before the ThT experiments, the protein was desalted using a desalting column (GE Healthcare, USA) into a buffer containing 20 mM MES, pH 6.0. The protein was not concentrated after desalination. Due to the low concentration of protein used (20μM), there was a lag time of almost 1 hour[8, 25]. The ThT experiment was carried out immediately after desalting.

Following the reviewer's comments, in the revised manuscript, we added detailed description in the revised manuscript, section "Method: Protein expression and purification", as follows:

"*For the purification of the TDP-43 LC construct, TDP-43 LC was overexpressed in BL21(DE3) E. coli cells (TransGen Biotech, CD601-02) by adding 1 mM IPTG and incubating at 37 °C for 12 h. The cells were then harvested and lysed in a buffer containing 50 mM Tris-HCl, pH 8.0, and 100 mM NaCl. Cell pellets were collected by centrifugation (16,000 rpm, 4 °C, 1 h) and subsequently resuspended in a denaturing buffer containing 50 mM Tris-HCl, pH 8.0, 6 M guanidine hydrochloride with sonication. The resuspended protein was filtered and then loaded onto a Ni column (GE Healthcare, USA). The protein was eluted using a denaturing elution buffer containing 50 mM Tris, pH 8.0, 6 M guanidine hydrochloride and 100 mM imidazole, and was further concentrated into over 30 mg/ml proteins, flash-frozen and stored at -80 °C. Before experiments, the protein was desalted using a desalting column (GE Healthcare, USA) into a buffer containing 20 mM MES, pH 6.0. The protein was not concentrated after desalination and ThT experiment was carried out immediately.*"

Minor comments:

. In figure 1, panel A: not clear what means "more CHCl3"

Response: We appreciate the reviewer's suggestion. To address this, we included a table in the revised manuscript, detailing the specific quantities and proportions of $CHCl_3$ and other reagents used in the synthesis process, as shown below:

Table 1. Proportions of Reagents Used in the Synthesis of GQDs.

Sample	1,5-diaminonaphthalene / g	$CHCl_3$/ mL	Ethanol / mL	Catechol / g
GQDs-Cl*	0.1	3	7	0
GQDs-Cl	0.1	1	9	0
Cl-GQDs-OH	0.1	1.5	8.5	0.025
GQDs	0.1	0	10	0

. A figure showing the TDP-43 sequence with the position of the different domains is useful. It is present in the manuscript, but only in Figure 5h. I propose to introduce it earlier in the figures.

Response: We sincerely thank this reviewer for his/her insightful suggestion. We carefully considered the flow and structure of our manuscript, and revised the manuscript to include the TDP-43 domain structure in Figure 2 a and Figure S1a.

. The inhibition of droplet formation (shown in Supporting Figure SI1) seems less efficient compared to what was reported by the same authors for FUS (in the publication: https://doi.org/10.1021/acsnano.3c00001, in figure 2), using the same concentration of 0.1mg/mL. Can the authors comment of this aspect ?

Response: We thank the reviewer very much for the careful reading and the insightful suggestion.

Firstly, it's crucial to recognize the inherent differences between TDP-43 and FUS proteins, as these influence their behavior in LLPS. For instance, TDP-43's LLPS is primarily driven by π-π interactions from its aromatic residues and a transient α-helix structure[14, 26, 27]. Additionally, its arginine and hydrophobic residues are key in modulating its biomolecular condensate properties[26]. In contrast, FUS's LLPS involves hydrogen bonds, π-interactions, and hydrophobic interactions within its LCR, with glutamine residues playing a significant role[28, 29].Moreover, FUS contains SYGQ rich and multiple RGG regions, and these regions contribute to the protein's ability to engage in various molecular interactions[14].Secondly, the difference in the observed efficiency of droplet formation inhibition could be attributed to the fact that the GQDs used in our study are distinct due to the incorporation of chlorine, which alters their electronic properties, chemical reactivity, and surface properties.

In sum, while both studies used the same concentration of 0.1mg/mL, the differences in protein and GQDs characteristics may explain the variance in the efficiency of droplet formation inhibition observed between TDP-43 and FUS.

. Immobilization of TDP-43 for the BLI experiment is not well described.

Response: We apologize that our initial description may not have been sufficiently detailed, and we are grateful for the opportunity to enhance the clarity of our manuscript. Taken the reviewer's suggestion, we revised the "Materials and methods: BLI" section as follows:

"*The binding kinetics were assessed using a ForteBio Octet RED96 system (Pall ForteBio LLC).*

A total volume of 200 μL either sample or buffer (50 mM Tris, pH 7.5, 150 mM NaCl) was dispensed into a 96-well black flat-bottom plate. The plate was incubated at 37 ℃ with orbital shaking set to 1000 rpm. The biosensors were first incubated with GQDs to eliminate the non-specifically binding. Proteins were immobilized onto the Ni-NTA biosensors (Sartorius, 185101) at a concentration of 20 μg/ml. Different concentrations of GQDs were further incubated with biosensors during the association step, followed by a disassociation step. The resulting curves were adjusted using a blank reference and analyzed using the ForteBio Data Analysis software (version 9.0)."

. Please comment if the use of the fused MBP in the TDP-43 construct can also lead to artifacts during the BLI experiments.

Response: We understand the reviewer's concern. Taken the reviewer's suggestion, we performed BLI assay to assess the direct interaction between the TDP-43 protein and halogen atom-doped GQDs, as shown in Figure R3-1 and referenced in reviewer 3's first concern. Additionally, our cellular model experiments demonstrated the interaction of TDP-43 with GQDs in the absence of the MBP tag. Based on these findings, we believe the MBP tag's effect on the interaction between TDP-43 and GQDs is minimal in the BLI experiments.

. The authors show that the LC domain directly interacts with GQDs. Can they rule out that the folded N-terminal domain or the RNA-binding motifs do not directly interact with GQDs ?

Response: We are grateful to the reviewer for raising this interesting question. To fully address the reviewer's question, we conducted additional affinity experiments to investigate the interactions of the NTD, RRM, and LC domains of TDP-43 with halogen atom-doped GQDs with BLI assay, respectively. The results show that halogen atom-doped GQDs bind to TDP-43 NTD, RRM, and LC domains (Figure R3-4 a-f), with the strongest binding observed in the LC domain, possibly due to its structural flexibility.

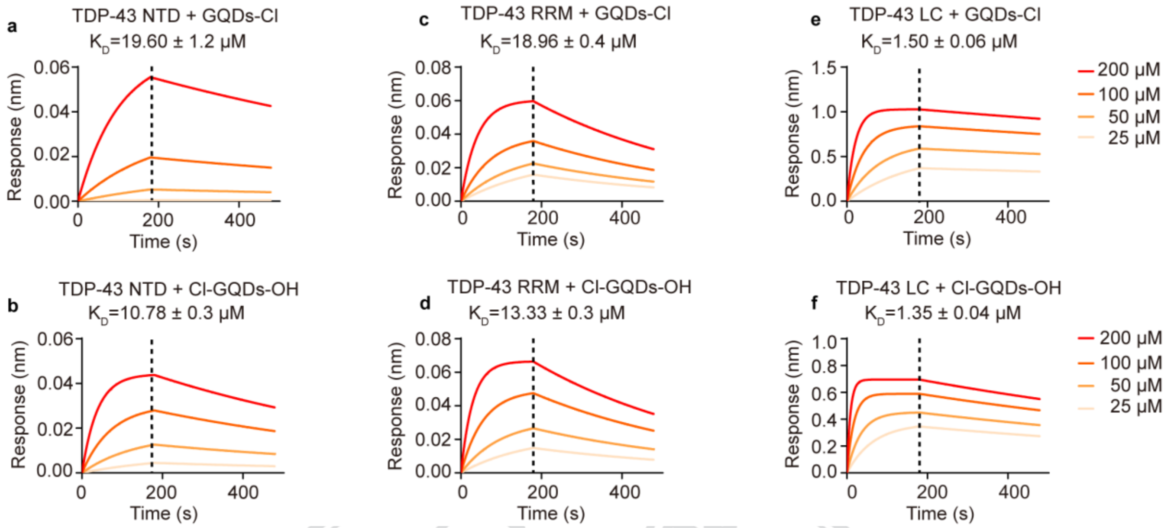

Figure R3-4. **Binding affinity of GQDs to various TDP-43 domains.** (a-f) Kinetic binding curves of TDP-43 NTD, RRM, LC domian with a concentration gradient of GQDs-Cl or Cl-GQDs-OH. The association and dissociation profiles are divided by a vertical black dotted line.

In the revised manuscript, we included Figure R3-4 as Supplementary Figure S12, described in the Results section, "Halogen atom-doped GQDs inhibit TDP-43 LC fibrillation" as follows:

"*TDP-43 features an N-terminal domain with dimerization propensity, two RRM domains with β-α-β motifs, and an LCD domain, mediating liquid-to-solid phase transitions and pathological fibril formation*[37,38]. *BLI assays were used to quantify the binding affinity between halogen atom-doped GQDs and TDP-43 protein domains. The results demonstrate that halogen atom-doped GQD interact with various domains of TDP-43, including the NTD, RRM, and LC domain (Figure S12 a-f). The* K_D *of GQDs-Cl and Cl-GQDs-OH to the TDP-43 NTD was measured as 19.60 μM and 10.78 μM, respectively. These GQDs also exhibit a similar binding affinity to the TDP-43 RRM domain, with* K_D *values of 18.96 μM and 13.33 μM. Notably, for the TDP-43 LC domain, BLI data revealed that halogen atom-doped GQDs bind with* K_D *values of 1.50 μM and 1.35 μM, suggesting the strongest binding affinity among the three domains, potentially attributable to its flexible structure……*"

Reference:

1. Mackenzie, I. R.; Neumann, M.; Baborie, A.; Sampathu, D. M.; Du Plessis, D.; Jaros, E.; Perry, R. H.; Trojanowski, J. Q.; Mann, D. M.; Lee, V. M., A harmonized classification system for FTLD-TDP pathology. *Acta Neuropathol* **2011,** *122* (1), 111-3.
2. Mackenzie, I. R.; Neumann, M., Reappraisal of TDP-43 pathology in FTLD-U subtypes. *Acta*

Neuropathol **2017,** *134* (1), 79-96.

3. Neumann, M.; Mackenzie, I. R.; Cairns, N. J.; Boyer, P. J.; Markesbery, W. R.; Smith, C. D.; Taylor, J. P.; Kretzschmar, H. A.; Kimonis, V. E.; Forman, M. S., TDP-43 in the ubiquitin pathology of frontotemporal dementia with VCP gene mutations. *J Neuropathol Exp Neurol* **2007,** *66* (2), 152-7.

4. Tome, S. O.; Vandenberghe, R.; Ospitalieri, S.; Van Schoor, E.; Tousseyn, T.; Otto, M.; von Arnim, C. A. F.; Thal, D. R., Distinct molecular patterns of TDP-43 pathology in Alzheimer's disease: relationship with clinical phenotypes. *Acta Neuropathol Commun* **2020,** *8* (1), 61.

5. Gu, J.; Wang, C.; Hu, R.; Li, Y.; Zhang, S.; Sun, Y.; Wang, Q.; Li, D.; Fang, Y.; Liu, C., Hsp70 chaperones TDP-43 in dynamic, liquid-like phase and prevents it from amyloid aggregation. *Cell Res* **2021,** *31* (9), 1024-1027.

6. Spence, H.; Waldron, F. M.; Saleeb, R. S.; Brown, A.-L.; Rifai, O. M.; Gilodi, M.; Read, F.; Roberts, K.; Milne, G.; Wilkinson, D.; O'Shaughnessy, J.; Pastore, A.; Fratta, P.; Shneider, N.; Tartaglia, G. G.; Zacco, E.; Horrocks, M. H.; Gregory, J. M., RNA aptamer reveals nuclear TDP-43 pathology is an early aggregation event that coincides with STMN-2 cryptic splicing and precedes clinical manifestation in ALS. **2023**.

7. Udan-Johns, M.; Bengoechea, R.; Bell, S.; Shao, J.; Diamond, M. I.; True, H. L.; Weihl, C. C.; Baloh, R. H., Prion-like nuclear aggregation of TDP-43 during heat shock is regulated by HSP40/70 chaperones. *Hum Mol Genet* **2014,** *23* (1), 157-70.

8. Gu, J., Wang, C., Hu, R., Li, Y., Zhang, S., Sun, Y., Wang, Q., Li, D., Fang, Y., Liu, C., Hsp70 chaperones TDP-43 in dynamic, liquid-like phase and prevents it from amyloid aggregation. *Cell Res.* **2021,** *31* (9), 1024-1027.

9. Wang, C.; Duan, Y. J.; Duan, G.; Wang, Q. Q.; Zhang, K.; Deng, X.; Qian, B. T.; Gu, J. G.; Ma, Z. W.; Zhang, S.; Guo, L.; Liu, C.; Fang, Y. S., Stress Induces Dynamic, Cytotoxicity-Antagonizing TDP-43 Nuclear Bodies via Paraspeckle LncRNA -Mediated Liquid-Liquid Phase Separation. *Molecular Cell* **2020,** *79* (3), 443-+.

10. Yu, H., Lu, S., Gasior, K., Singh, D., Vazquez-Sanchez, S., Tapia, O., Toprani, D., Beccari, M. S., Yates, J. R., Da Cruz, S., Newby, J. M., Lafarga, M., Gladfelter, A. S., Villa, E., Cleveland, D. W., HSP70 chaperones RNA-free TDP-43 into anisotropic intranuclear liquid spherical shells. *Science* **2021,** *371* (6529), eabb4309.

11. Inukai, Y.; Nonaka, T.; Arai, T.; Yoshida, M.; Hashizume, Y.; Beach, T. G.; Buratti, E.; Baralle, F. E.; Akiyama, H.; Hisanaga, S.; Hasegawa, M., Abnormal phosphorylation of Ser409/410 of TDP-43 in FTLD-U and ALS. *FEBS Lett* **2008,** *582* (19), 2899-904.

12. Neumann, M.; Kwong, L. K.; Lee, E. B.; Kremmer, E.; Flatley, A.; Xu, Y.; Forman, M. S.; Troost, D.; Kretzschmar, H. A.; Trojanowski, J. Q.; Lee, V. M., Phosphorylation of S409/410 of TDP-43 is a consistent feature in all sporadic and familial forms of TDP-43 proteinopathies. *Acta Neuropathol* **2009,** *117* (2), 137-49.

13. Chen, H. J.; Topp, S. D.; Hui, H. S.; Zacco, E.; Katarya, M.; McLoughlin, C.; King, A.; Smith, B. N.; Troakes, C.; Pastore, A.; Shaw, C. E., RRM adjacent TARDBP mutations disrupt RNA binding and enhance TDP-43 proteinopathy. *Brain* **2019,** *142* (12), 3753-3770.

14. Zbinden, A.; Perez-Berlanga, M.; De Rossi, P.; Polymenidou, M., Phase Separation and Neurodegenerative Diseases: A Disturbance in the Force. *Dev Cell* **2020,** *55* (1), 45-68.

15. Alberti, S.; Hyman, A. A., Biomolecular condensates at the nexus of cellular stress, protein aggregation disease and ageing. *Nat Rev Mol Cell Biol* **2021,** *22* (3), 196-213.

16. Atukorale, P. U.; Guven, Z. P.; Bekdemir, A.; Carney, R. P.; Van Lehn, R. C.; Yun, D. S.; Jacob Silva, P. H.; Demurtas, D.; Yang, Y. S.; Alexander-Katz, A.; Stellacci, F.; Irvine, D. J., Structure-Property Relationships of Amphiphilic Nanoparticles That Penetrate or Fuse Lipid Membranes. *Bioconjug Chem* **2018,** *29* (4), 1131-1140.

17. Lin, X.; Lin, X., Designing amphiphilic Janus nanoparticles with tunable lipid raft affinity via molecular dynamics simulation. *Biomater Sci* **2021,** *9* (24), 8249-8258.

18. Gerebtzoff, G.; Li-Blatter, X.; Fischer, H.; Frentzel, A.; Seelig, A., Halogenation of drugs enhances membrane binding and permeation. *Chembiochem* **2004,** *5* (5), 676-84.

19. Zhang, H.; Gu, J. E.; Zhang, Y. L.; Guo, H. Z.; Zhang, S. N.; Song, J.; Liu, C.; Wang, L.; Li, D.; Dai, B., Graphene Quantum Dots Modulate Stress Granule Assembly and Prevent Abnormal Phase Transition of Fused in Sarcoma Protein. *Acs Nano* **2023,** *17* (11), 10129-10141.

20. Zheng, X. T.; Ananthanarayanan, A.; Luo, K. Q.; Chen, P., Glowing graphene quantum dots and

carbon dots: properties, syntheses, and biological applications. *Small* **2015,** *11* (14), 1620-36.

21. Yan, Y.; Gong, J.; Chen, J.; Zeng, Z.; Huang, W.; Pu, K.; Liu, J.; Chen, P., Recent Advances on Graphene Quantum Dots: From Chemistry and Physics to Applications. *Adv Mater* **2019,** *31* (21), e1808283.

22. Nurunnabi, M.; Khatun, Z.; Huh, K. M.; Park, S. Y.; Lee, D. Y.; Cho, K. J.; Lee, Y. K., In vivo biodistribution and toxicology of carboxylated graphene quantum dots. *ACS Nano* **2013,** *7* (8), 6858-67.

23. Chong, Y.; Ma, Y.; Shen, H.; Tu, X.; Zhou, X.; Xu, J.; Dai, J.; Fan, S.; Zhang, Z., The in vitro and in vivo toxicity of graphene quantum dots. *Biomaterials* **2014,** *35* (19), 5041-8.

24. Yang, K.; Wan, J.; Zhang, S.; Zhang, Y.; Lee, S. T.; Liu, Z., In vivo pharmacokinetics, long-term biodistribution, and toxicology of PEGylated graphene in mice. *ACS Nano* **2011,** *5* (1), 516-22.

25. Babinchak, W. M.; Haider, R.; Dumm, B. K.; Sarkar, P.; Surewicz, K.; Choi, J. K.; Surewicz, W. K., The role of liquid-liquid phase separation in aggregation of the TDP-43 low-complexity domain. *J Biol Chem* **2019,** *294* (16), 6306-6317.

26. Schmidt, H. B.; Barreau, A.; Rohatgi, R., Phase separation-deficient TDP43 remains functional in splicing. *Nat Commun* **2019,** *10* (1), 4890.

27. Conicella, A. E.; Zerze, G. H.; Mittal, J.; Fawzi, N. L., ALS Mutations Disrupt Phase Separation Mediated by alpha-Helical Structure in the TDP-43 Low-Complexity C-Terminal Domain. *Structure* **2016,** *24* (9), 1537-49.

28. Murthy, A. C.; Dignon, G. L.; Kan, Y.; Zerze, G. H.; Parekh, S. H.; Mittal, J.; Fawzi, N. L., Molecular interactions underlying liquid-liquid phase separation of the FUS low-complexity domain. *Nat Struct Mol Biol* **2019,** *26* (7), 637-648.

29. Qamar, S.; Wang, G.; Randle, S. J.; Ruggeri, F. S.; Varela, J. A.; Lin, J. Q.; Phillips, E. C.; Miyashita, A.; Williams, D.; Strohl, F.; Meadows, W.; Ferry, R.; Dardov, V. J.; Tartaglia, G. G.; Farrer, L. A.; Kaminski Schierle, G. S.; Kaminski, C. F.; Holt, C. E.; Fraser, P. E.; Schmitt-Ulms, G.; Klenerman, D.; Knowles, T.; Vendruscolo, M.; St George-Hyslop, P., FUS Phase Separation Is Modulated by a Molecular Chaperone and Methylation of Arginine Cation-pi Interactions. *Cell* **2018,** *173* (3), 720-734 e15.

Reviewers' Comments:

Reviewer #1:
Remarks to the Author:
The authors attempted to address my comments however the biology validation part of the study was not expanded as requested and remains rather weak, I am afraid. A major issue is the K181E mutant data. These data (Figure 5) are inconsistent and confusing. RFP-tagged mutant was used for the first time, and no WT TDP-43 data were provided for this tag. Images in C in fact do not show aggregates, the signal looks more like diffuse "territories", usually 1-2 massive blobs per cell, which was not addressed. Further, quantification in L,M shows that there are ~15-20 aggregates in PBS condition. How is this possible? No "aggregation-free" (e.g. WT condition) is shown for comparison. Furthermore, After GQD treatment, the number of RFP-positive cells in the field is reduced (one per field, instead of 3-4 in PBS), and these cells have an even more granulated pattern. According to WB, protein levels do not change, so the number of positive cells should be the same but with more diffuse pattern which is not the case. Most confusingly, images are for RFP whereas WBs are for GFP.
Figure S5 – Image in A for the mutant is not convincing and probably corresponds to a dying cell or contains an imaging artefact: the is an empty space in the middle which looks odd and cannot correspond to any cell structure. The authors should provide a range of images (at least 10) of healthy cells for this phenotype used for FRAP.
Overall, further biology validation, with proper controls, more convincing phenotypes and more in-depth analysis of TDP-43 condensation in cellular systems, is required.

Secondly, the authors state that:
". Thus, for potential therapeutic application, it will be important toapply GQDs to the cells with solidified TDP-43 condensates and aggregatesrather than to those with dynamic TDP-43 NBs, in orderto diminish the potential side-effect GQDs in disruptingthe functional TDP-43 NBs in normal cells."
How do they see this achieved technically? How would this selective targeting be possible? I am afraid that GQDs will affect all condensate types, including neuroprotective ones, and are therefore an unlikely therapeutic choice.
Minor:
Figure S4 – this control panel should be in the main figure, to be able to compare with the treated cells.
Fig S2 – many image labels are misaligned
Fig2a – TDP-43 schematic is odd, why both N-terminal and C-terminal parts are labelled LCD and why is single RRM shown? In Fig5, the structure is shown differently.
Panel arrangement in Fig 2 is also highly irregular – b comes before a, never seen this kind of arrangement before.
Abstract – 'decrease amyloid clumping' – unusual way to describe TDP-43 condensation, needs revision. Similarly, "irregular aggregation" is confusing.

Reviewer #2:
Remarks to the Author:
I recommend this revised manuscript for publication as is.

Reviewer #3:
Remarks to the Author:
The authors have addressed all of my concerns.

Point-by-point response to the reviewers' comments:

We thank the reviewers for the valuable comments and suggestions to our work. We have performed additional experiments, added new data, figures and carefully revised the text as the reviewers suggested. All comments are addressed point-by-point below:

*Reviewers' comments are in *Italic*. Our responses are in blue. All revisions are highlighted in red in the revised manuscripts.

Reviewer#1:

Comments:

**The authors attempted to address my comments however the biology validation part of the study was not expanded as requested and remains rather weak, I am afraid. A major issue is the K181E mutant data. These data (Figure 5) are inconsistent and confusing.*

Response: We would like to express our gratitude for the insightful comments provided by the reviewer. We sincerely apologize for any confusion that may have arisen and are grateful for this opportunity to offer further clarification. In response, we have taken the following actions: 1. Thoroughly reexamined all cell experiments, ensuring rigorous validation and the inclusion of appropriate control groups. 2. Provided a detailed explanation for Figure 5 and supplemented it with additional data. We hope these revisions and clarifications will effectively address the concerns raised.

RFP-tagged mutant was used for the first time, and no WT TDP-43 data were provided for this tag.

Response: We are immensely grateful to the reviewer for his/her insightful and constructive comments. In response to the reviewer's suggestion, we added results using the plasmid TDP-43 RFP in Figure R1a-d. These results demonstrate that under non-stress conditions, HeLa cells expressing TDP-43 RFP plasmids exhibit normal fusion of TDP-43 without any aggregation.

Additionally, we have re-examined our results and supplemented the data with TDP-43 GFP in Figure R1e-h.

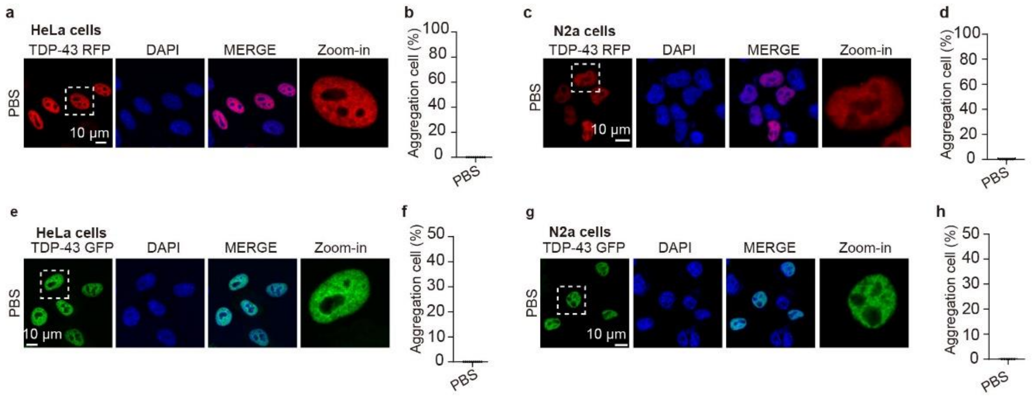

Figure R1. Cellular expression of TDP-43 RFP/GFP plasmid demonstrates protein dispersion. (a, c, e, g) Fluorescence images of HeLa or N2a cells transfected with the TDP-43 RFP/GFP plasmid. Scale bar: 10 μm. (b, d, f, h) Quantitative analysis of the number of aggregates for image a, c, e, g.

In the revised manuscript, we included Figure R1 as Figure 5c-f, j-m.

Images in C in fact do not show aggregates, the signal looks more like diffuse "territories", usually 1-2 massive blobs per cell, which was not addressed.

Response: We thank the reviewer for bringing this to our attention and sincerely apologize for any confusion caused by our initial imaging settings. The original exposure conditions were optimized to highlight protein aggregates, which inadvertently reduced the visibility of cells with dispersed protein distribution due to the strong fluorescent intensity from the aggregates. To address this oversight, we conducted additional imaging under varied exposure conditions at the same position (Figure R2a). These updated images not only clarify the overexposed signals indicative of aggregation but also reveal a considerable number of cells exhibiting protein dispersion, which is morphologically distinct from the aggregates.

Furthermore, we observed bright, irregularly shaped aggregates co-localizing with pTDP-43 (S409/410) antibodies (highlighted by yellow arrows), in contrast to cells with dispersed protein, which lack phosphorylation at S409/410 (denoted by write arrows) in Figure R2b. Additionally, cells displaying these aggregates did not exhibit recovery post-FRAP (Figure R2c-d), affirming their as aggregated cells. These findings are consistent with those from previous studies[1, 2].

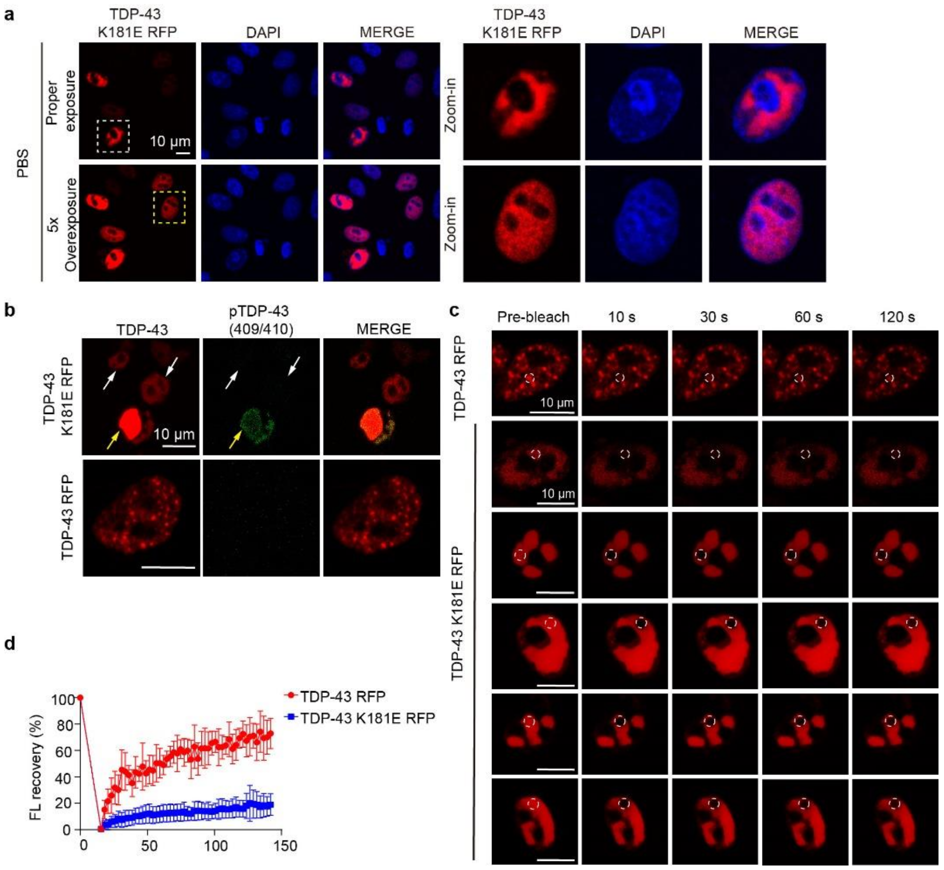

Figure R2. Protein aggregation and dispersion within the nucleus. (a) Fluorescence microscopy images showing HeLa cells transfected with TDP-43 K181E RFP observed under proper exposure and 5x enhanced exposure conditions. The white dotted box indicates cells with protein aggregation, while the yellow dotted box shows cells with protein dispersion. The scale bar is 10 μm. (b) Immunofluorescence images of cells transfected with the respective plasmids, stained for phosphorylated TDP-43 (S409/410). Irregularly shaped aggregates co-localizing with pTDP-43 (S409/410) antibodies (highlighted by yellow arrows), in contrast to cells with dispersed protein, which lack phosphorylation at S409/410 (denoted by write arrows). (c) Representative fluorescence microscopy images showing TDP-43 RFP nuclear bodies and TDP-43 K181E RFP nuclear aggregates, with regions (dashed circles) subjected to photobleaching in the FRAP assay. (d) Fluorescence intensity recovery curves are displayed.

In the revised manuscript, we included Figure R2a as Supplementary Figure S12a, and described it in the Results section: "Halogen atom-doped GQDs can inhibit aggregation of TDP-43 and decrease pTDP-43 (S409/410) level", as follows:

"*Furthermore, both GQDs-Cl and Cl-GQDs-OH were observed to increase the number of cells with dispersed protein (Figure S12a-c).*"

Additionally, we included Figure R2b-d as Supplementary Figure S4, and described them in the Results section: "GQDs-NH_2 and GQDs-OH inhibit TDP-43 condensation in the cytoplasm but

not in the nucleus", as follows:

"*The TDP-43 K181E mutation, associated with ALS/FTD*[26]*, triggered the spontaneous formation of large, irregularly shaped TDP-43 nuclear aggregates, even in the absence of stress conditions*[27]*. Nuclear aggregation of TDP-43 K181E exhibits slower recovery in the FRAP assay (Figure S4 a-b) and is hyperphosphorylated at S409/410 (Figure S4 c). Although severe protein aggregation occurs, Caspase 3 staining (Figure S4 d) and TUNEL assay (Figure S4 e) demonstrate that cells expressing the TDP-43 K181E plasmid do not undergo apoptosis.*"

In a similar vein, analysis of the TDP-43 CTF truncation revealed that protein aggregation led to intense fluorescence, predominantly highlighting aggregated cells in Figure 5. To counter this, we conducted further imaging of TDP-43 CTF truncation under varied exposure levels, revealing that cells with dispersed protein distributions are clearly distinguishable from those with aggregation (Figure R3a).

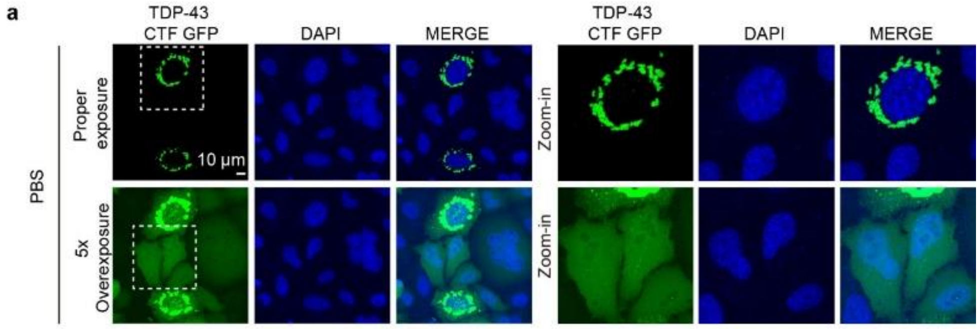

Figure R3. Protein aggregation and dispersion within the cytoplasm of HeLa Cells. (a) Fluorescence microscopy images of HeLa cells transfected with TDP-43 CTF GFP and treated with either PBS or 0.02 μg/μL GQDs for 16 hours, under proper and 5x exposure conditions. Scale bar represents 10 μm.

In the revised manuscript, we included Figure R3a as Supplementary Figure S13a, and described it in the Results section: "Halogen atom-doped GQDs can inhibit aggregation of TDP-43 and decrease pTDP-43 (S409/410) level", as follows:

"*This treatment significantly reduced the number of cells with cytoplasmic aggregates while increasing those exhibiting cytoplasmic dispersion, as observed with both types of GQDs (Figure 5 i-m, S13 a-c)*"

Further, quantification in L,M shows that there are ~15-20 aggregates in PBS condition. How is this possible? No "aggregation-free" (e.g. WT condition) is shown for comparison.

Response: We are grateful for the reviewer's insightful comments. In response, we added results depicting cells in an "aggregation-free" state using the TDP-43 GFP plasmid (Figure R4 a-d). These results reveal that, in the absence of external stimuli, cells expressing TDP-43 GFP displays protein dispersion, highlighting the natural state of protein distribution in the nucleus. In contrast, cells overexpressing the mutant TDP-43 CTF spontaneously form aggregate in the cytoplasm, even without external stimuli. This observation is consistent with previous research[3, 4], which has established that TDP-43 CTF is prone to form ubiquitinated, phosphorylated (S409/410), and insoluble cytoplasmic inclusions without any external stimuli, underscoring the pathological role of the TDP-43 CTF mutation in ALS.

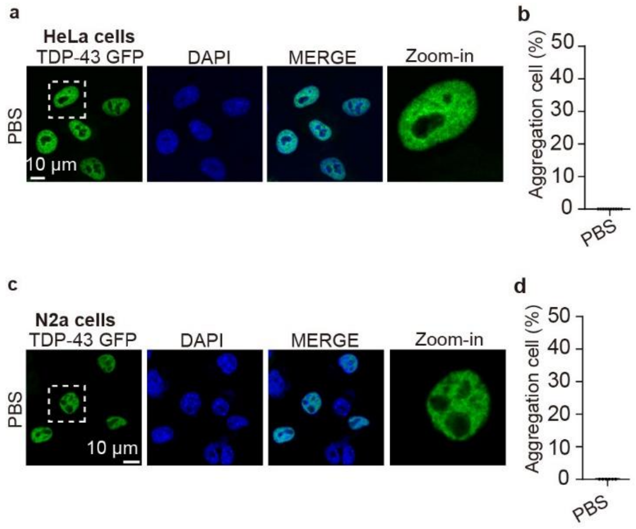

Figure R4. Cellular expression TDP-43 GFP plasmid demonstrates protein dispersion. (a, c) Fluorescence images of HeLa or N2a cells transfected with TDP-43 GFP plasmid. Scale bar: 10 μm. (b, d) Quantitative analysis of the number of aggregates for image a and c.

In the revised manuscript, we included Figure R4 as Figure 5j,k,l,m.

Furthermore, After GQD treatment, the number of RFP-positive cells in the field is reduced (one per field, instead of 3-4 in PBS), and these cells have an even more granulated pattern. According to WB, protein levels do not change, so the number of positive cells should be the same but with more diffuse pattern which is not the case.

Response: We are grateful to the reviewer for raising this important point. The issue primarily arises

from the fact that cells with aggregated proteins tend to exhibit extremely bright fluorescence, which can overshadow the visibility of cells with dispersed proteins. As a result, the RFP-positive cells shown in Figure 5c are primarily those with protein aggregates. The application of GQDs results in a reduction in the number of aggregated cells, alongside an increase in cells exhibiting protein dispersion. Due to the limitations inherent in photographic exposure, it is challenging to simultaneously represent both aggregated and dispersed cells in a single image adequately. We supplemented the Figure R5, conducted additional imaging under varied exposure conditions at the same position. The results show that after GQDs treatment, there is a noticeable decrease in cells with aggregation and a corresponding increase in cells with dispersed proteins. WB results showing unchanged protein levels, these results account for total protein levels from both aggregated and dispersed proteins.

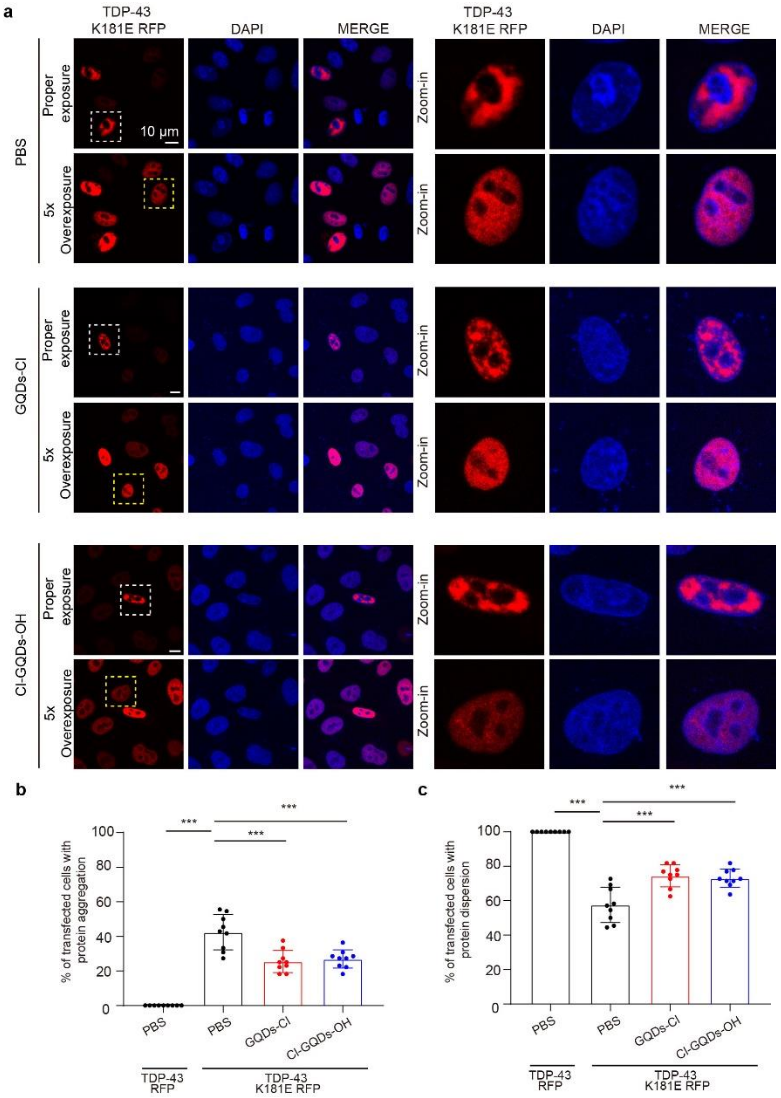

Figure R5. Impact of GQDs-Cl and Cl-GQDs-OH on protein aggregation and dispersion in the nucleus of HeLa cells. (a) Fluorescence microscopy images of HeLa cells transfected with

TDP-43 K181E RFP and treated with either PBS or 0.02 μg/μL GQDs for 16 hours, under proper and 5x exposure conditions. The white dotted box indicates cells with protein aggregation, while the yellow dotted box shows cells with protein dispersion. The scale bar represents 10 μm. (b) Quantitative analysis of the number of cells displaying aggregates for images a. Data correspond to the mean ± SD, n =9. Student's *t* test, ***P < 0.001. (c) Quantitative analysis of the number of transfected cells exhibiting protein dispersion for image a. Data correspond to the mean ± SD, n =9. Student's *t* test, ***P < 0.001.

In the revised manuscript, we included Figure R5 as Supplementary Figure S12, and described it in the Results section: "Halogen atom-doped GQDs can inhibit aggregation of TDP-43 and decrease pTDP-43 (S409/410) level", as follows:

"*Furthermore, both GQDs-Cl and Cl-GQDs-OH were observed to increase the number of cells with dispersed protein (Figure S12 a-c).*"

Most confusingly, images are for RFP whereas WBs are for GFP.

Response: We sincerely apologize for the inconsistency and are deeply grateful to the reviewer for his/her meticulous attention to detail. We conducted a new set of experiments to ensure that the fluorescence-labeled plasmid used in the WB experiments matches the one used in the immunofluorescent staining (Figure R6 a-b). In the revised manuscript, we included Figure R6 as Figure 5 g,h.

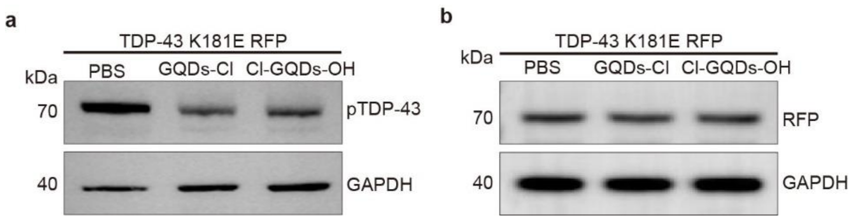

Figure R6. Analysis of total TDP-43 protein and pTDP-43 (S409/410) expression levels via WB assay. (a) Western blot assay displaying the expression levels of pTDP-43 (S409/410) in transfected cells. GAPDH serves as a loading control. (b) Western blot analysis showing the expression levels of TDP-43 K181E RFP in transfected cells, with GAPDH used as the loading control.

Figure S5 – Image in A for the mutant is not convincing and probably corresponds to a dying cell or contains an imaging artefact: the is an empty space in the middle which looks odd and cannot

correspond to any cell structure. The authors should provide a range of images (at least 10) of healthy cells for this phenotype used for FRAP.

Overall, further biology validation, with proper controls, more convincing phenotypes and more in-depth analysis of TDP-43 condensation in cellular systems, is required.

Response: We appreciate the reviewer's insightful feedback. In response to the concerns about the image in Figure S5a, we included FRAP images of an additional 10 cells to show the morphologies observed in protein aggregation following the overexpression of TDP-43 K181E. The diverse morphologies of protein aggregation that occur following the overexpression of TDP-43 K181E can indeed present varied appearances. This observation is also consistent with previous studies[1, 2].

To ascertain that the cells exhibiting these varied aggregation morphologies are not undergoing apoptosis, we performed a TUNEL assay (Figure R7 e). This assay, in conjunction with our earlier results from the Caspase 3 staining (Figure R7 d), conclusively shows that the cells in question are not apoptotic.

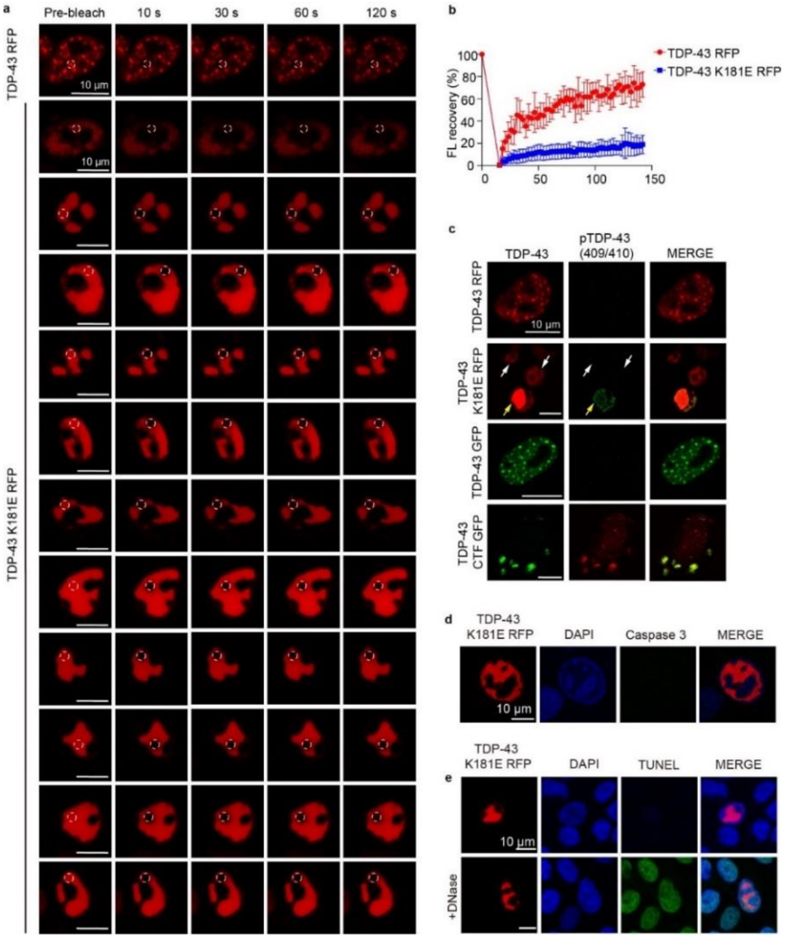

Figure R7. Aggregation of TDP-43 K181E in the nucleus. (a) Representative fluorescence microscopy images showing TDP-43 RFP nuclear bodies and TDP-43 K181E RFP nuclear aggregates, with regions (dashed circles) subjected to photobleaching in the FRAP assay. (b) Fluorescence intensity recovery curves are displayed, with TDP-43 RFP n=5 and TDP-43 K181E RFP n=11. (c) Immunofluorescence images of cells transfected with the respective plasmids, stained for phosphorylated TDP-43 (S409/410). (d) Images of HeLa cells expressing TDP-43 K181E RFP, immunostained for Caspase-3 to assess apoptosis. (e) TUNEL assay on HeLa cells expressing TDP-43 K181E RFP, with DNase treatment serving as a positive control.

And we included Figure R7 as Supplementary Figure S4, and described it in the Results section: "GQDs-NH_2 and GQDs-OH inhibit TDP-43 condensation in the cytoplasm but not in the nucleus", as follows:

"*The TDP-43 K181E mutation, associated with ALS/FTD*[26]*, triggered the spontaneous formation of large, irregularly shaped TDP-43 nuclear aggregates, even in the absence of stress conditions*[27]*. Nuclear aggregation of TDP-43 K181E exhibits slower recovery in the FRAP assay (Figure S4 a-b) and is hyperphosphorylated at S409/410 (Figure S4 c). Although severe protein aggregation occurs, Caspase 3 staining (Figure S4 d) and TUNEL assay (Figure S4 e) demonstrate that cells expressing the TDP-43 K181E plasmid do not undergo apoptosis.*"

Secondly, the authors state that:

". Thus, for potential therapeutic application, it will be important to apply GQDs to the cells with solidified TDP-43 condensates and aggregates rather than to those with dynamic TDP-43 NBs, in order to diminish the potential side-effect GQDs in disrupting the functional TDP-43 NBs in normal cells." How do they see this achieved technically? How would this selective targeting be possible? I am afraid that GQDs will affect all condensate types, including neuroprotective ones, and are therefore an unlikely therapeutic choice.

Response: We thank the reviewer for their insightful comments. We agree on the challenges related to the selective targeting capabilities of GQDs and acknowledge the difficulty in distinguishing between pathological and neuroprotective TDP-43 condensates, which currently limits our approach. However, the synthesis flexibility of GQDs offers a unique opportunity to customize their physicochemical properties, including size, surface functionalization, biocompatibility, and blood-brain barrier permeability. We propose further exploration into the functionalization of GQDs with

molecules such as amyloid tracers or DNA aptamers, known for their selective affinity towards pathological aggregates. This approach could enhance the specificity of GQDs towards pathogenic TDP-43, offering a promising avenue for the development of targeted therapies for neurological conditions associated with protein aggregation. And we hope to contribute to the development of novel approaches for the treatment of neurological disorders associated with abnormal protein aggregation.

Minor:

Figure S4 – this control panel should be in the main figure, to be able to compare with the treated cells.

Response: We thank the reviewer for raising this point. Following the reviewer's suggestion, we moved the previous Figure S4 to Figure 3 c,d,g,h. Similarly, we move previous Figure S3c (TDP-43 D169G control panel) to Figure 3k,l, as follows:

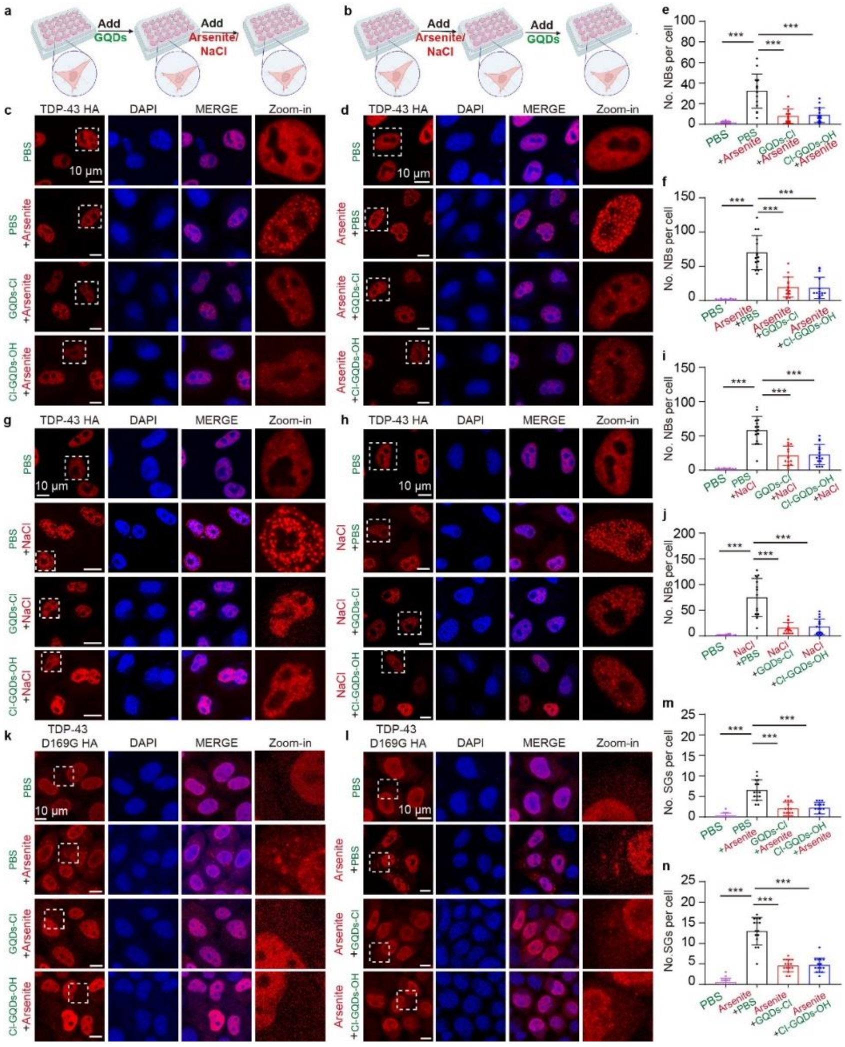

Figure 3. Halogen atom-doped GQDs disassemble preformed TDP-43 granules as well as prevent their formation in the nucleus and cytoplasm. (a) Schematic diagram of the assay for GQDs inhibition of LLPS of TDP-43. (b) Schematic diagram of the TDP-43 granules disassembly assay. (c) Representative confocal images of HeLa cells transfected with TDP-43 HA. Cells were treated with 0.1 μg μL^{-1} of GQDs or PBS (control) for 1 h and then treated with 250 μM arsenite for 1 h. Scale bar, 10 μm. (d) Representative confocal images of HeLa cells transfected with TDP-43 HA. Cells were treated with 250 μM sodium arsenite for 1 h and then treated with 0.1 μg μL^{-1} of both types of GQDs or PBS (control) for 1 h. Scale bar, 10 μm. (e-f) Quantitative analysis of the number of NBs in nucleus per cell for images c and d. Data correspond to the mean ± SD, n = 15. Student's t test, ***P < 0.001. (g) Representative confocal images of HeLa cells transfected with TDP-43 HA. Cells were treated with 0.1 μg μL^{-1} of GQDs or PBS (control) for 1 h and then treated with 300 mM NaCl for 1 h. Scale bar, 10 μm. (h) Representative confocal images of HeLa cells transfected with TDP-43 HA. Cells were treated with 300 mM NaCl for 1 h and then treated with 0.1 μg μL^{-1} of both types of GQDs or PBS (control) for 1 h. Scale bar, 10 μm. (i-j) Quantitative analysis of the number of NBs in nucleus per cell for image g-h. Data correspond to the mean ± SD, n = 15. Student's t test, ***P < 0.001. (k) Representative confocal images of HeLa cells transfected with TDP-43 D169G HA. Cells were treated with 0.1 μg μL^{-1} of GQDs or PBS (control) for 1 h and then treated with 250 μM arsenite for 1 h. Scale bar, 10 μm. (l) Representative confocal images of HeLa cells transfected with TDP-43 D169G HA. Cells were treated with 250 μM sodium arsenite for 1 h and then treated with 0.1 μg μL^{-1} of both types of GQDs or PBS (control) for 1 h. Scale bar, 10 μm. (m-n) Quantitative analysis of the number of SGs in cytoplasm per cell for image k-l. Data correspond to the mean ± SD, n = 15. Student's t test, ***P < 0.001.

Fig S2 – many image labels are misaligned

Response: We appreciate reviewer's careful scrutiny, which helps in maintaining the quality and accuracy of our publication. We carefully reviewed Fig S2 and corrected the misalignments of the image labels as follows:

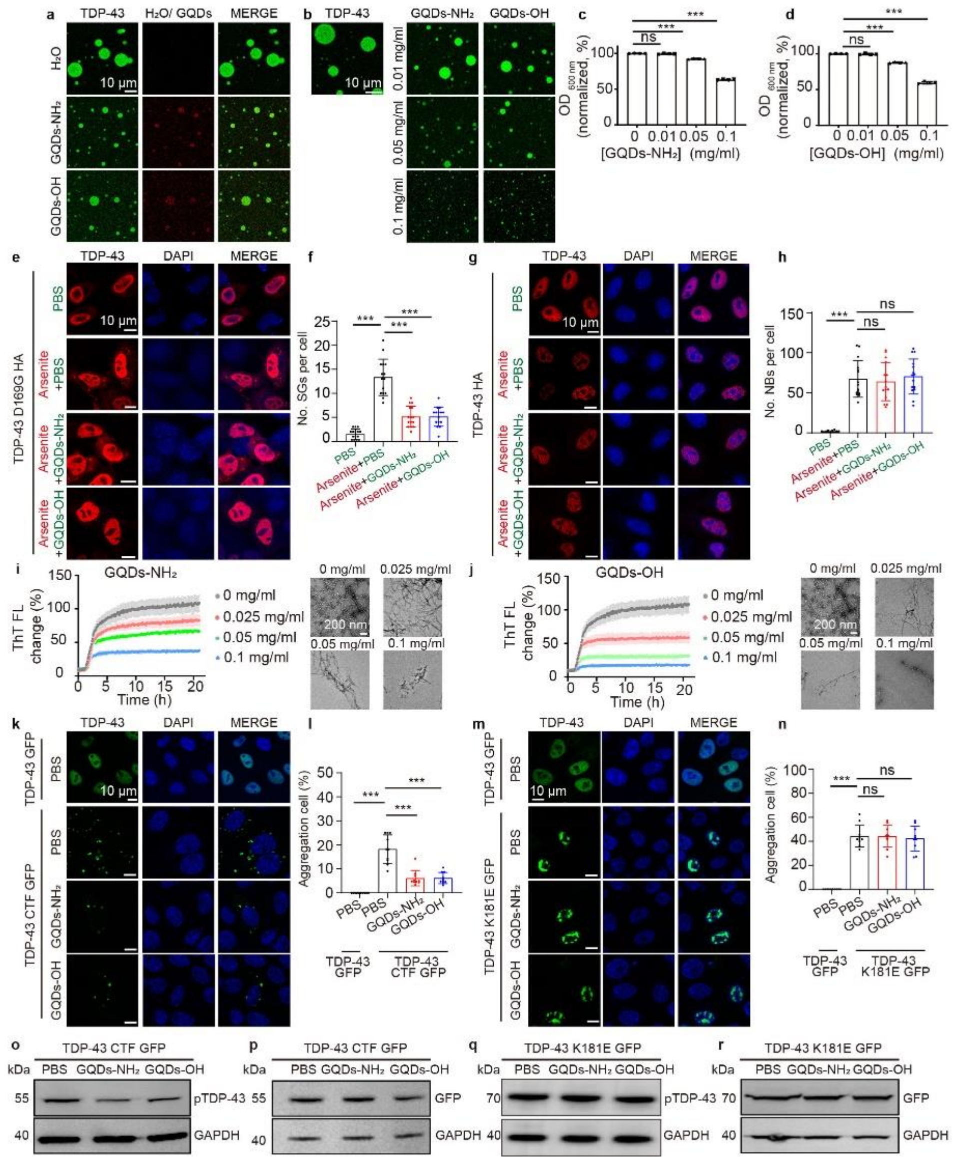

Figure S2. GQDs-NH_2 and GQDs-OH inhibit the TDP-43 phase separation *in vitro*. (a) Fluorescent images of the TDP-43-MBP in the presence and absence of GQDs. 40 μM TDP-43 MBP undergo LLPS in the buffer containing 50 mM Tris, pH 7.5, and 150 mM NaCl, 10% Dextran 70 and then mixed with 0.01 μg μL^{-1} GQDs-NH_2 or GQDs-OH. Scale bar, 10 μm. (b) Representative fluorescence images of 40 μM TDP-43 MBP undergoing phase separation in the presence of different concentrations of GQDs-NH_2 or GQDs-OH. Scale bar, 10 μm. (c-d) Turbidity measurement of the TDP-43 MBP phase separation in the presence of GQDs-NH_2 or GQDs-OH. Data correspond to the mean ± SD, n = 4. Student's t test. ***P < 0.001; ns: not significant. (e) Representative confocal images of HeLa cells transfected with TDP-43 D169G HA. Cells were treated with 250 μM sodium arsenite for 1h and then treated with 0.1 μg μL^{-1} GQDs-NH_2 or GQDs-OH or PBS (control) for 1 h. Scale bar, 10 μm. (f) Quantitative analysis of number of SGs in cytoplasm per cell for image e. Data correspond to the mean ± SD, n = 15. Student's t test, ***P < 0.001; ns: not significant. (g) Representative confocal images of HeLa cells transfected with TDP-43 HA. Cells were treated with 250 μM sodium arsenite for 1h and then treated with 0.1 μg μL^{-1} GQDs-NH_2 or GQDs-OH or PBS (control) for 1 h. Scale bar, 10 μm. (h) Quantitative analysis of the number of NBs in nucleus per cell for image g. Data correspond to the mean ± SD, n = 15. Student's t test, ***P < 0.001; ns: not significant. (i-j) ThT fluorescence assay of 20 μM TDP-43

LC monomers fibrillation in the presence of different concentrations of GQDs-NH_2 or GQDs-OH. Data correspond to the mean ± SD, n = 3. TEM images of the ThT samples in the presence and absence of GQDs at 20 h are shown right. Scale bar, 200 nm. (k) Fluorescence images of HeLa cells transfected with TDP-43 CTF GFP. Cells were treated with PBS or 0.02 μg μL^{-1} GQDs-NH_2 or GQDs-OH for 16 h. Scale bar, 10 μm. (l) Quantitative analysis of the number of aggregation cells for image k. Data correspond to the mean ± SD, n =10. Student's t test, ***$P < 0.001$; ns: not significant. (m) Fluorescence images of HeLa cells transfected with TDP-43 K181E GFP. Cells were treated with PBS or 0.02 μg μL^{-1} GQDs-NH_2 or GQDs-OH for 16 h. Scale bar, 10 μm. (n) Quantitative analysis of the number of aggregation cells for image m. Data correspond to the mean ± SD, n =10. Student's t test, ***$P < 0.001$; ns: not significant. (o-r) Western blot for the expression of pTDP-43 or GFP in the transfected 293T cells. GAPDH serves as a loading control.

Fig2a – TDP-43 schematic is odd, why both N-terminal and C-terminal parts are labelled LCD and why is single RRM shown? In Fig5, the structure is shown differently.

Response: We appreciate the reviewer's attention to detail. In response, we corrected our previous errors and revised the schematic representation to ensure it consistently reflects the composition of the TDP-43 protein.

Panel arrangement in Fig 2 is also highly irregular – b comes before a, never seen this kind of arrangement before.

Response: We thank the reviewer for his/her feedback. We revised Fig 2 as follows:

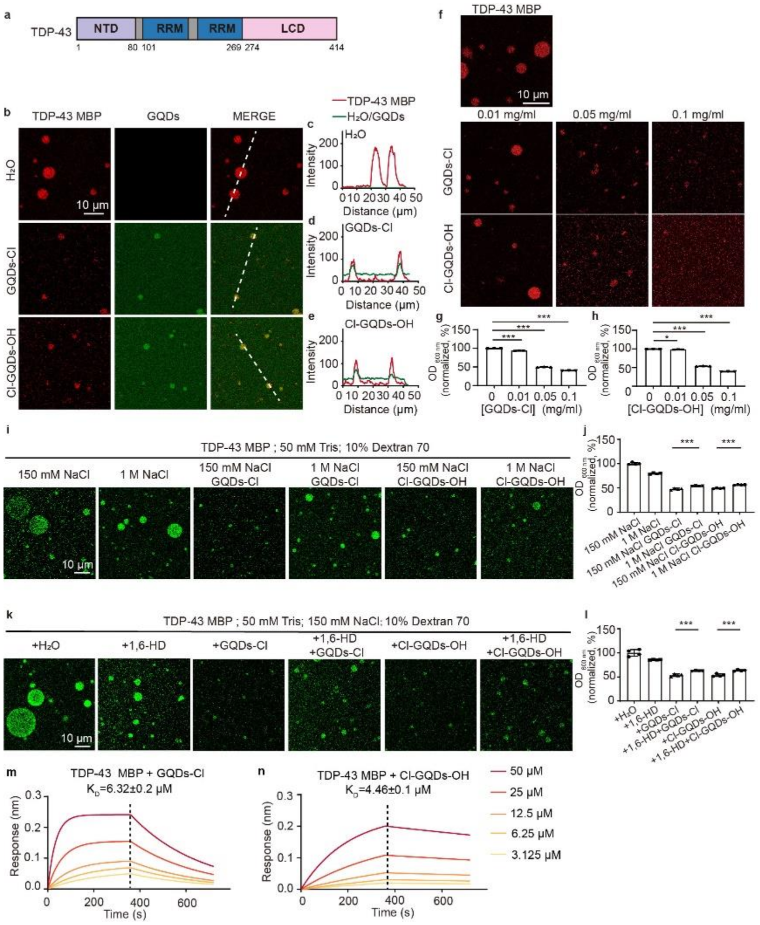

Figure 2. Halogen atom-doped GQDs inhibit TDP-43 phase separation *in vitro*. (a) Domain architecture of TDP-43. (b) Fluorescent images show TDP-43 MBP in the presence and absence of GQDs-Cl or Cl-GQDs-OH. TDP-43 MBP at a concentration of 20 μM undergoes LLPS within a buffer (50 mM Tris, pH 7.5, 150 mM NaCl, 10% Dextran 70), subsequently combined with 0.01 μg μL^{-1} GQDs-Cl or Cl-GQDs-OH. Scale bar, 10 μm. (c-e) Intensity profiles evaluating the colocalization of GQDs with TDP-43 MBP. (f) Fluorescence images provide representative views of 40 μM TDP-43 MBP undergoing phase separation in the presence of varying GQDs-Cl or Cl-GQDs-OH concentrations. Scale bar, 10 μm. (g-h) Turbidity measurements show the turbidity of TDP-43 MBP phase separation in the presence of GQDs-Cl or Cl-GQDs-OH. Data correspond to the mean ± SD, n = 3. Student's t test. *$P < 0.05$; ***$P < 0.001$. (i) Representative fluorescence images of 40 μM TDP-43 MBP undergoing phase separation with 50 mM Tris, pH 7.5, 1 M NaCl, 10% Dextran 70 and 0.1 μg μL^{-1} GQDs-Cl or Cl-GQDs-OH. Scale bar, 10 μm. (j) Turbidity measurement of 40 μM TDP-43 MBP as shown in image h. Data correspond to mean ± SD, n=4. Student's t test. ***$P < 0.001$. (k) Fluorescence image of TDP-43 MBP in the presence and absence of 1,6-HD and GQDs-Cl or Cl-GQDs-OH. Scare bar, 10 μm. (l) Turbidity measurement of 40 μM TDP-43 MBP as shown in image j. Data correspond to mean ± SD, n=4. Student's t test, ***$P<0.001$. (m-n) Kinetic binding curves of TDP-43 MBP with a concentration gradient of GQDs-Cl or Cl-GQDs-OH. The association and dissociation profiles are divided by a vertical black dotted line.

Abstract – 'decrease amyloid clumping' – unusual way to describe TDP-43 condensation, needs revision. Similarly, "irregular aggregation" is confusing.

Response: We thank the reviewer. In this revised abstract, I replaced "decrease amyloid clumping" with "inhibit the formation of TDP-43 amyloid-like aggregates" and "irregular aggregation" with "pathological aggregation" to provide clearer and more conventional descriptions of the TDP-43 protein behavior in disease states.

1. Gu J, Wang, C., Hu, R., Li, Y., Zhang, S., Sun, Y., Wang, Q., Li, D., Fang, Y., Liu, C. Hsp70 chaperones TDP-43 in dynamic, liquid-like phase and prevents it from amyloid aggregation. *Cell Res* **31**, 1024-1027 (2021).

2. Chen HJ, Topp, S. D., Hui, H. S., Zacco, E., Katarya, M., McLoughlin, C., King, A., Smith, B. N., Troakes, C., Pastore, A., Shaw, C. E. RRM adjacent TARDBP mutations disrupt RNA binding and enhance TDP-43 proteinopathy. *Brain* **142**, 3753-3770 (2019).

3. Zhang YJ*, et al.* Aberrant cleavage of TDP-43 enhances aggregation and cellular toxicity. *Proc Natl Acad Sci U S A* **106**, 7607-7612 (2009).

4. Pesiridis GS, Tripathy K, Tanik S, Trojanowski JQ, Lee VM. A "two-hit" hypothesis for inclusion formation by carboxyl-terminal fragments of TDP-43 protein linked to RNA depletion and impaired microtubule-dependent transport. *J Biol Chem* **286**, 18845-18855 (2011).

Reviewers' Comments:

Reviewer #1:
Remarks to the Author:
Specific requests were addressed.

Response to the reviewers 1:

Specific requests were addressed.

Response: We appreciate the reviewer's positive assessment of the revised manuscript and thank the reviewer for the constructive comments.
